# Negative regulation of NEMO signaling by the ubiquitin E3 ligase MARCH2

**DOI:** 10.15252/embj.2020105139

**Published:** 2020-09-16

**Authors:** Kiramage Chathuranga, Tae‐Hwan Kim, Hyuncheol Lee, Jun‐Seol Park, Jae‐Hoon Kim, Wijesinghe A Gayan Chathuranga, Pathum Ekanayaka, Youn Jung Choi, Chul‐Ho Lee, Chul‐Joong Kim, Jae U Jung, Jong‐Soo Lee

**Affiliations:** ^1^ College of Veterinary Medicine Chungnam National University Daejeon Korea; ^2^ Infectious Disease Research Center Korea Research Institute of Bioscience and Biotechnology Daejeon Korea; ^3^ California Institute for Quantitative Biosciences University of California Berkeley CA USA; ^4^ Laboratory Animal Resource Center Korea Research Institute of Bioscience and Biotechnology University of Science and Technology (UST) Daejeon Korea; ^5^ Department of Molecular Microbiology and Immunology Keck School of Medicine University of Southern California Los Angeles CA USA

**Keywords:** innate immunity, MARCH2, NEMO, ubiquitination, Immunology, Signal Transduction

## Abstract

NF‐κB essential modulator (NEMO) is a key regulatory protein that functions during NF‐κB‐ and interferon‐mediated signaling in response to extracellular stimuli and pathogen infections. Tight regulation of NEMO is essential for host innate immune responses and for maintenance of homeostasis. Here, we report that the E3 ligase MARCH2 is a novel negative regulator of NEMO‐mediated signaling upon bacterial or viral infection. MARCH2 interacted directly with NEMO during the late phase of infection and catalyzed K‐48‐linked ubiquitination of Lys326 on NEMO, which resulted in its degradation. Deletion of MARCH2 resulted in marked resistance to bacterial/viral infection, along with increased innate immune responses both *in vitro* and *in vivo*. In addition, MARCH2^−/−^ mice were more susceptible to LPS challenge due to massive production of cytokines. Taken together, these findings provide new insight into the molecular regulation of NEMO and suggest an important role for MARCH2 in homeostatic control of innate immune responses.

## Graphical abstract

MARCH2 interacts with the NF‐κB modulator NEMO and initiates its ubiquitin‐dependent degradation for homeostatic control of innate immune responses to viral and bacterial infection.

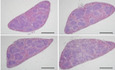

## Introduction

The innate immune system is the first line of host defense against viral and bacterial infections and functions by recognizing pathogen‐associated molecular patterns (PAMPs), which are usually components of viruses and bacteria (Akira *et al*, 2006). PAMPs are detected by host pattern recognition receptors (PRRs) such as Toll‐like receptors (TLRs) or RIG‐I‐like receptors (RLRs) (Creagh & O'Neill, 2006; Eyster *et al*, 2011; Lee *et al*, 2019). After sensing PRRs, a series of downstream signaling events facilitated by adaptor molecules is triggered, leading to production of type I interferons (IFNs) and pro‐inflammatory cytokines, and synthesis of antiviral interferon‐stimulated genes; this inhibits the spread of viruses and bacteria and activates adaptive immune responses (Sadler & Williams, 2008; Liu *et al*, 2011).

Upon viral or bacterial infection, the cellular signaling cascades that generate IFNs and pro‐inflammatory cytokines are triggered by molecules that activate transcriptional factors, including NF‐κB and IRFs (Heaton *et al*, 2016). Activation of NF‐κB is regulated by the IκBα kinase (IKK) complex (which is composed of the catalytic subunits IKKα and IKKβ) and the regulatory subunit IKKγ/NF‐κB essential modulator (NEMO). Upon activation of the IKK complex via K63‐linked polyubiquitination of NEMO, IκB is phosphorylated by IKKα and IKKβ, followed by its K48‐linked polyubiquitination and proteasomal degradation, thereby releasing NF‐κB, which then translocates to the cell nucleus to activate transcription of pro‐inflammatory cytokines and related genes (Heaton *et al*, 2016). Activation and nuclear translocation of IRF3 and IRF7 via the IKK‐related kinases TANK‐binding kinase (TBK1) and IKKε (Fitzgerald *et al*, 2003; Sharma *et al*, 2003) also occur in parallel; this leads ultimately to induction of type I interferons and other antiviral genes (McWhirter & Maniatis, 2005).

NEMO/IKKγ plays a key role in regulating both the NF‐κB and type I IFN signaling pathways (Xing *et al*, 2016). NEMO/IKKγ is the integral regulatory scaffolding protein of the canonical IKK complex (Häcker & Karin, 2006) and a key protein for cell survival and immune responses (Schmidt‐Supprian *et al*, 2000). For this reason, interrupting NEMO/IKKγ has severe consequences for tissue homeostasis, and mutation of NEMO/IKKγ can lead to hereditary human diseases such as incontinentia pigmenti, anhidrotic ectodermal dysplasia with immunodeficiency (EDA‐ID), and immunodeficiency 33 (Filipe‐Santos *et al*, 2006; Fusco *et al*, 2015).

By contrast, continuous or excessive activation of NEMO/IKKγ induces autoimmune disease, tumor development, and chronic inflammation (Ben‐Neriah & Karin, 2011; Ruland, 2011). Therefore, control of NEMO/IKKγ must be tightly regulated to maintain immune homeostasis. Recent studies identified both positive (mainly) and negative regulators of NEMO/IKKγ in the NF‐κB signaling pathway (Chariot *et al*, 2002; Field *et al*, 2003; Stilmann *et al*, 2009; Ashida *et al*, 2010; Xing *et al*, 2016; Zhang *et al*, 2017). However, other negative regulators of NEMO/IKKγ remain to be identified.

Membrane‐associated RING‐CH 2 (MARCH2), an E3 ubiquitin ligase, is a member of the MARCH family; this family comprises 11 members and localizes mainly to the endoplasmic reticulum (ER), Golgi, endosome, and plasma membrane (Nathan & Lehner, 2009). MARCH2 participates in vesicular trafficking between the trans‐Golgi network and endosomes, as well as in recycling of endosomes via interaction with syntaxin 6 (Nakamura *et al*, 2005). Deficiency of MARCH2 suppresses the growth of colon cancer cells by activating ER stress mechanisms (Xia *et al*, 2017). As an E3 ubiquitin ligase, MARCH2 transports ubiquitin to substrates such as DLG1, β2AR, CFTR, and ERGIC3, as well as to the envelope protein of HIV‐1 (Cao *et al*, 2008; Han *et al*, 2012; Cheng & Guggino, 2013; Zhang *et al*, 2018; Yoo *et al*, 2019). To date, however, the specific immunomodulatory function of MARCH2 upon viral or bacterial infection remains unknown.

In this study, we used MARCH2 knockout mice to demonstrate the physiological role of MARCH2 and showed that MARCH2 is a negative regulator of the NF‐κB and type I IFN signaling pathways upon viral or bacterial infection. MARCH2 interacts specifically with NEMO/IKKγ and mediates K48‐linked polyubiquitination and degradation of NEMO in response to infection. These findings suggest that interaction between MARCH2 and NEMO is essential for maintenance of homeostasis in the innate immune system.

## Results

### MARCH2 plays a critical role in host defense *in vivo*

The MARCH family of E3 ligases plays diverse roles in cell metabolism. These roles include membrane protein trafficking, antigen presentation, and regulation of innate immunity (Liu *et al*, 2019). However, its functions during innate immune responses are unclear, although we know that MARCH5 regulates MAVS (Yoo *et al*, 2015). Based on our preliminary MARCH family screening study, we identified MARCH2 as a potential negative regulator of the IFN‐β pathway.

**Figure EV1:**
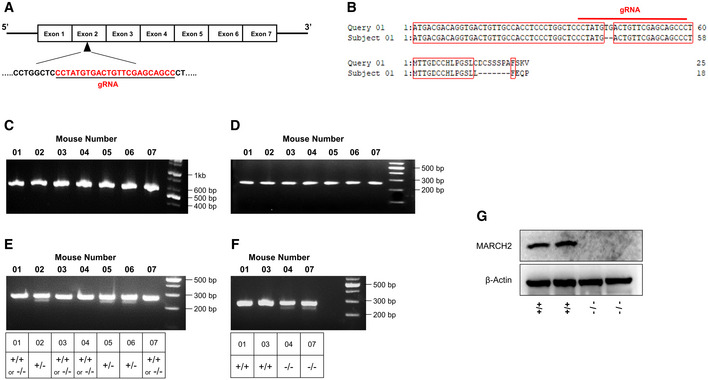
MARCH2 knockout mice generation ASchematic representation of the genomic target site in the MARCH2 gene and gRNA sequence.BSequencing alignment result of MARCH2 gene and mice genomic DNA.C–FGenotyping of MARCH2 mice (F2 generation). Heterozygous MARCH2^+/−^ mice (F1 generation) were mated, and genomic DNA was extracted from tail ends from the pups (F2 generation) (C, D) Agarose gel electrophoresis of nested PCR products. First PCR product size, 582 bp (C). Second PCR product size, 264 bp (D). (E) Agarose gel electrophoresis of melted, reannealed, and T7 endonuclease 1‐treated DNA. (F) MARCH2^+/+^ or MARCH2^−/−^ mice DNA was (Mouse number: 1, 3, 4, 7) individually mixed with equal amount of MARCH2^+/+^ DNA then, melted, and reannealed, and T7 endonuclease 1 was treated.GBMDMs isolated from MARCH2^+/+^ and MARCH2^−/−^ mice were infected with PR8‐GFP (MOI = 3) virus. Whole‐cell lysates were used for immunoblot with anti‐MARCH2 antibody, which is normalized by β‐actin.Data information: (+/+, wild‐type; +/−, heterozygous; −/−, knockout), gRNA: guide RNA.Source data are available online for this figure.

**Figure 1:**
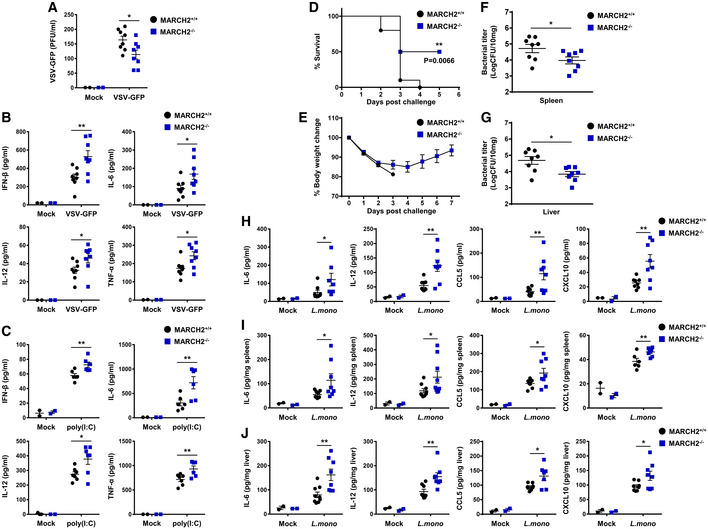
Deficiency of MARCH2 strengthens antimicrobial immunity *in vivo* A, BMARCH2^+/+^ (*n* = 8) or MARCH2^−/−^ (*n* = 8) mice were intravenously infected with VSV‐GFP (2 × 10^8^ PFU/mouse), and serum samples were collected at 12 h post‐infection (hpi). (A) Virus titer was analyzed in a plaque assay. (B) Secretion of IFN‐β, IL‐6, IL‐12, and TNF‐α was measured in specific ELISAs.CMARCH2^+/+^ (*n* = 6) or MARCH2^−/−^ (*n* = 6) mice were intravenously injected with poly(I:C) (200 μg/mouse). Serum samples were collected at 2 hpi, and secretion of IFN‐β, IL‐6, IL‐12, and TNF‐α was measured in specific ELISAs.D–JMARCH2^+/+^ (*n* = 8) or MARCH2^−/−^ (*n* = 8) mice were intraperitoneally challenged with 1 × 10^6 ^CFU of *Listeria monocytogenes*. (D) The percentage of surviving mice (D, log‐rank test, **P < 0.01) and body weight changes for each group (E) are shown. (F, G) At 3 dpc, the bacterial load in the spleen (F) and liver (G) was examined. (H–J) Amount of IL‐6, IL‐12, CCL5, and CXCL10 in serum (H), spleen (I), and liver (J) of mice in each group at 24 hpc.Data information: *P <* *0.05, **P <* *0.01 (two‐tailed Student's *t*‐test). Data are expressed as the mean ± SEM.Source data are available online for this figure.

To evaluate the potential roles of MARCH2 in innate immune responses, we generated MARCH2^−/−^ mice on a C57BL/6 background using the CRISPR‐Cas9 system and verified genetic knockout of the MARCH2 gene (Fig EV1A–G). MARCH2^−/−^ mice did not show any external abnormalities. First, to investigate the important role of MARCH2 in viral infection *in vivo*, we challenged MARCH2^+/+^ and MARCH2^−/−^ mice intravenously with VSV‐GFP (Fig 1A). The serum of MARCH2^−/−^ mice contained fewer replicating viruses than that of MARCH2^+/+^ mice. In addition, when we measured the amount of cytokines in the serum, we found that the concentrations of IFN‐β, IL‐6, IL‐12, and TNF‐α were higher in MARCH2^−/−^ mice than in MARCH2^+/+^ mice (Fig 1B). Furthermore, we measured serum cytokine levels in mice injected with the viral RNA mimic ligand, poly(I:C) (Fig 1C). As observed for virus infection, serum cytokine levels in MARCH2^−/−^ mice after ligand stimulation were higher than those in MARCH2^+/+^ mice. These results strongly suggest that MARCH2 is involved in antiviral innate immune responses *in vivo*.

Second, to investigate whether MARCH2 plays a role in response to bacterial infection, we challenged MARCH2^+/+^ and MARCH2^−/−^ mice with a lethal dose of *Listeria monocytogenes* (Lm). As shown in Fig 1D and E, MARCH2^−/−^ mice were more resistant to bacterial infection than MARCH2^+/+^ mice. To examine the effects on pathogen clearance, we examined the bacterial burden in the spleen and liver using colony‐forming unit (CFU) titration assays (Fig 1F and G). The results correlated with those of the survival assay; the bacterial load in MARCH2^−/−^ mice was significantly lower than that in MARCH2^+/+^ mice. Next, we investigated whether the low bacterial burden in MARCH2^−/−^ mice was due to higher inflammatory responses in these animals. Serum, spleen, and liver samples from MARCH2^−/−^ mice contained higher levels of IL‐6, IL‐12, CCL5, and CXCL10 than those from MARCH2^+/+^ mice (Fig 1H–J). These results also indicate that MARCH2 is involved in antibacterial innate immune responses *in vivo*.

**Figure 2:**
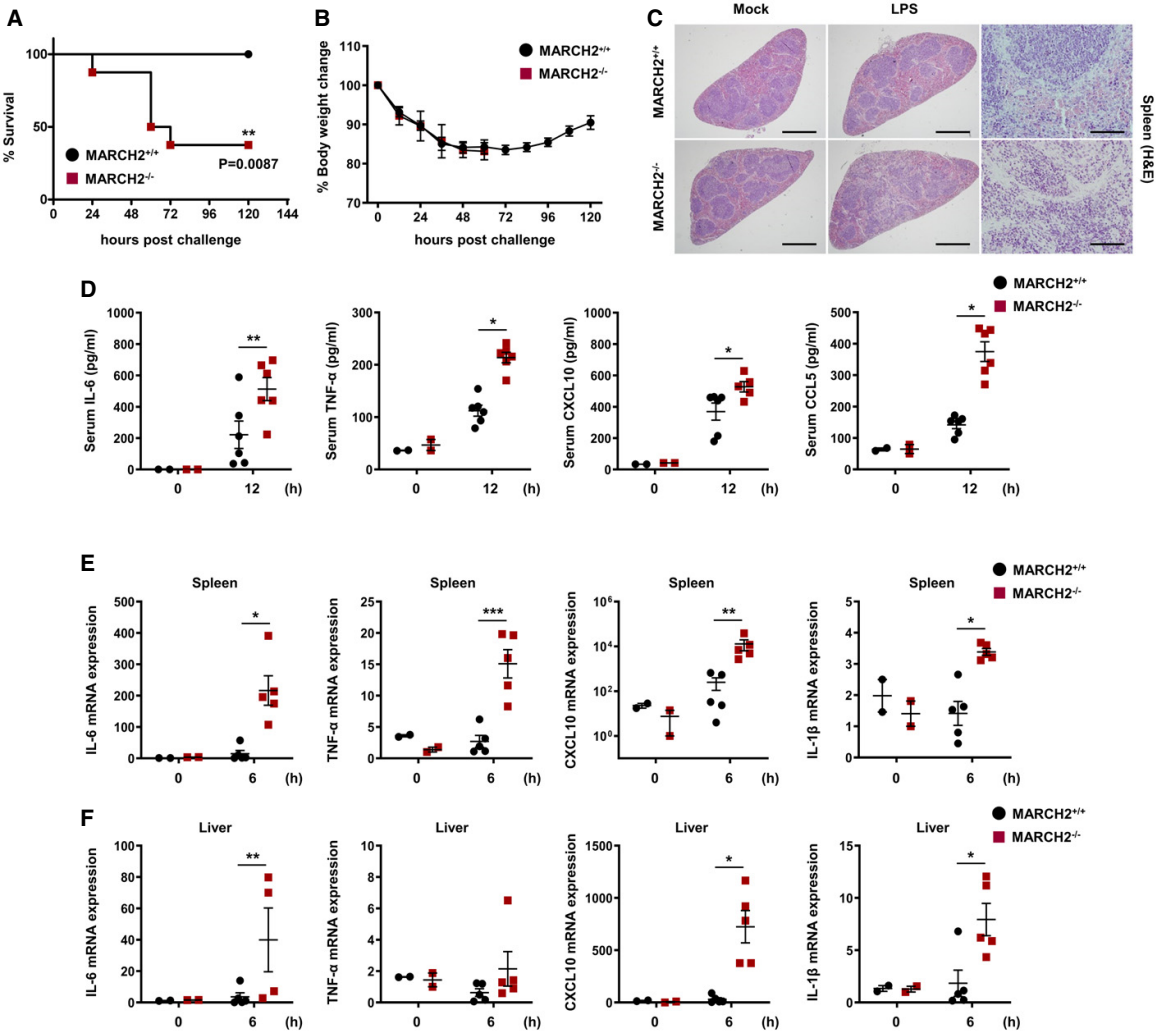
Deficiency of MARCH2 induces susceptibility to LPS‐induced septic shock A, BMARCH2^+/+^ (*n* = 8) or MARCH2^−/−^ (*n* = 8) mice were challenged intraperitoneally with LPS (24 mg/kg). (A, B) Percentage of surviving mice (A, log‐rank test, **P < 0.01) and body weight changes (B) in each group.CMARCH2^+/+^ (*n* = 4) or MARCH2^−/−^ (*n* = 4) mice were challenged intraperitoneally with LPS (24 mg/kg). Representing slides of H&E staining of spleen sections from each group. Scale bar, 6 mm.DMARCH2^+/+^ (*n* = 6) or MARCH2^−/−^ (*n* = 6) mice were challenged intraperitoneally with LPS (24 mg/kg). Levels of IL‐6, TNF‐α, CXCL‐10, and CCL‐5 in serum from mice in each group were measured at 12 hpc by ELISA.E, FMARCH2^+/+^ (*n* = 5) or MARCH2^−/−^ (*n* = 5) mice were challenged intraperitoneally with LPS (24 mg/kg). cDNA was prepared from total RNA extracted from spleen and liver of mice. Expression of mRNA encoding IL‐6, TNF‐α, CXCL10, and IL‐1β in spleen (E) and liver (F) from mice in each group was examined at 6 hpc by qPCR.Data information: *P <* *0.05, **P <* *0.01, ***P <* *0.001 (two‐tailed Student's *t‐*test). Data are expressed as the mean ± SEM.Source data are available online for this figure.

Third, to investigate whether MARCH2 is responsible for susceptibility to lipopolysaccharide (LPS)‐induced septic shock *in vivo*, MARCH2^+/+^ and MARCH2^−/−^ mice were injected intraperitoneally with LPS. All MARCH2^+/+^ mice survived, whereas over 60% of MARCH2^−/−^ mice died (Fig 2A and B). These results suggest that MARCH2^−/−^ mice are more susceptible to endotoxin shock than MARCH2^+/+^ mice. Furthermore, hematoxylin and eosin staining of spleen sections revealed greater inflammatory cell infiltration in MARCH2^−/−^ mice than in MARCH2^+/+^ mice (Fig 2C). Therefore, we hypothesized that MARCH2 is involved in inflammatory responses during LPS‐mediated septic shock. To test this, we used an ELISA to measure cytokine levels in serum (Fig 2D). As expected, MARCH2^−/−^ mice showed markedly higher expression of inflammatory mediators than MARCH2^+/+^ mice. We also measured expression of mRNA encoding pro‐inflammatory cytokine/chemokine genes in the spleen and liver at 6 hpt. As shown in Fig 2E and F, expression of mRNA encoding IL‐6, TNF‐α, CXCL10, and IL‐1β was significantly higher in MARCH2^−/−^ mice than in MARCH2^+/+^ mice. These data indicate that MARCH2 attenuates inflammatory responses under conditions of excessive inflammation. Collectively, the results provide *in vivo* evidence that MARCH2 is a critical regulator in innate immune responses against viral and bacterial infection.

### MARCH2 negatively regulates pathogen‐mediated innate immune responses

**Figure 3:**
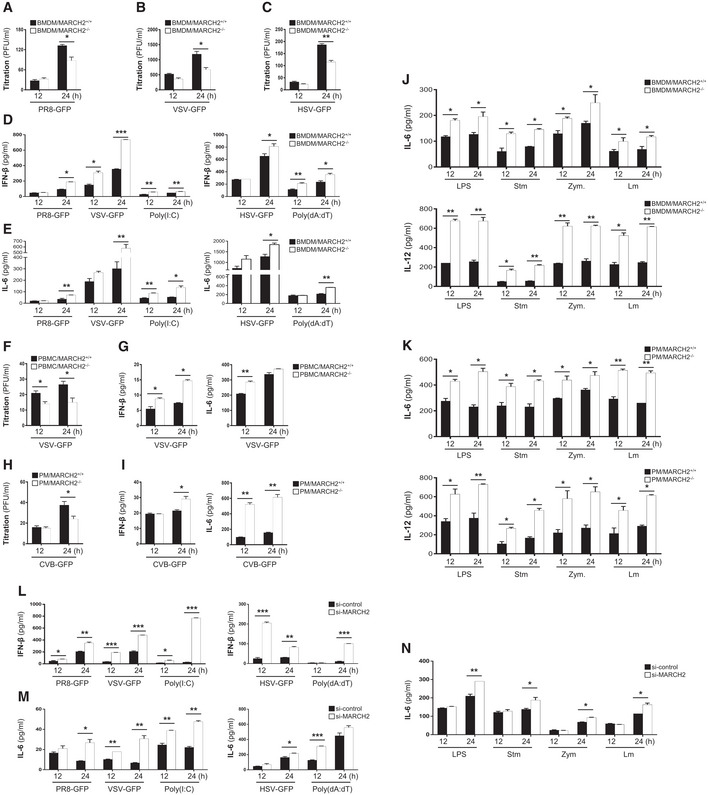
Deficiency of MARCH2 leads to augmented innate immune responses upon microbial infection in immune cells A–CBMDMs isolated from MARCH2^+/+^ or MARCH2^−/−^ mice were infected with PR8‐GFP (A, MOI = 3), VSV‐GFP (B, MOI = 5), or HSV‐GFP (C, MOI = 3), and virus titer was measured in a plaque assay.D, EBMDMs isolated from MARCH2^+/+^ or MARCH2^−/−^ mice were infected with viruses or treated with poly(I:C) (80 μg/ml) or poly (dA:dT) (1 μg/ml). The concentration of secreted IFN‐β (D) and IL‐6 (E) in the supernatants was measured in an ELISA.F, GPBMCs isolated from whole peripheral blood from MARCH2^+/+^ or MARCH2^−/−^ mice were infected with VSV‐GFP (MOI = 1). (F) The virus titer was measured in a plaque assay. (G) Concentration of IFN‐β and IL‐6 in the supernatant was measured in an ELISA.H, IPMs isolated from MARCH2^+/+^ or MARCH2^−/−^ mice infected with CVB‐GFP (MOI = 3). (H) Virus titer was measured in a plaque assay. (I) Concentration of IFN‐β and IL‐6 in the supernatant was measured in an ELISA.J, KBMDMs (J) or PMs (K) isolated from MARCH2^+/+^ or MARCH2^−/−^ mice were infected with *Salmonella typhimurium* or *Listeria monocytogenes*, or treated with LPS or zymosan. The concentration of IL‐6 and IL‐12 in supernatant was analyzed in an ELISA.L, MRAW 264.7 cells transfected with control siRNA (si‐control) or MARCH2‐specific siRNA (si‐MARCH2) were infected with PR8‐GFP (MOI = 1), VSV‐GFP (MOI = 0.5), poly(I:C) (20 μg/ml) or HSV‐GFP (MOI = 1) poly (dA:dT) (1 μg/ml), and IFN‐β (L), and IL‐6 (M) levels in the supernatant were measured in an ELISA.NRAW 264.7 cells transfected with control siRNA (si‐control) or MARCH2‐specific siRNA (si‐MARCH2) were infected with *S. typhimurium* or *L. monocytogenes*, or treated with LPS (100 ng/ml) or zymosan (100 μg/ml), and IL‐6 secretion into the cell supernatant was measured in an ELISAData information: *P <* *0.05, **P <* *0.01, ***P <* *0.001 (two‐tailed Student's *t‐*test). Data are representative of three independent experiments, each with similar results, and expressed as the mean ± SD of two biological replicates. Source data are available online for this figure.

To further assess the critical role of MARCH2 in virus‐ or bacteria‐mediated innate immune responses, we isolated bone marrow‐derived macrophages (BMDMs), peripheral blood mononuclear cells (PBMCs), and peritoneal macrophages (PMs) from MARCH2^+/+^ and MARCH2^−/−^ mice. First, we infected BMDMs with a RNA virus (influenza A virus [PR8‐GFP] or vesicular stomatitis virus [VSV‐GFP]) and a DNA virus (herpes simplex virus [HSV‐GFP]) and found that virus titers in MARCH2^−/−^ BMDMs were lower than those in MARCH2^+/+^ BMDMs (Fig 3A–C). Next, we used an ELISA to measure the amount of IFN‐β and IL‐6 secreted by BMDMs either infected with viruses or treated with poly(I:C) and poly(dA:dT). Consistent with the results of *in vivo* experiments, we found that MARCH2^−/−^ cells secreted more cytokines than MARCH2^+/+^ cells (Fig 3D and E), suggesting that MARCH2 deficiency increases production of type I IFNs and pro‐inflammatory cytokines and suppresses RNA virus infection of primary immune cells. Antiviral innate immune responses are initiated by host sensors, which activate key signaling molecules such as TBK1, IRF3, and P65. Since phosphorylation on specific amino acids within these molecules acts as an activation signal, we investigated phosphorylation in response to infection by VSV‐GFP. We found that the phosphorylation levels of these molecules in MARCH2^−/−^ BMDMs were markedly higher than those in MARCH2^+/+^ BMDMs (Appendix Fig S1A). Additionally, real‐time qPCR revealed that expression of mRNA encoding IFN‐β, IFN‐α, or other antiviral‐related genes was higher in MARCH2^−/−^ BMDMs (Appendix Fig S1B). Also, we found that PBMCs or PMs infected with VSV‐GFP or Coxsackievirus B (CVB‐GFP), respectively, showed similar phenotypes (Fig 3F–I). These results suggest that MARCH2 negatively regulates both the type I IFN signaling pathway and antiviral gene expression in primary immune cells in response to virus infection.

Second, to determine whether MARCH2 is involved in antibacterial innate immune responses, BMDMs or PMs from MARCH2^+/+^ and MARCH2^−/−^ mice were exposed to Lm, *Salmonella typhimurium* (Stm), Zymosan (Zym), or LPS, followed by measurement of IL‐6 and IL‐12 secretion into the culture supernatant at 12 h or 24 h. As shown in Fig 3J and K, stimulated MARCH2^−/−^ cells secreted more cytokines than MARCH2^+/+^ cells, a finding consistent with those from the virus infection experiments. Next, to examine the effect of MARCH2 on NF‐κB signaling, we treated MARCH2^+/+^ and MARCH2^−/−^ BMDMs with LPS and then subjected the cells to immunoblotting with the indicated antibodies (Appendix Fig S1C). The results showed that MARCH2 deficiency triggered increased phosphorylation of IkBα and NF‐κB. We also confirmed that stimulated MARCH2^−/−^ BMDMs showed higher expression of IL‐6 mRNA than stimulated MARCH2^+/+^ BMDMs (Appendix Fig S1D). Collectively, the data from primary cells suggest that MARCH2 negatively regulates virus‐ or bacteria‐mediated innate immune responses.

Third, to confirm the effects of MARCH2 *in vitro*, we prepared MARCH2 knockdown mouse macrophage cells (Raw264.7) by subjecting them to siRNA‐mediated gene silencing (Appendix Fig S2A). Similar to MARCH2^−/−^ BMDMs or PMs, virus replication in MARCH2 knockdown RAW264.7 cells was lower than that in control cells upon infection with PR8‐GFP, VSV‐GFP, or HSV‐GFP (Appendix Fig S2B–D). Also, we measured the concentration of IFN‐β and IL‐6 in the supernatant of cells infected with virus and in cells exposed to poly(I:C) and poly(dA:dT) (Fig 3L and M). We found that knockdown of MARCH2 increased secretion of IFN‐β and IL‐6. In addition, we confirmed similar activation of signaling molecules and induction of mRNA encoding IFN‐β and antiviral‐related genes in response to PR8‐GFP infection (Appendix Fig S2E and F). To evaluate antibacterial innate immune responses, we infected control and MARCH2 knockdown cells with Stm and Lm or treated them with LPS and Zym. As shown in Fig 3N, secretion of IL‐6 by MARCH2 knockdown cells was higher than that by control cells; knockdown of MARCH2 increased expression of mRNA encoding inflammatory cytokines and antimicrobial genes in response to LPS stimulation (Appendix Fig S2G). Next, to confirm these results, we generated stable MARCH2‐overexpressing RAW264.7 cells and confirmed overexpression by immunoblot analysis (Appendix Fig S3A). As in the previous experiments, control cells and MARCH2‐overexpressing cells were infected with viruses, followed by measurement of GFP expression, virus titers, and IFN‐β or IL‐6 concentrations. Overexpression of MARCH2 increased virus replication and reduced secretion of IFN‐β and IL‐6 in response to infection by VSV‐GFP, PR8‐GFP, or HSV‐GFP (Appendix Fig S3B–G). Activation of signaling molecules in MARCH2‐overexpressing cells was lower than that in control cells (Appendix Fig S3H). We also examined the physiological role of MARCH2 in antibacterial innate immune responses by exposing MARCH2‐overexpressing Raw264.7 cells to diverse bacteria and TLR ligands. To do this, cells were infected with Stm or Lm or treated with LPS, Zym, imiquimod, or CpG oligodeoxynucleotides (CpG‐ODN). We found that secretion of IL‐6 and IL‐12 into the supernatant of MARCH2‐overexpressing cells in response to all bacteria and ligands was lower than that into the supernatant of control cells (Appendix Fig S3I and J), confirming the function of MARCH2 in antibacterial innate immune responses. Taken together, the data strongly suggest that MARCH2 plays a negative regulatory role in virus‐ or bacteria‐mediated innate immune responses.

**Figure EV2:**
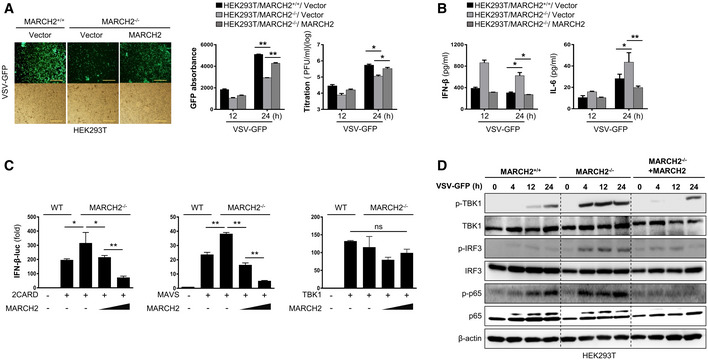
Reconstitution of MARCH2 in MARCH2^−/−^ HEK293T cells reduces the immune response leading to increased virus replication A, BMARCH2^+/+^ HEK293T cells transfected with Flag‐tagged empty vector or MARCH2^−/−^ HEK293T cells transfected with Flag‐tagged empty vector or MARCH2^WT^ were infected with VSV‐GFP (MOI = 0.5). (A) Viral replication was determined at 24 hpi by fluorescence microscopy, fluorescence absorbance, and plaque assay. (B) Concentration of IFN‐β and IL‐6 secreted in supernatants was determined at 12 and 24 hpi by ELISA, Scale bar, 50 μm.CIFN‐β luciferase reporter assay. MARCH2^+/+^ or MARCH2^−/−^ HEK293T cells were transfected with firefly luciferase reporter plasmid encoding INF‐β promoter, TK‐Renilla plasmid, and expression plasmids of RIG‐I 2CARD, MAVS, or TBK‐1. MARCH2^−/−^ HEK293T cells were reconstituted with increasing amount of Flag‐tagged MARCH2 plasmid (100, 200 ng).DMARCH2^+/+^, MARCH2^−/−^, or MARCH2^−/−^ HEK293T reconstituted with Flag‐tagged MARCH2^WT^ were infected with VSV‐GFP (MOI = 0.5). Cells were harvested at indicated hours post‐infection; total and phosphorylated TBK1, IRF3, and p65 were measured by immunoblotting in whole‐cell lysates. β‐actin was used to confirm equal loading of protein.Data information: *P <* *0.05, **P <* *0.01 (two‐tailed Student's *t‐*test). Data are representative of at least two independent experiments, each with similar results, and expressed as the mean ± SD of two biological replicates. Source data are available online for this figure.

To exclude the possibility that negative regulation of pathogen‐mediated innate immune signaling by MARCH2 is a cell type‐specific phenomenon, we examined the function of MARCH2 in HEK293T cells. Upon infection with PR8‐GFP virus, HEK293T cells transiently expressing MARCH2 showed higher susceptibility to virus infection (Appendix Fig S4A–C). Next, we generated a MARCH2^−/−^ HEK293T cell line to test whether regulatory function was restored by reconstitution of MARCH2. MARCH2^+/+^, MARCH2^−/−^, and MARCH2^−/−^ cells transiently transfected with MARCH2 were infected with VSV‐GFP. Virus titration and cytokine secretion assays showed that reconstitution of MARCH2 restored its innate immune function (Fig EV2A and B). These results were confirmed in a luciferase assay and by immunoblot analysis of activated signaling molecules (Fig EV2C and D). Taken together, these results suggest that MARCH2 is a negative regulator of both antiviral and antibacterial innate immune responses.

### MARCH2 interacts with NEMO upon pathogen infection

**Figure 4:**
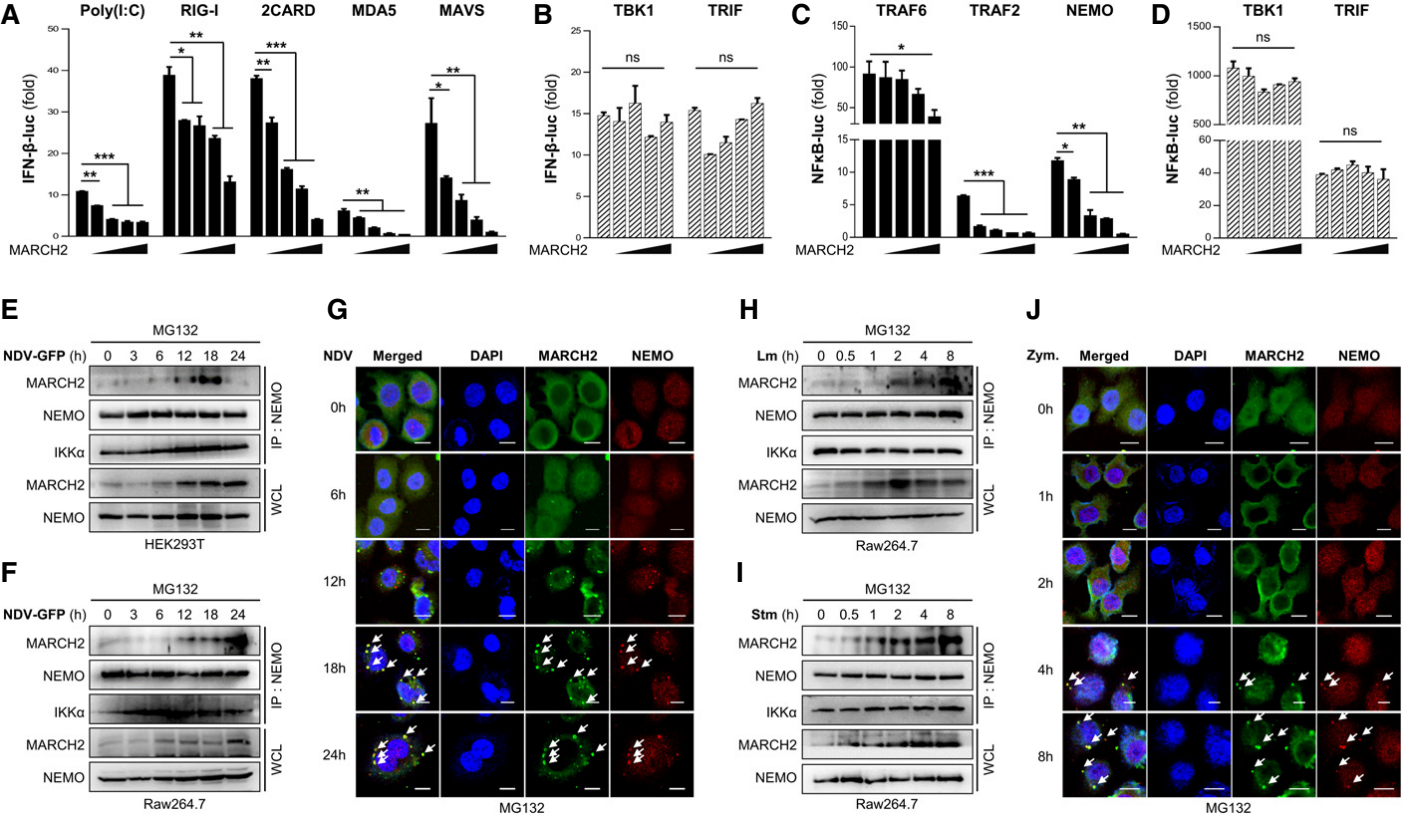
MARCH2 negatively regulates innate immunity by interacting with NEMO A–DHEK293T cells were transfected with a firefly luciferase reporter plasmid encoding the IFN‐β promoter (A, B) or the NF‐κB promoter (C, D), plus a TK‐Renilla plasmid and an increasing dose of flag‐tagged MARCH2 plasmid (50, 100, 200, 400 ng) plus expression plasmids for RIG‐I 2CARD, RIG‐I, MDA‐5, MAVS, TBK1, TRAF 6, TRAF 2, NEMO, or TRIF, or stimulated with poly(I:C), for 24 h. Results are expressed relative to those of Renilla luciferase alone (internal control).E, FInteraction between MARCH2 and NEMO in response to viral infection. HEK293T cells (E) or Raw264.7 cells (F) were infected with NDV‐GFP in a time‐dependent manner in the presence of MG132 (proteasome inhibitor, 10 μM). Cell lysates were subjected to immunoprecipitation with an anti‐NEMO antibody, followed by immunoblotting with an anti‐MARCH2 antibody.GConfocal microscopy was conducted to examine time‐dependent co‐localization of MARCH2 and NEMO in HeLa cells upon NDV infection (MOI = 1) in the presence of MG132 (10 μM). Arrow indicates the co‐localized NEMO and MARCH2 protein.H, IInteraction between MARCH2 and NEMO in response to bacterial infection. Raw264.7 cells were infected with *Listeria monocytogenes* (H) or *S. typhimurium* (I) in a time‐dependent manner in the presence of MG132. Cell lysates were subjected to immunoprecipitation with an anti‐NEMO antibody, followed by immunoblotting with an anti‐MARCH2 antibody.JConfocal microscopy was conducted to examine time‐dependent co‐localization of MARCH2 and NEMO in HeLa cells expressing TLR2 upon zymosan treatment (100 μg/ml) in the presence of MG132 (10 μM). Arrow indicates the co‐localized NEMO and MARCH2 protein.Data information: Scale bar (G, J), 10 μm. ns, not significant. *P <* *0.05, **P <* *0.01, ***P <* *0.001 (two‐tailed Student's *t‐*test). Data are representative of at least two independent experiments, each with similar results, and are expressed as the mean ± SD of three biological replicates. Source data are available online for this figure.

**Figure EV3:**
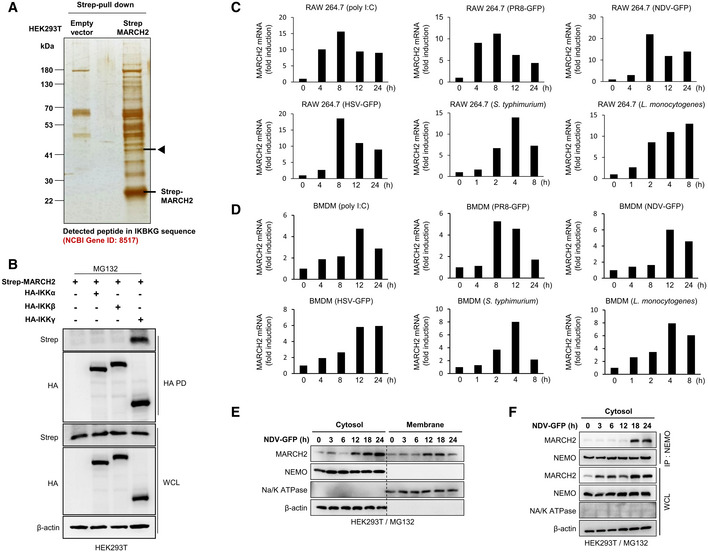
MARCH2 is expressed by pathogen infection and interacts with NEMO in cytosol ASilver staining for MARCH2 interactome assay. Arrow indicates the expected protein expression of NEMO.BHEK293T cells transfected with Strep‐tagged MARCH2 together with HA‐tagged IKKα, IKKβ, or NEMO under MG132 treatment were subjected to immunoprecipitation with an anti‐HA antibody, followed by immunoblot with anti‐Strep antibody or anti‐HA antibody. Whole‐cell lysates were determined by immunoblotting with anti‐Strep, anti‐HA, or anti‐β‐actin antibody.C, DMARCH2 expression upon virus and bacterial infection. RAW 264.7 cells (C) and BMDMs isolated from C57BL/6 mice (D) were treated with poly I:C or infected with PR8‐GFP, NDV‐GFP, HSV‐GFP, *S. typhimurium*, and *Listeria monocytogenes*, followed by qPCR with MARCH2 gene‐specific primers.E, FHEK293T cells were infected with NDV‐GFP (MOI = 1) under MG132 treatment and harvested at indicated time points. (E) Protein expression in cytosolic and total membrane fraction was determined by immunoblotting with anti‐MARCH2, anti‐NEMO, anti‐Na/K ATPase, or anti‐β‐actin antibody. (F) Cytosolic fraction was subjected to immunoprecipitation with an anti‐NEMO antibody, followed by immunoblotting with anti‐MARCH2 or anti‐NEMO antibody. Protein expression in total cytosolic fraction was determined by immunoblotting with anti‐MARCH2, anti‐NEMO, anti‐Na/K ATPase, or anti‐β‐actin antibody.Data information: Data are representative of at least two independent experiments, each with similar results (B–F). Source data are available online for this figure.

Upon viral or bacterial infection, host sensors activate signaling cascades. Above, we showed that MARCH2 affects these signaling pathways (Fig EV2D and Appendix Figs S1A and C, S2E, and S3H). To identify the step in the type I IFN and NF‐κB cellular signaling cascade that is regulated by MARCH2, we performed a luciferase promoter assay by co‐expressing MARCH2 along with several factors. We found that MARCH2 reduced 2CARD‐, RIG‐I‐, MDA5‐, MAVS‐, and poly(I:C)‐mediated activation of the IFN‐β promoter in dose‐dependent manner (Fig 4A). However, there was no detectable change in TBK1‐mediated promoter activity with increasing expression of MARCH2 (Fig 4B), suggesting that MARCH2 targets the molecule(s) immediately upstream of TBK1. Because MARCH2 deficiency affects LPS‐mediated phosphorylation of IκBα and p65 (Appendix Fig S1C), we conducted a NF‐κB promoter–luciferase assay by co‐expressing MARCH2 and other molecules belonging to the NF‐κB signaling pathway. As expected, the NF‐κB promoter assay revealed that MARCH2 reduced TRAF6, TRAF2, and NEMO‐mediated activation of the NF‐κB promoter (Fig 4C), but not TBK‐mediated activation (Fig 4D). Both the TLR‐ and RLR‐mediated signaling pathways activate the canonical IKK complex and TBK1 to trigger innate immune responses. Therefore, these data suggest that MARCH2 may control the IKK complex or related downstream pathways. To further identify a specific target molecule of MARCH2 during innate immune responses, we expressed Strep‐tagged MARCH2 in HEK293T cells and conducted a large‐scale Strep pull‐down assay (Fig EV3A). Mass spectrometry analysis identified NEMO as a candidate target of MARCH2, leading us to hypothesize that MARCH2 interacts with NEMO. To check this, we performed an immunoprecipitation assay using Strep‐tagged MARCH2 and each HA‐tagged IKK molecule. Because MARCH2 is an E3 ligase, all binding assays were performed in the presence of MG132 to prevent proteasomal degradation. We found that MARCH2 interacted with NEMO but not with IKKα and IKKβ (Fig EV3B).

Next, we investigated the interaction between endogenous MARCH2 and NEMO over time in response to viral or bacterial infection. Immunoprecipitation experiments revealed endogenous binding of MARCH2 to NEMO in HEK293T and Raw264.7 cells after infection with Newcastle disease virus (NDV‐GFP; Fig 4E and F). A similar result was obtained in an immunoprecipitation assay in HEK293T cells infected with PR8‐GFP and HSV‐GFP (Appendix Fig S5A and B). We also used confocal microscopy to confirm time‐dependent localization of MARCH2 and NEMO. After infection of HeLa cells with NDV, confocal microscopy revealed co‐localization of both molecules at a late stage post‐infection (Fig 4G). Likewise, immunoprecipitation and confocal microscopy revealed an endogenous interaction between MARCH2 and NEMO in Raw264.7 cells in response to Lm or Stm (Fig 4H and I), and in TLR2‐expressing HeLa cells after Zym treatment (Fig 4J). Moreover, a similar interaction was shown clearly in A549 cells treated with LPS (Appendix Fig S5C). These interactions also occurred at a late stage after bacterial infection. Notably, the expression level of MARCH2 protein was up‐regulated, and the interactions occurred at a late stage after virus infection. To confirm up‐regulation of MARCH2 expression and late‐stage interaction between MARCH2 and NEMO, we measured induction of endogenous mRNA encoding MARCH2 in a time‐dependent manner. Expression of MARCH2 mRNA increased in RAW264.7 cells and BMDMs following viral or bacterial infection (Fig EV3C and D), which correlated with expression levels of MARCH2 protein (Fig EV3E–G). Previous reports identified the main cellular location of MARCH2 as the plasma membrane, ER, and lysosomal or endosomal vesicles (Nakamura *et al*, 2005). Thus, to identify the site of interaction between MARCH2 and NEMO, we assessed the intracellular localization of MARCH2 using confocal microscopy and cell fractionation assays after pathogen infection. Interestingly, we found that a substantial amount of MARCH2 did not localize to the ER or lysosome upon virus infection or zymosan stimulation (Appendix Fig S6 and S7). As shown in Fig EV3E, expression of MARCH2 protein was markedly higher in the cytoplasmic fraction than in the membrane fraction at the late phase of virus infection; in addition, interaction between MARCH2 and NEMO was observed in the cytosolic fraction (Fig EV3F).

Taken together, these results suggest that MARCH2 binds to NEMO directly during the late stages of viral or bacterial infection and negatively regulates innate immunity to maintain cellular homeostasis.

### MARCH2 mediates degradation of NEMO via K48‐linked polyubiquitination

**Figure 5:**
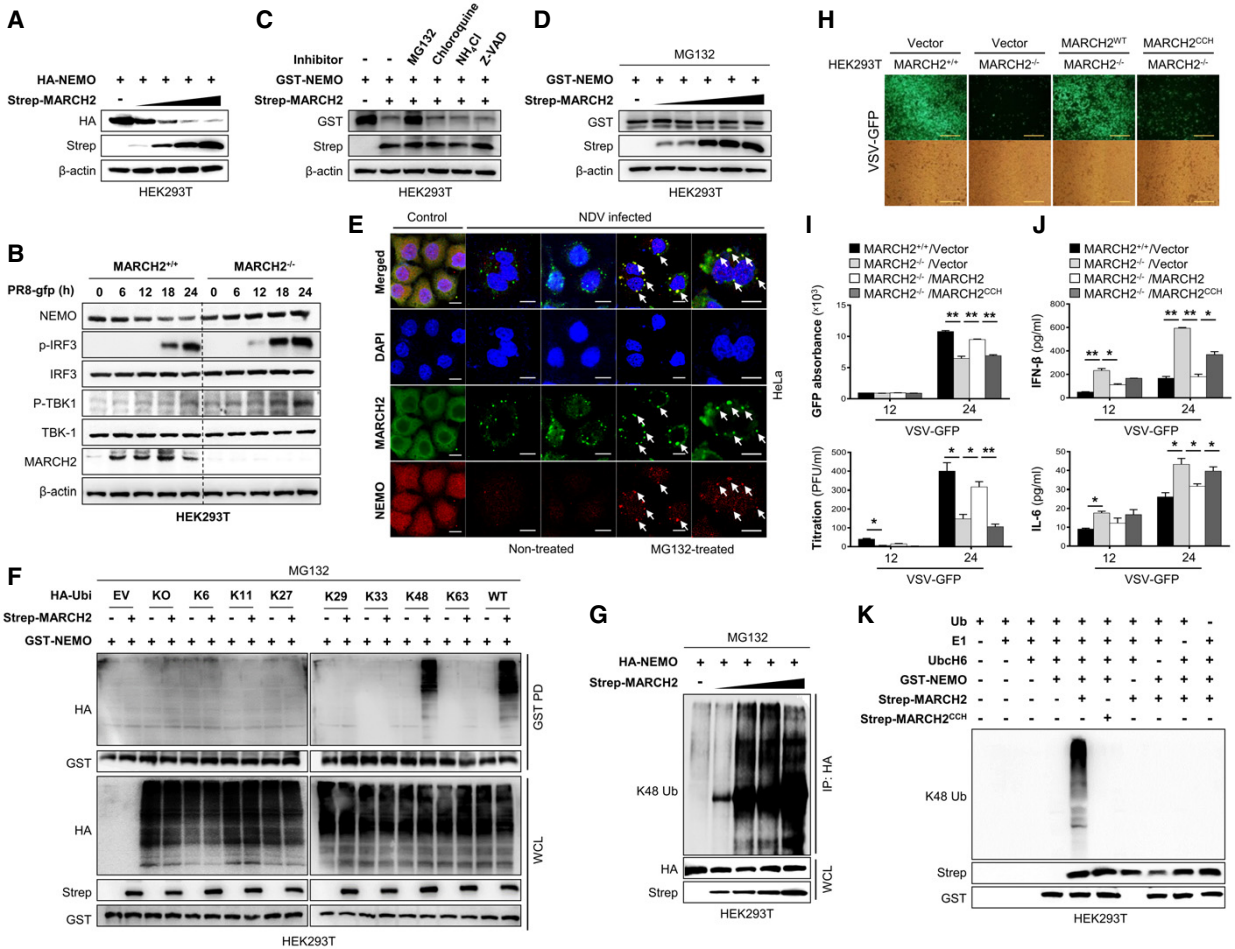
MARCH2 leads to proteasomal degradation of NEMO by mediating K48‐linked polyubiquitination A, BNEMO degradation assay. (A) HEK293T cells were transfected with different doses of Strep‐tagged MARCH2 in the presence of HA‐tagged NEMO plasmid. (B) MARCH2^+/+^ and MARCH2^−/−^ HEK293T cells were infected with PR8‐GFP (MOI = 1) in time‐dependent manner. Cells were harvested following infection; NEMO expression level, total and phosphorylated IRF‐3, and TBK‐1 were measured by immunoblotting. β‐actin was used to confirm equal loading of protein.CHEK293T cells transfected with a Strep‐tagged empty vector or MARCH2 together with GST‐tagged NEMO were treated with MG132 (10 μM), chloroquine, NH_4_Cl, or Z‐VAD for 6 h before harvest. Whole‐cell lysates were subjected to immunoblotting with indicated antibodies.DHEK293T cells were transfected with different doses of Strep‐tagged MARCH2 together with GST‐tagged NEMO plasmid. MG132 (10 μM) was added to cells for 6 h prior to harvest. Whole‐cell lysates were subjected to immunoblotting with the indicated antibodies.EConfocal microscopy was used to examine co‐localization of MARCH2 and NEMO in HeLa cells upon NDV infection (MOI = 1) in the presence or absence of MG132 (10 μM). Scale bar, 10 μm. Arrow indicates the co‐localized NEMO and MARCH2 protein.FHEK293T cells transfected with Strep‐tagged empty vector or MARCH2 with GST‐tagged NEMO and each different HA‐tagged ubiquitin mutants (indicated lysine (K) only, other lysines (K) mutated to arginines (R)) were treated with MG132 (10 μM) for 6 h before harvest. Lysates were subjected to pull‐down with GST beads, followed by immunoblotting with an anti‐HA antibody. Whole‐cell lysates were determined by immunoblotting with the indicated antibodies.GNEMO ubiquitination assay. HEK293T cells were transfected with different doses of Strep‐tagged MARCH2 in the presence of a HA‐tagged NEMO plasmid. Lysates were immunoprecipitated with anti‐HA antibody followed by immunoblotting with anti‐K48 antibody.H–JMARCH2^+/+^ HEK293T cells harboring empty vector or MARCH2^−/−^ HEK293T cells harboring empty vector, Flag‐tagged MARCH2, or MARCH2^CCH^ (a mutant harboring mutations in residues within the catalytic domain: C64S, C67S, and H90Q) were infected with VSV‐GFP (MOI = 0.05). Viral replication was assessed under a fluorescence microscope (H) or by measuring GFP expression using a fluorescence modulator (I, top) and in a plaque assay (I, bottom). (J) Levels of IFN‐β (top) and IL‐6 (bottom) in cell supernatants were measured in an ELISA.K
*In vitro* ubiquitination assay for NEMO. HEK293T cells were transfected with GST‐tagged NEMO, Strep‐tagged MARCH2, or MARCH2^CCH^ individually. Immunoprecipitates were incubated with a reaction mixture containing ubiquitin, E1, and UbcH6 (E2), followed by immunoblotting with an anti‐K48 linkage‐specific polyubiquitin antibody.Data information: *P <* *0.05, **P <* *0.01 (two‐tailed Student's *t‐*test). Data are representative of at least two independent experiments, each with similar results, and are expressed as the mean ± SD of three biological replicates. Source data are available online for this figure.

**Figure EV4:**
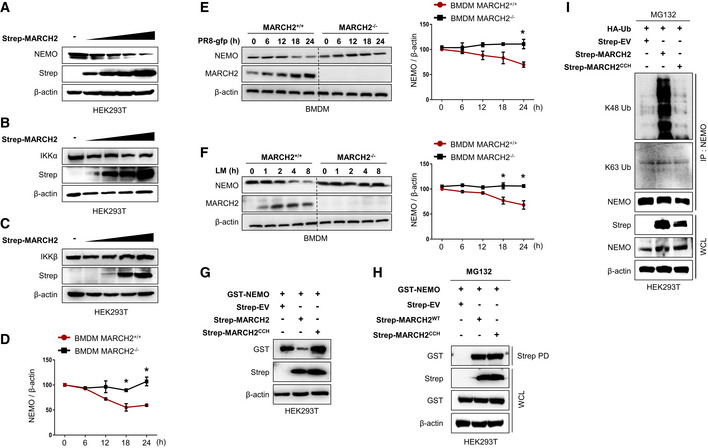
MARCH2 induces NEMO degradation via E3 ubiquitin ligase function A–CHEK293T cells were transfected with an increasing amount of Strep‐tagged MARCH2, followed by immunoblot with anti‐NEMO (A), anti‐IKKα (B), or anti‐IKKβ (C) and the other indicated antibodies.DArbitrary value of band intensity of NEMO blot shown in Fig 5B.E, FMARCH2^+/+^ and MARCH2^−/−^ BMDMs were infected with PR8‐GFP (E, MOI = 3) or *Listeria monocytogenes* (F, MOI = 1) in time‐dependent manner. Cells were lysed, where NEMO and MARCH2 expression was determined by immunoblotting. β‐actin was used to confirm equal loading of protein. Band intensity of NEMO blots was shown in graphs (right).GHEK293T cells were transfected with a Strep‐tagged empty vector, MARCH2, or MARCH2^CCH^ together with GST‐tagged NEMO. Whole‐cell lysates were subjected to immunoblotting with the indicated antibodies.HHEK293T cells transfected with Strep‐tagged empty vector, MARCH2, or MARCH2^CCH^ together with GST‐tagged NEMO were subjected to pull‐down with Strep beads, followed by immunoblotting with anti‐GST antibody. Whole‐cell lysates were determined by immunoblotting with the indicated antibodies.IHEK293T cells transfected with a Strep‐tagged empty vector, MARCH2, or MARCH2^CCH^ together with HA‐tagged ubiquitin were treated with MG132 (10 μM) for 6 h before harvest. Whole‐cell lysates were subjected to immunoprecipitation with an anti‐NEMO antibody, followed by immunoblotting with anti‐K48 or anti‐K63 linkage‐specific polyubiquitin antibodiesData information: *P < 0.05 (two‐tailed Student's *t‐*test). Data are representative of at least two independent experiments, each with similar results, and expressed as the mean ± SD of two biological replicates. Source data are available online for this figure.

NEMO is a well‐known regulatory subunit of the IKK complex, which itself is an essential modulator in innate immunity. Until now, the results show that MARCH2 binds NEMO upon bacterial or viral infection and negatively regulates innate immune responses (Fig 4); therefore, we hypothesized that MARCH2 might utilize NEMO to maintain cell homeostasis by controlling innate immune signaling. MARCH2 is an E3 ubiquitin ligase; thus, we asked whether binding of NEMO by MARCH2 promotes proteasomal degradation of NEMO. First, we expressed MARCH2 together with NEMO in HEK293T cells and found that expression of NEMO fell in the presence of MARCH2 (Fig 5A). Likewise, endogenous expression of NEMO fell in a dose‐dependent manner upon ectopic expression of MARCH2 (Fig EV4A), while that of IKKα and IKKβ was unchanged (Fig EV4B and C). Moreover, viral infection (PR8‐GFP) decreased expression of endogenous NEMO protein in wild‐type but not in MARCH2 knockout HEK293T cells at late time points (Figs 5B and EV4D). Similar results were observed after viral (PR8‐GFP) and bacterial (Lm) infection of wild‐type and knockout BMDM cells (Fig EV4E and F). Next, we expressed NEMO and MARCH2 in cells treated with several inhibitors to investigate the possibility that NEMO is regulated by MARCH2‐mediated proteasomal degradation. Expression of NEMO was recovered in the presence of the proteasomal inhibitor MG132 but not in the presence of lysosomal inhibitors (chloroquine or NH_4_Cl) or a pan‐caspase inhibitor (Z‐VAD) (Fig 5C and D), suggesting that NEMO undergoes MARCH2‐induced proteasomal degradation. Additionally, we confirmed co‐localization of MARCH2 and NEMO, with or without MG132 treatment, after virus infection. As shown in Fig 5E, co‐localization of MARCH2 and NEMO occurred only in the presence of MG132.

Thus, we next asked whether NEMO degradation is mediated by ubiquitination. We expressed GST‐tagged NEMO and Strep‐tagged MARCH2 along with different mutants of ubiquitin and found that NEMO undergoes K48‐linked polyubiquitination by MARCH2 (Fig 5F). This result was also supported by an immunoprecipitation assay, showing that NEMO undergoes MARCH2‐mediated K48‐linked polyubiquitination, but not K63‐linked polyubiquitination, in a dose‐dependent manner (Fig 5G and Appendix Fig S8A). These findings indicate that MARCH2 mediates ubiquitin‐dependent proteasomal degradation of NEMO by conjugating K48‐linked polyubiquitin chains.

### Enzymatic activity of MARCH2 is essential for ubiquitination of NEMO

Previous reports identified three residues within the catalytic domain of MARCH2 that are essential for E3 ligase activity by coordinating zinc ions (Han *et al*, 2012). To evaluate whether MARCH2 E3 ligase activity affects NEMO ubiquitination and subsequent degradation during virus or bacterial infection, we generated a construct harboring mutations at cysteines 64 and 67 and at histidine 90 (C64S, C67S, and H90Q); this was designated MARCH2^CCH^. First, we infected HEK293T^MARCH2+/+^ cells and HEK293T^MARCH2−/−^ cells harboring MARCH2 or MARCH2^CCH^ with VSV‐GFP and then examined antiviral innate immune responses (Fig 5H and Appendix Fig S8b). As shown in Fig 5I and J, HEK293T^MARCH2−/−^ cells showed lower virus replication and higher cytokine secretion than HEK293T^MARCH2+/+^ cells; reconstitution of MARCH2 reversed this phenomenon. Interestingly, reconstitution of MARCH2^CCH^ had no effect on virus replication or cytokine secretion (Fig 5H–J). The results for Raw264.7 cells overexpressing MARCH2 or MARCH2^CCH^ and infected with VSV‐GFP were the same as those for the HEK293T^MARCH2−/−^ cell line (Appendix Fig S8C–E), suggesting that MARCH2 regulates innate immune responses via its E3 ligase activity. Next, we assessed the effect of MARCH2^CCH^ on NEMO. HEK293T cells were transfected with NEMO together with MARCH2 or MARCH2^CCH^ in the absence of MG132; as expected, expression of NEMO fell in the presence of MARCH2 but not in the presence of MARCH2^CCH^ (Fig EV4G). However, MARCH2^CCH^ bound to NEMO to the same extent as MARCH2 in the presence of MG132 (Fig EV4H). Next, we investigated K48‐linked polyubiquitination of NEMO in the presence of MARCH2 or MARCH2^CCH^. We found that MARCH2^CCH^ had no effect on NEMO ubiquitination. Expression of MARCH2^CCH^ did not trigger K48‐linked ubiquitination of ectopic or endogenous NEMO, whereas MARCH2 did (Fig EV4I and Appendix Fig S8F). These data indicate that the enzymatic function of MARCH2 is critical for K48‐linked polyubiquitination of NEMO. Finally, an *in vitro* ubiquitination assay was performed in the presence of NEMO, E1 and E2 enzymes, and ubiquitin, plus MARCH2 or MARCH2^CCH^. Consistent with the results presented in Fig EV4I and Appendix Fig S8F, K48‐linked polyubiquitination of NEMO was observed only in the presence of MARCH2 (Fig 5K). Thus, these results provide evidence that the E3 ubiquitin ligase activity of MARCH2 mediates K48‐linked polyubiquitination of NEMO, which in turn leads to its proteasomal degradation.

### MARCH2 conjugates K48‐linked polyubiquitin at lysine 326 of NEMO

**Figure 6:**
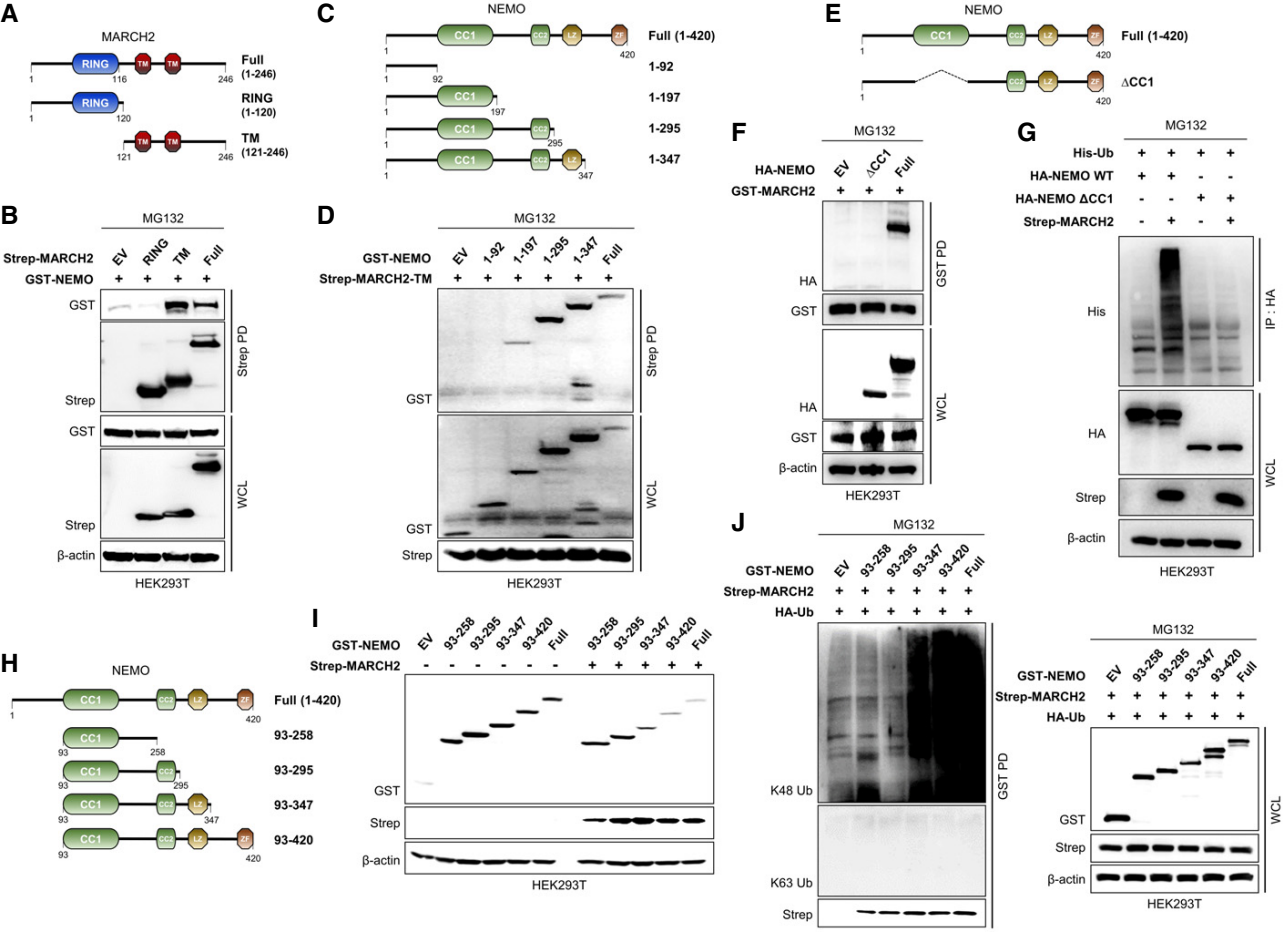
MARCH2 interacts with CC1 domain of NEMO via TM domain A, B(A) Schematic representations of the domain constructs of MARCH2. (B) HEK293T cells were transfected with Strep‐tagged MARCH2 domain constructs together with GST‐tagged NEMO. Whole‐cell lysates were subjected to a Strep‐PD assay, followed by immunoblotting with an anti‐GST antibody.C, D(C) Schematic representations of the domain constructs of NEMO. (D) HEK293T cells were transfected with GST‐tagged NEMO domain constructs together with Strep‐tagged MARCH2‐TM. Whole‐cell lysates were subjected to a Strep‐PD assay, followed by immunoblotting with an anti‐GST antibody.E, F(E) Schematic representations of the deletion mutant constructs of NEMO. (F) HEK293T cells were transfected with HA‐tagged NEMO domain constructs together with GST‐tagged MARCH2. Whole‐cell lysates were subjected to a GST‐PD assay, followed by immunoblotting with an anti‐HA antibody.GHEK293T cells transfected with His‐tagged ubiquitin and HA‐tagged NEMO‐WT or NEMO lacking the CC1 domain together with Strep‐tagged empty vector or MARCH2 were treated with MG132 (10 μM) for 6 h before harvest. Lysates were subjected to immunoprecipitation with an anti‐HA antibody, followed by immunoblotting with an anti‐His antibody. WCL were subjected to immunoblotting with anti‐HA, anti‐Strep, or anti‐β‐actin antibody.H, I(H) Schematic representation of the domain constructs of NEMO. (I) HEK293T cells transfected with GST‐tagged NEMO mutants together with Strep‐tagged empty vector or MARCH2 were subjected to immunoblotting with anti‐GST, anti‐Strep, or anti‐β‐actin antibody.JHEK293T cells transfected with HA‐tagged ubiquitin and GST‐tagged NEMO mutants together with Strep‐tagged empty vector or MARCH2 were subjected to pull‐down with GST beads, followed by immunoblotting with anti‐Lys48 (K48) linkage‐specific polyubiquitin (K48‐ub), anti‐Lys63 (K63) linkage‐specific polyubiquitin (K63‐ub), or anti‐Strep antibody. Whole‐cell lysates were determined by immunoblotting with the indicated antibodies.Data information: Data are representative of three independent experiments, each with similar results. Source data are available online for this figure.

**Figure EV5:**
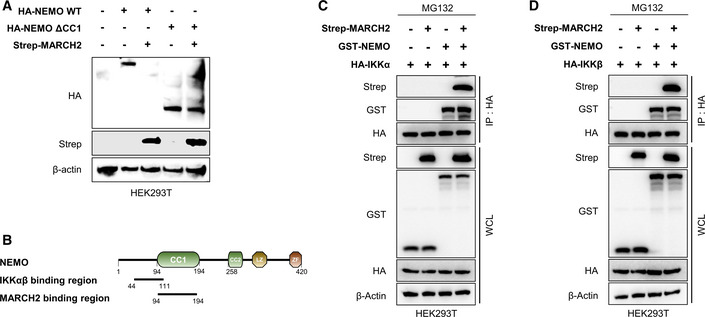
MARCH2 leads to NEMO degradation, but not its dissociation from IKK complex via interaction with CC1 domain AHEK293T cells were transfected with HA‐tagged NEMO‐WT or NEMO‐∆CC1 in the absence or presence of Strep‐tagged MARCH2. Whole‐cell lysates were subjected to immunoblotting with anti‐HA, anti‐Strep, or anti‐β‐actin antibody.BSchematic presentation of NEMO domain, interacting with IKK complex (IKKα, IKKβ) or MARCH2.C, DHEK293T cells transfected with HA‐tagged (C) IKKα or (D) IKKβ together with indicated plasmids were treated with MG132 (10 μM) for 6 h before harvest. Whole‐cell lysates were subjected to immunoprecipitation with an anti‐HA antibody, followed by immunoblotting with an anti‐HA, anti‐Strep, or anti‐GST antibody. Whole‐cell lysate was subjected to above antibody together with anti‐β‐actin.Data information: Data are representative of at least two independent experiments, each with similar results. Source data are available online for this figure.

Thus far, the results indicate that MARCH2 interacts with NEMO and mediates K48‐linked polyubiquitination. To identify the domain within MARCH2 that interacts with NEMO, we constructed Strep‐tagged RING and transmembrane (TM) MARCH2 expression plasmids (Fig 6A). A Strep pull‐down assay of each construct plus NEMO showed that NEMO bound strongly to the TM domain, but not to the RING domain, of MARCH2 (Fig 6B). Next, to investigate the specific domain of NEMO responsible for interaction with MARCH2, we generated four NEMO deletion mutants and used a GST pull‐down assay with either the TM domain or full‐length MARCH2 (Fig 6C). CC1 domain‐containing constructs interacted with the TM domain of MARCH2, as did full‐length MARCH2 (Fig 6D and Appendix Fig S9A). To verify this, we generated a CC1‐deleted NEMO construct and examined its interactions (Fig 6E). As expected, NEMO lacking the CC1 domain did not bind to MARCH2 (Fig 6F), resulting in neither ubiquitinated nor degraded MARCH2 (Figs 6G and EV5A); also, NEMO lacking the CC1 domain did not localize with MARCH2 (Appendix Fig S9B).

Next, to check the relationship between MARCH2 and the IKK complex, we assessed the interaction between MARCH2 and NEMO together with IKKα or IKKβ. The immunoprecipitation results showed no change in the interaction between NEMO and IKKα or IKKβ, regardless of MARCH2 expression, suggesting that MARCH2 most likely does not trigger dissociation of NEMO from IKKs, even though the binding region between NEMO and IKKs overlaps the CC1 domain by a few residues (Fig EV5B–D). These findings indicate that interaction between the CC1 domain of NEMO and the TM domain of MARCH2 is essential for regulation of innate immune responses.

**Figure 7:**
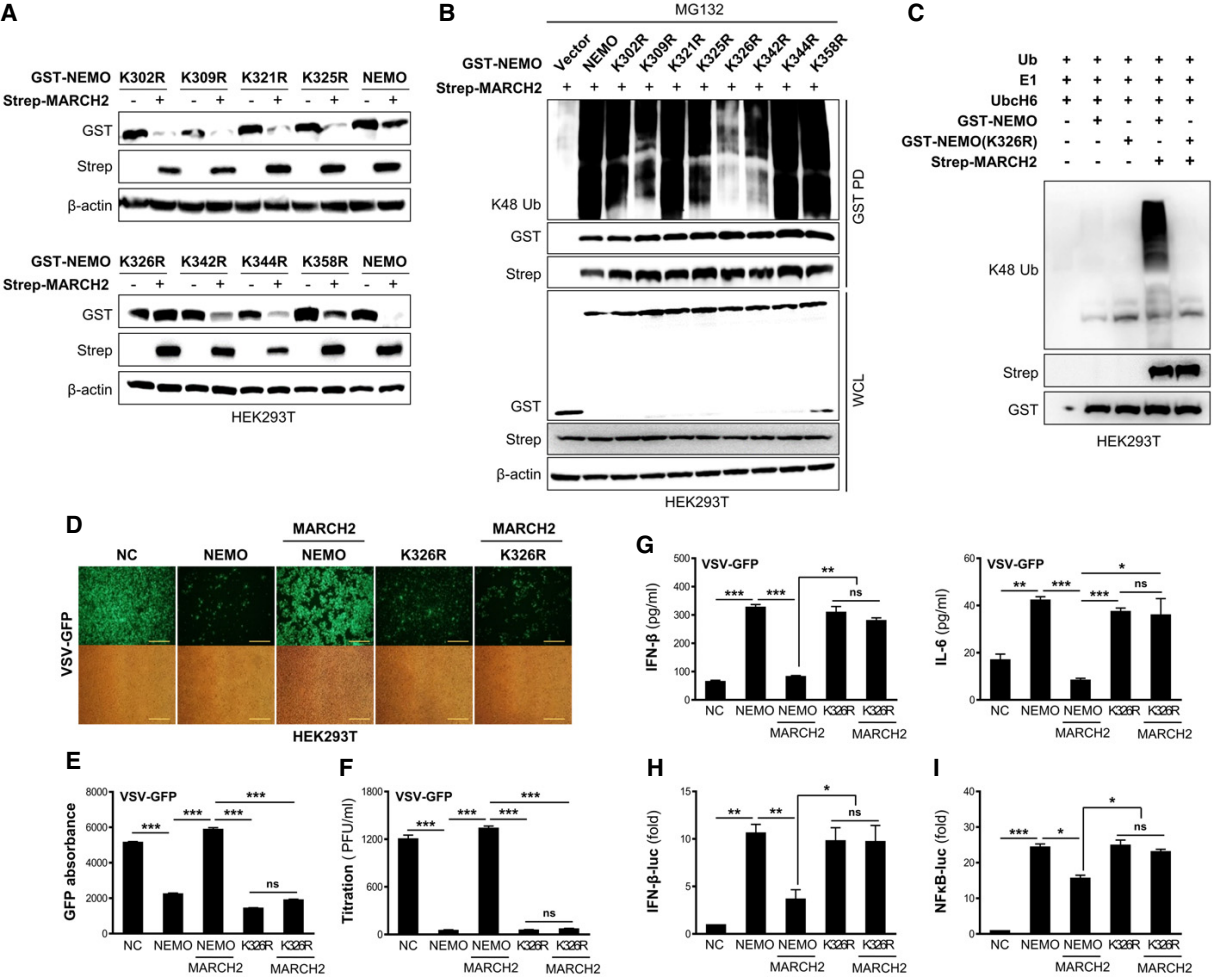
MARCH2 targets Lys 326 of NEMO for K48‐linked polyubiquitination AHEK293T cells were transfected with GST‐tagged NEMO‐WT and its point mutants with or without Strep‐tagged MARCH2. Whole‐cell lysates were subjected to immunoblotting with the indicated antibodies.BHEK293T cells were transfected with GST‐tagged NEMO‐WT and its point mutants together with Strep‐tagged MARCH2 in the presence of MG132. Whole‐cell lysates were subjected to a GST‐PD assay, followed by immunoblotting with an anti‐K48 linkage‐specific polyubiquitin antibody.C
*In vitro* ubiquitination assay for NEMO‐K326R. HEK293T cells were transfected with GST‐tagged NEMO, GST‐tagged NEMO‐K326R, or Strep‐tagged MARCH2 individually. Immunoprecipitates were incubated with a reaction mixture containing ubiquitin, E1, and UbcH6 (E2), followed by immunoblotting with an anti‐K48 linkage‐specific polyubiquitin antibody.D–GHEK293T cells transfected with GST‐tagged empty vector, NEMO‐WT, or NEMO‐K326R, with or without Strep‐tagged MARCH2 were infected with VSV‐GFP. (D‐F) Viral replication was determined by fluorescence microscopy (D), fluorescence absorbance (E), and a plaque assay (F). (G) IFN‐β and IL‐6 in the cell supernatants were measured in an ELISA.H, IIFN‐β (H) and NF‐κB (I) luciferase reporter assay.Data information: ns, not significant. *P <* *0.05, **P <* *0.01, ***P <* *0.001 (two‐tailed Student's *t‐*test). Data are representative of at least two independent experiments, each with similar results, and expressed as the mean ± SD of three biological replicates. Source data are available online for this figure.

Next, we generated four deletion mutants of NEMO to identify the region within NEMO that is ubiquitinated by MARCH2 (Fig 6H). We then examined degradation and ubiquitination of the NEMO constructs. NEMO harboring the LZ domain with or without the ZF domain was degraded (Fig 6I) and exhibited K48‐linked ubiquitination (Fig 6J), indicating that amino acids 296–347 contained the ubiquitin‐binding site(s). To identify the specific Lys residue within NEMO, we generated mutants in which each Lys was substituted with Arg (K302R, K309R, K321R, R325R, K326R, K342R, K344R, and K358R). Then, we assessed MARCH2‐mediated degradation of each mutant and compared it with that of the wild‐type. As shown in Fig 7A and B, substitution of Lys326 conferred complete resistance to degradation by MARCH2 and did not show K48‐linked polyubiquitination, whereas all other substitutions were intact. Moreover, an *in vitro* ubiquitination assay was performed in the presence of MARCH2, E1 and E2 enzymes, and ubiquitin, plus NEMO or NEMO‐K326R. Consistent with the results presented in Fig 7B, K48‐linked polyubiquitination was observed only on NEMO but not on NEMO‐K326R (Fig 7C). These findings demonstrate that Lys326 within NEMO is a specific site for K48‐linked polyubiquitination by MARCH2. Furthermore, we confirmed that the effect of NEMO K326R is the same as that of NEMO in terms of functionality in response to viral infection. We found that virus replication in the presence of both NEMO and NEMO K326R was lower than that in the control (Fig 7D–F). However, the antiviral effects of NEMO K326R were not affected by expression of MARCH2, while those of NEMO were attenuated (Fig 7D–F). In addition, NEMO K326R induced higher expression of IFN‐β and IL‐6 upon viral infection and was not affected by expression of MARCH2 (Fig 7G). Finally, these results were confirmed in a reporter gene assay (Fig 7H and I). Both NEMO and NEMO K326R induced IFN‐β and NF‐κB luciferase activity, but NEMO K326R was not affected by co‐expression of MARCH2. Collectively, these data indicate that Lys326 is critical for MARCH2‐mediated K48‐linked polyubiquitination and degradation of NEMO.

## Discussion

The MARCH E3 ligase family comprises proteins related to viral immune evasion (Früh *et al*, 2002). Early studies show that viral RING‐CH E3 ligases such as K3 and K5 of Kaposi's sarcoma‐associated herpesvirus negatively regulate diverse immunoreceptors to facilitate immune escape; indeed, 11 mammalian homologues, known as MARCH proteins, were identified subsequently (Früh *et al*, 2002). Most MARCH proteins share a similar structure, which includes an N‐terminal RING‐CH finger motif and two or more C‐terminal transmembrane domains that target various subcellular membranes such as the plasma membrane, the endosomal membrane, the ER, and the Golgi (Nathan & Lehner, 2009). MARCH proteins ubiquitinate and downregulate critical transmembrane proteins (Zhang *et al*, 2019). MARCH1 and MARCH8 downregulate expression of CD86 and MHC‐II (Goto *et al*, 2003; Ohmura‐Hoshino *et al*, 2006; De Gassart *et al*, 2008; Baravalle *et al*, 2011; Corcoran *et al*, 2011). In addition, MARCH8 triggers degradation of IL1RAP, CD98, TRAIL receptor 1, and the transferrin receptor (Eyster *et al*, 2011; Chen *et al*, 2012; Fujita *et al*, 2013; van de Kooij *et al*, 2013), while MARCH4 and MARCH9 reduce expression of Mult1 and CD166 (Bartee *et al*, 2006; Nice *et al*, 2010). Also, MARCH4 and MARCH8 downregulate CD81 and CD44 (Bartee *et al*, 2010). With the exception of cellular transmembrane proteins, MARCH proteins control diverse cellular proteins, such as MARCH1, to reduce cell surface expression of TRAIL‐R1 and regulate insulin signaling via interacting with INSR (van de Kooij *et al*, 2013; Nagarajan *et al*, 2016) while MARCH5 interacts with TANK and MAVS to regulate immune response to RNA virus infection (Shi *et al*, 2011; Yoo *et al*, 2015). MARCH2 may be the first member of the ubiquitin ligase family found to be associated with viral immune evasion proteins (Boutell *et al*, 2002). However, the specific immune modulatory function of MARCH2 upon viral or bacterial infection remained unknown until now.

Here, we show that MARCH2 is a potent negative regulator of innate immune responses against infection by viruses or bacteria. First, we generated MARCH2^−/−^ mice and showed that they exhibited low levels of viral replication and bacterial growth, along with elevated expression of pro‐inflammatory cytokines and type I interferons in response to viral or bacterial infection. In addition, challenge of MARCH2^−/−^ mice with LPS resulted in inflammatory responses that were more lethal than those in wild‐type mice. Second, knockout or knockdown of endogenous MARCH2 in immune cells and HEK293T cells reduced viral replication or bacterial growth, which correlated well with strong type I IFN and inflammatory responses. Consistent with these results, we found that overexpression of MARCH2 by immune cells and HEK293T cells suppressed host antiviral or antibacterial responses. Third, MARCH2 interacted directly with NEMO, but not with IKKα and IKKβ, at the late stage of infection. Fourth, the E3 ligase enzymatic activity of MARCH2 is critical for K48‐linked polyubiquitination and proteasomal degradation of NEMO. Finally, NEMO undergoes MARCH2‐mediated K48‐linked polyubiquitination on lysine 326. Taken together, our findings suggest that MARCH2 plays an important role in regulating NEMO and inhibiting excessive antiviral or antibacterial innate immune responses.

To date, there is limited *in vivo* evidence related to the physiological functions of MARCH family proteins, including MARCH2, although a few *in vitro* studies have been published (Xia *et al*, 2017; Xia, 2019). Recently, Lee *et al* (2018) reported that MARCH2 targets Xenopus Dsh for ubiquitin‐mediated degradation and antagonization of Wnt signaling, thereby negatively regulating head formation. In this study, MARCH2^−/−^ mice were generated using the CRISPR‐Cas9 system and verified genetic knockout of the MARCH2 gene without any external abnormalities (Fig EV1). We found that MARCH2^−/−^ mice were more resistant to infection by viruses or bacteria and more sensitive to LPS‐induced sepsis than wild‐type mice. These results strongly support a role for MARCH2 as a negative regulator of virus‐ or bacteria‐mediated innate immune responses.

We also investigated the antiviral and antibacterial roles of MARCH2 in BMDMs, PBMCs, and PMs isolated from MARCH2^−/−^ mice, and in MARCH2 knockdown Raw2647 cells. Consistent with the results in mice, we found that knockout or knockdown of MARCH2 in immune cells reduced viral replication or bacterial growth, and led to higher production of IL‐6, IL‐12, and IFN‐β production upon virus or bacterial infection. To examine the general function of MARCH2, we established a MARCH2 knockout HEK293T epithelial cell line using the CRISPR‐Cas9 system and confirmed similar results after virus infection. However, reconstitution of MARCH2 restored function by rescuing production of cytokines. Additionally, overexpression of MARCH2 by Raw264.7 cells and HEK293T cells increased viral replication and bacterial growth.

Increasing evidence suggests that excessive production of IFNs and pro‐inflammatory cytokines leads to development of autoimmune diseases, some cancers, and chronic inflammatory diseases (Ben‐Neriah & Karin, 2011; Ruland, 2011). Therefore, diverse molecular mechanisms tightly regulate the NF‐κB and type I IFN signaling pathways at different levels. A number of proteins have been reported as negative regulators that maintain immune homeostasis (Ruland, 2011; Anand *et al*, 2012; Long *et al*, 2014; James *et al*, 2015; Lu *et al*, 2017), such as MARCH2. Although the details remain elusive, these regulators form complicated networks and interact closely to compensate or replace each other.

NEMO/IKKγ is a crucial adaptor molecule that activates IKKα and IKKβ, and plays a key role in regulating the NF‐κB and type I IFN signaling pathways (Fujita *et al*, 2014). Therefore, NEMO/IKKγ must be tightly controlled to maintain immune homeostasis. In particular, ubiquitination of NEMO/IKKγ plays an essential role in modulating its functional activity. As positive regulators, Bcl10 and MALT1 induce K63‐linked ubiquitination of NEMO/IKKγ (Sun *et al*, 2004; Zhou *et al*, 2004). In addition, linear ubiquitination of NEMO/IKKγ is triggered by the LUBAC complex (Tokunaga *et al*, 2009) in a ligand‐dependent manner. Also, TRIM23 induces K27‐linked ubiquitination of NEMO/IKKγ during antiviral responses (Arimoto *et al*, 2010). By contrast, NEMO/IKKγ is negatively regulated by specific deubiquitinases (DUBs) such as CYLD, TNFAIP3/A20, and OTU deubiquitinases (OTULIN) (Kovalenko *et al*, 2003; Wen *et al*, 2004; Lork *et al*, 2017). In addition, a recent report shows that K48‐linked polyubiquitination of NEMO/IKKγ by the E3 ubiquitin ligase TRIM29 in alveolar macrophages leads to proteasomal degradation of NEMO/IKKγ and interferes with activation of the IKK complex. However, Xing *et al* (2016) showed that TRIM29 is a selective regulator for downregulation of NEMO/IKKγ in alveolar macrophages via ubiquitination and degradation.

In this study, we identified MARCH2 as a novel negative regulator of NEMO/IKKγ via a proteasomal degradation mechanism previously unreported for the NF‐κB signaling and type I IFN signaling cascades. The TM domain of MARCH2 interacted directly with the CC1 domain of NEMO, and, importantly, MARCH2 did not bind to NEMO/IKKγ under normal conditions. However, based on the time course‐binding studies, MARCH2 bound strongly to NEMO at the late stage after viral or bacterial infection, and induced ubiquitination and degradation of NEMO/IKKγ in both immune cells and epithelial cells. These results strongly suggest that MARCH2 negatively regulates excessive production of IFNs and inflammatory responses against viruses or bacteria via degradation of NEMO/IKKγ to maintain immune homeostasis. However, the upstream signaling molecule(s) that regulates MARCH2 remains to be identified. It is possible that unknown transcription factors are engaged in MARCH2 expression; also, specific post‐translational modifications of MARCH2 by upstream molecules may initiate or terminate MARCH2 activation, which ultimately regulates degradation of NEMO/IKKγ. However, this plausible hypothesis awaits further study.

Additionally, previous reports identified the specific lysine residues in NEMO that are ubiquitinated following stimulus; these include Bcl10‐mediated K63‐linked polyubiquitination at Lys399 (Zhou *et al*, 2004), RIP2 or TRAF6‐mediated K63‐linked polyubiquitination at Lys285 and Lys399 (Abbott *et al*, 2004; Sebban‐Benin *et al*, 2007), LUBAC‐mediated linear ubiquitination at Lys285 and Lys309 (Tokunaga *et al*, 2009), TRIM23‐mediated K27‐linked polyubiquitination at Lys183 (Arimoto *et al*, 2010), and IpaH9.8 E3 ligase of *Shigella flexneri*‐mediated K27‐linked polyubiquitination at Lys309 and Lys321 (Ashida *et al*, 2010). In this study, we identified a specific amino acid, Lys326, in NEMO/IKKγ as being crucial for MARCH2‐mediated K48‐linked ubiquitination. Furthermore, given the functional importance of NEMO/IKKγ, Lys326 (and its degradation by MARCH2) is thought to be linked to inflammatory disorders or other diseases.

In summary, we identified the E3 ubiquitin ligase MARCH2 as a negative regulator of NEMO/IKKγ after infection by viruses or bacteria. MARCH2 targets NEMO/IKKγ and induces its Lys326‐mediated polyubiquitination and proteasomal degradation, thereby preventing excessive NF‐kB and type I interferon signaling. Consequently, MARCH2 plays a physiologically essential role in maintaining immune homeostasis. These findings have important implications for the understanding of innate immune responses as well as for providing useful therapeutic strategies for pathogen‐associated or chronic inflammatory diseases.

## Materials and Methods

### Mice and *in vivo* experiments

All animal experiments were approved by the Institutional Animal Use and Care Committee of Chungnam National University (Reference number CNU‐00813, CNU‐00921, and CNU‐00927) and were performed in biosafety level BSL‐2 laboratory facilities with the Guide for the Care and Use of Laboratory Animals (published by the US National Institutes of Health). For the viral infection experiment, sex‐matched mice (6‐ to 8‐week‐old) were intravenously infected with VSV‐GFP (2 × 10^8^ PFU/mouse) or injected with poly(I:C) (200 μg/mouse). Serum samples were collected at 12 hpi (VSV‐GFP) or 2 hpi (poly(I:C)) to examine level of cytokines by ELISA. For the bacterial infection experiment, mice were intraperitoneally infected with *L. monocytogenes* (1 × 10^6^ CFU/mouse). Spleens and livers were aseptically collected to determine the bacterial loads or secretion level of cytokines or chemokines. Sera were collected to measure secretion level of cytokines or chemokines. For LPS challenge experiment, mice were intraperitoneally challenged with LPS (24 mg/kg). Sera, spleens, and livers were used for measuring secretion or mRNA expression level of cytokines or chemokine, or H&E staining (only spleens).

### Generation of MARCH2 knockout mice

Target site at the second exon of the MARCH2 gene (Fig EV1) was validated and synthesized from the ToolGen South Korea. (gRNA‐CCTATGTGACTGTTCGAGCAGCC). Female mice (C57BL/6J, 4–6 weeks old) were used as embryo donors for superovulation. After mating, resultant fertilized embryos were collected from the oviducts and cultured in KSOM medium as previously described (Yang *et al*, 2014; An *et al*, 2015). Microinjection was performed using an Olympus IX71 Inverted Microscope with the Narishigi microinjection system. gRNA (50 ng/μl) and Cas9 mRNA (100 ng/μl) were mixed and injected into the cytoplasm of the zygotes with visible pronuclei in M2 medium as previously described (Wang *et al*, 2013). The injected embryos were cultured overnight in KSOM medium at 37°C with 5% CO_2_. Embryos at the two‐cell stage were transferred into oviducts of pseudopregnant ICR females. Tail samples from 3‐week‐old live pups were collected for extraction of genomic DNA and sequenced. Presumptive heterozygous MARCH2^+/−^ were mated. To screen MARCH2 KO mice, T7E1 assays were performed as previously described using genomic DNA samples from tail biopsies (Cho *et al*, 2013). Briefly, the genomic region included RNA‐guided endonucleases target site was PCR‐amplified with nested PCR method, melted, and reannealed to form heteroduplex DNA, which was treated with T7 endonuclease 1 (New England Biolabs) and then analyzed by agarose gel electrophoresis. Once heterogeneous mice were excluded, additional T7E1 assay was conducted by mixing equal amount of wild‐type PCR products and WT and KO mice were separated by agarose gel electrophoresis. The primers used for nested PCR are listed in Appendix Table S3.

### Cell culture and transfection

HEK293T (ATCC‐11268), Raw264.7 (ATCC TIB‐71), HeLa (ATCC CCL‐2), Vero (ATCC CCL‐81), and A549 (ATCC CCL‐185) cells were cultured in DMEM (Invitrogen) supplemented with 10% fetal bovine serum (FBS) (Gibco) and 1% antibiotic/antimycotic (Thermo Fisher Scientific) in a humidified 5% CO_2_ incubator at 37°C. To establish Raw264.7 cells stably expressing pIRES, pIRES‐MARCH2‐Flag, or pIRES‐MARCH2^CCH^‐Flag, cells were transfected with respective plasmids using Lipofectamine 2000 (Invitrogen) and selected in DMEM supplemented with 2 μg/ml of puromycin (Thermo Fisher Scientific) at least for 2 weeks. HEK293T cells were transiently transfected with the indicated plasmids or poly(dA:dT), 5′‐triphosphate double‐stranded RNA using polyethylenimine (PEI; Polyscience Inc.), or Lipofectamine 2000, respectively.

### Generation of MARCH2 knockout cells

A MARCH2 KO HEK293T cell line was established by CRISPR/Cas9‐mediated genome editing. The target sequences for CRISPR interference were designed by ToolGen Inc. (Seoul, South Korea). HEK293T cells were transfected with 500 ng dRGEN‐Human MARCH2‐U6‐SG plasmid, CAS9, and 1,000 ng surrogate reporter (ToolGen) using Lipofectamine 2000. MARCH2 KO cells were selected by puromycin.

### Primary cell isolation

Bone marrow‐derived macrophage (BMDM) isolation was performed as described previously (Kim *et al*, 2018). In brief, the femurs and tibias were isolated from MARCH2^+/+^ or MARCH2^−/−^ mice aseptically and flushed with medium. After RBCs were removed by ACK lysis buffer (Gibco), cells were incubated with DMEM containing 10% FBS, antibiotics, and mGM‐CSF (10 ng/ml) for 6 days to be differentiated to BMDMs. PBMCs were isolated from peripheral blood using Ficoll‐Paque PREMIUM 1.703 (GE healthcare) with SepMate (STEMCELL) in accordance with manufacturer's protocol.

Peritoneal macrophages (PMs) were aseptically obtained by flushing peritoneal cavity of MARCH2^+/+^ or MARCH2^−/−^ mice with HBSS w/o phenol red and cultured with DMEM containing 10% FBS and antibiotics (Xiang *et al*, 2015).

### Plasmids

To generate MARCH2 different construct, WT MARCH2 was amplified from template DNA using PCR and cloned into pIRES‐Flag, pEXPR‐Strep, or pEBG vectors. Strep‐tagged MARCH2‐RING domain and transmembrane domain were subcloned into pEXPR vector. To generate the catalytically inactivated RING‐CH domain in MARCH2, three amino acids that are important for enzymatic activity were mutated, MARCH2^CCH^ (cysteines 64 and 67 to serines and histidine 90 to glutamine), and cloned into Strep‐ and Flag‐tagged vectors. To generate NEMO different construct, WT NEMO was amplified from template DNA using PCR and cloned into pEBG and pCMV‐HA vectors. Shortly, PCR‐amplified abbreviated GST‐tagged NEMO mutants carrying each domain were cloned into pEBG vector and HA‐tagged NEMO deletion mutant (NEMO‐∆CC1) was cloned into pCMV vector. pRK5‐HA‐ubiquitin‐KO, K6, K11, K27, K29, K33, K48, K63, and WT were gifted from Ted Dawson (Addgene plasmid numbers: #17603, #22900, #22901, #22902, #22903, #17607, #17605, #17606, #17608). pCI‐His‐hUbi was a gift from Astar Winoto (Addgene plasmid number: #31815). (Several lysine (K) amino acids having probability to accepting ubiquitin proteins were mutated to arginine (R) as K302R, K309R, K321R, K325R, K326R, K342R, K344R, and K358R). All point mutations were generated by PCR using a QuikChange Site‐Directed Mutagenesis Kit (Stratagene). The PCR primers used for site‐directed mutagenesis are listed in Appendix Table S4.

### Reagents and antibodies

Poly(I:C) and Poly(dA:dT) (InvivoGen), puromycin (Gibco), Lipofectamine 2000 and Lipofectamine RNAiMAX (Invitrogen), Protein A/G PLUS‐Agarose (Santa Cruz Biotechnology), MG132 (Sigma‐Aldrich), LPS (Sigma‐Aldrich), zymosan (InvivoGen), β‐glucan (Curdlan AL, InvivoGen), imiquimod (Invitrogen), CpG ODN2395 (Invitrogen), Sepharose 6B (GE Healthcare Life Science), glutathione‐conjugated Sepharose 4B (GST) beads (GE Healthcare Life Science), Strep‐Tactin Sepharose (1201‐0590, IBA), NH_4_Cl (Sigma‐Aldrich), Z‐VAD (Santa Cruz Biotechnology), chloroquine (Sigma‐Aldrich), and LysoTracker Red DND‐99 (Thermo Fisher Scientific) were obtained commercially. All the antibodies used for the immunoblot analysis and immunofluorescence experiments are listed in Appendix Tables S1 and S2.

### *In vivo* and *in vitro* ubiquitination assay

For the *in vivo* ubiquitination assay, HEK293T cells were treated with 10 μM of MG 132 for 8–12 h before harvest. Whole cells were lysed with RIPA buffer (50 mM Tris–HCl (pH 7.4), 150 mM NaCl, 1% NP‐40, 0.1% SDS, 0.1% sodium deoxycholate, 5 mM EDTA) containing protease inhibitor (Roche). The equal amount of protein lysates was subjected to pull‐down with GST beads or immunoprecipitation with anti‐NEMO antibody overnight at 4°C, followed by incubation with Protein A/G PLUS‐Agarose (only for IP) for 3 h at 4°C. The immune complex was washed extensively for three times with RIPA buffer and boiled with sample buffer for 10 min. For *in vitro* ubiquitination assay, immunoprecipitated GST‐NEMO, Strep‐MARCH2, and Strep‐MARCH2^CCH^ were prepared from lysates of HEK293T cells transfected with individual plasmids. Immunoprecipitates were incubated with 100 ng of E1 (Boston Biochem), 400 ng of E2 (UbcH6, Sigma‐Aldrich), and 2 μg of ubiquitin (Boston Biochem) in a 1× ATP regeneration solution (Enzo, BML‐EW9810) for 2 h at 30°C. The reaction was terminated by adding 4× sample buffer and boiling for 10 min. Ubiquitination was determined by immunoblotting with anti‐K48 or K63 linkage‐specific polyubiquitin antibodies.

### Quantitative real‐time PCR

Total RNA was isolated from cells or 1 mg of mice tissues using RNeasy Mini Kit (Qiagen). Complementary DNA (cDNA) synthesis was performed using reverse transcriptase (TOYOBO), followed by qPCR using gene‐specific primer sets (Bioneer) and QuantiTect SYBR Green PCR Kit (TOYOBO) according to the manufacturer's protocol on a Rotor‐Gene Q (Qiagen). The sequences of the primers used in qPCR are listed in Appendix Table S5.

### Luciferase reporter assay

HEK293T cells were transfected with a luciferase reporter plasmid, a thymidine kinase promoter‐Renilla (TK‐Renilla) luciferase reporter plasmid, and each of the individual plasmids using PEI. At 24 h post‐transfection, cells were washed with PBS and lysed with 1× passive lysis buffer (Promega). Luciferase activity was measured using Dual‐Luciferase Reporter Assay System (Promega; E1980) following manufacturer's instruction. Luciferase activity in cells expressing only reporter and Renilla plasmids was measured as a control. Data are expressed in accordance with relative firefly luciferase activity normalized against Renilla luciferase activity.

### Small interfering RNA transfection

Cells were transfected with small interfering RNA (siRNA) oligonucleotides (list in Appendix Table S6) using Lipofectamine RNAiMAX (Invitrogen) according to the manufacturer's protocol. A non‐specific siRNA was used as a control. Cells were incubated for 36–48 h prior to virus infection or treatment.

### Virus replication assay

Cells were infected with virus in reduced serum (1% FBS)‐containing medium for 2 h. The culture supernatants containing uninfected virus were replaced with fresh complete medium. The cell supernatants and/or extracts obtained from freeze‐thawed cells were used for plaque assay. Homogenates of freeze‐thawed tissue extracts were used for plaque assay when titrating viruses present in mouse tissues. The images of cells expressing GFP were pictured by fluorescence microscope (200× magnification). The replication of GFP‐tagged virus was measured using fluorescence modulator (GloMax‐Multi Detection System, Promega) and determined by standard plaque assay.

### Quantification of bacterial growth

Spleens and livers collected aseptically from MARCH2^+/+^ and MARCH2^−/−^ were used for quantifying bacterial replication. The tissue homogenates were diluted 10‐fold in sterile PBS and plated on BHI (brain heart infusion) agar, followed by incubation at 37°C for 1 days. Colony‐forming unit (CFU) was utilized to ensure quantification of bacterial replication.

### ELISA

Mouse sera, tissue homogenates, or cell supernatants were used to examine level of cytokine secretion by ELISA. Commercial kits as followings were used according to manufacturer's protocols: mouse IFN‐β (PBL Interferon Source), mouse IL‐6 (BD Biosciences), mouse IL‐12 (BD Biosciences), mouse TNF‐α (BD Biosciences), mouse CCL5 (R&D Systems), mouse CXCL10 (R&D Systems), mouse IL‐1β (BD Biosciences), human IFN‐β (PBL interferon source), and human IL‐6 (BD Biosciences).

### Confocal microscopy

HeLa or HEK293T cells were seeded into eight‐well chamber slide (ibidi). LysoTracker Red was applied to live cell supernatant with 100 nM for 30 min at 37°C. For each experiment, cells were fixed with 4% paraformaldehyde at room temperature for 20 min. After washing with PBS, cells were permeabilized with 100% methanol at −20°C for 20 min then blocked with 2% BSA in PBS for 1 h at room temperature, followed by incubation with relevant primary antibodies at 4°C overnight. Next day, cells were washed with PBST for three times and incubated with appropriate secondary antibody. Then, cells were washed with PBST for three times and stained with DAPI (4′,6‐diamidino‐2‐phenylindole) at room temperature for 10 min. Images were acquired under Nikon laser scanning confocal microscope (C2plus) and analyzed using NIS‐Elements software.

### Membrane protein isolation, immunoblot analysis, and immunoprecipitation

Cytoplasm and membrane protein of HEK293T cells were isolated using the MinuteTM Plasma Membrane Protein Isolation Kit following manufacturer's protocol (Invent sm‐005). For immunoblot analysis, cells were lysed with radioimmunoprecipitation assay (RIPA) buffer (50 mM Tris–HCl, 150 mM NaCl, 0.5% sodium deoxycholate, 1% IGEPAL) containing protease inhibitor cocktail (Roche) and phosphatase inhibitor (Na_3_PO_4_ 1 mM). Whole‐cell lysates were subjected to SDS–PAGE, followed by immunoblotting with the specific antibodies. For pull‐down assay or immunoprecipitation (IP), cell lysates were pre‐cleared by incubation with Sepharose 6B (GE Healthcare) or Protein A/G PLUS‐Agarose (Santa Cruz Biotechnology) for 1 h at 4°C. The pre‐cleared cell lysates were incubated with glutathione (GSH) Sepharose 4B (GE Healthcare), Strep‐Tactin Superflow high‐capacity resin (IBA), or a primary antibody overnight at 4°C, followed by incubation with Protein A/G PLUS‐Agarose (only for IP) for 3 h at 4°C. The immunoprecipitates were washed with lysis buffer for three times before immunoblot analysis.

### Mass spectrometry

HEK293T cells transfected with Strep‐tagged empty vector or MARCH2 were harvested at 36 h post‐transfection. Lysates were subjected to pull‐down with Strep‐Tactin Superflow high‐capacity resin (IBA) overnight at 4°C. The resin was washed with lysis buffer for five times. The bound proteins were eluted with elution buffer (100 mM Tris–HCl, 150 mM NaCl, 1 mM EDTA, and 2.5 mM desthiobiotin). The eluted fractions were concentrated in Amicon Ultra‐0.5 (10K cutoff) centrifugal filter (Merck Millipore). The samples were then separated by 4–15% NuPAGE gels (Invitrogen), followed by silver staining (Wray *et al*, 1981). Protein bands present exclusively in the gel were subjected to mass spectrometry.

### Statistical analysis

Statistical analysis was performed using Prism 6 (GraphPad Software). Data are expressed as the mean ± SD of at least two independent experiments. Statistical significance was analyzed using unpaired Student's *t*‐test or the log‐rank test, as indicated in the legends.

## Data availability

This study includes no data deposited in external repositories.

## Author contributions

KC, T‐HK, HL, and J‐SP performed most of the experiments with help from J‐HK, WAGC, PE, YJC. C‐HL, C‐JK, and J‐UJ contributed to the discussion and provided critical materials. J‐SL designed the study and wrote the manuscript with help from all of authors; J‐SL supervised the study; All of authors helped with data analysis.

## Conflict of interest

The authors declare that they have no conflict of interest.

## Supporting information



Appendix

Expanded View Figures PDF

Source Data for Expanded View and Appendix

Review Proccess File

Source Data for Figure 1

Source Data for Figure 2

Source Data for Figure 3

Source Data for Figure 4

Source Data for Figure 5

Source Data for Figure 6

Source Data for Figure 7
