# Supplementary material for: Negative regulation of NEMO signaling by the ubiquitin E3 ligase MARCH2
Source: EMBO J. 2020 Sep 16;39(21):e105139. doi: 10.15252/embj.2020105139 (PMC7604578; doi:10.15252/embj.2020105139)
Supplement: Supplementary file 1 — Appendix [file EMBJ-39-e105139-s001.docx]

**Appendix Information**

**Negative regulation of the NEMO signaling by the ubiquitin E3 ligase MARCH2**

Kiramage Chathuranga^1,6^, Tae-Hwan Kim^1,2,6^, Hyuncheol Lee^1,3,6^, Jun-Seol Park^1,6^, Jae-Hoon Kim^4^, W. A. Gayan Chathuranga^1^, Pathum Ekanayaka^1^, Youn Jung Choi^5^, Chul-Ho Lee^4^, Chul-Joong Kim^1^, Jae U. Jung^5^ and Jong-Soo Lee^1*^

**List of contents**

**Appendix Figures**

**Appendix Figure S1:** MARCH2 knockout increases phosphorylation of signaling molecules and IFN, pro-inflammatory cytokine gene transcription upon pathogen infection in BMDMs.

**Appendix Figure S2:** MARCH2 knock down increases immune response upon pathogen infection in Raw264.7 cells.

**Appendix Figure S3:** MARCH2 stable expression in Raw264.7 reduces immune response upon pathogen infection.

**Appendix Figure S4:** Transient expression of MARCH2 in HEK293T cells reduces immune response upon pathogen infection.

**Appendix Figure S5:** MARCH2 endogenously interacts with NEMO upon pathogen infection or PAMP stimulation.

**Appendix Figure S6:** MARCH2 localizes on and out of ER membrane at late time of pathogen infection or PAMP stimulation.

**Appendix Figure S7:** MARCH2 localized on and out of lysosome at late time of pathogen infection or PAMP stimulation.

**Appendix Figure S8:** E3 ubiquitin ligase activity of MARCH2 is important for negative regulation of innate immune response.

**Appendix Figure S9:** NEMO CC1 domain is important for interaction with MARCH2.

**Appendix Tables**

**Appendix Table S1:** List of antibody used for immunoblotting experiments

**Appendix Table S2:** List of antibody used for immunofluorescence experiments

**Appendix Table S3:** List of primers used for MARCH2 mice genotyping

**Appendix Table S4:** List of primers used for site-directed mutagenesis

**Appendix Table S5:** List of primers used for real time PCR

**Appendix Table S6:** List of MARCH2 siRNA sequence

**
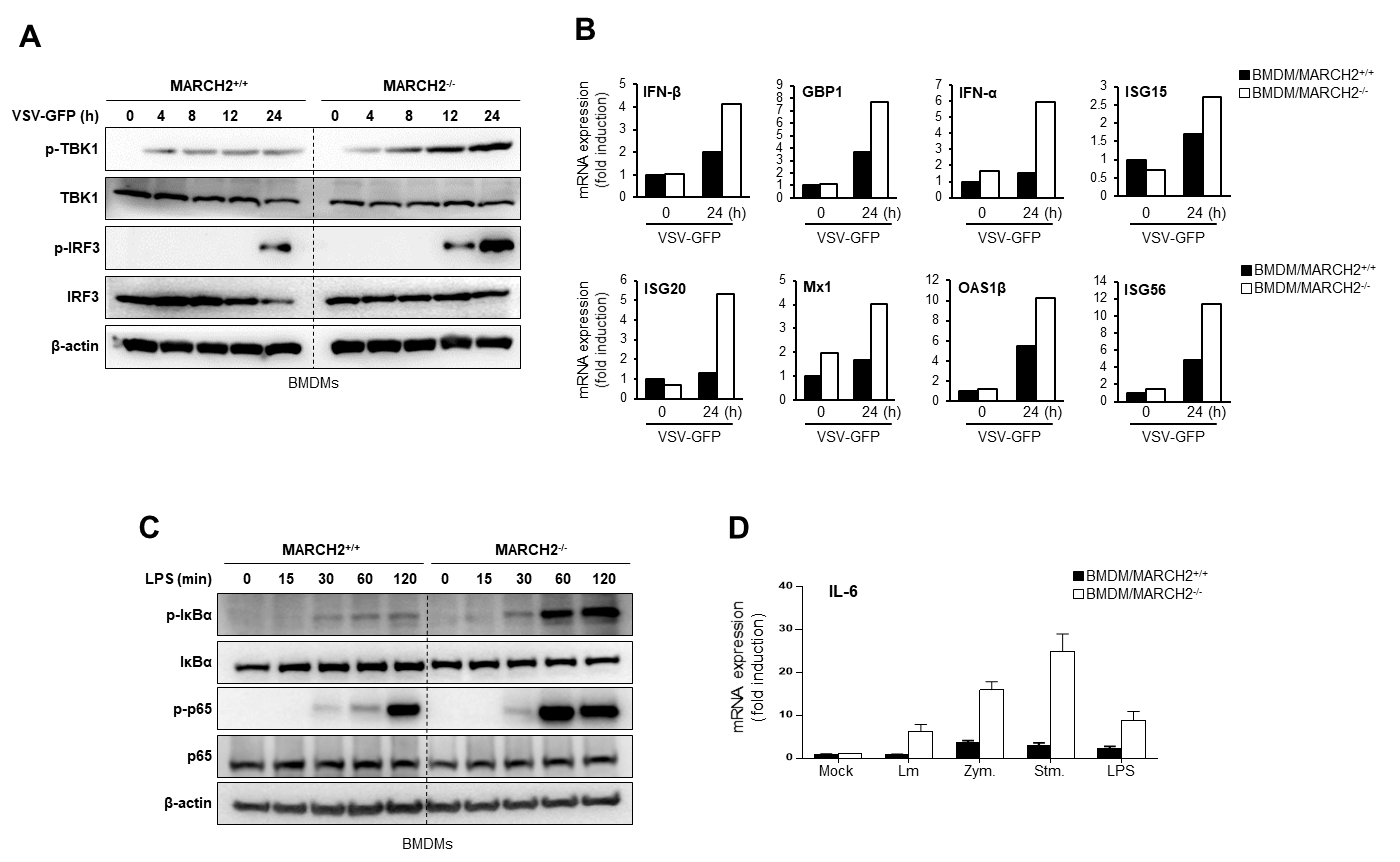
**

**Appendix Figure S1. MARCH2 knockout increases phosphorylation of signaling molecules and IFN, pro-inflammatory cytokine gene transcription upon pathogen infection in BMDMs.**

**A** BMDMs isolated from MARCH2^+/+^ and MARCH2^-/-^ mice were infected with VSV-GFP (MOI=5). Cells were harvested at indicated time points after virus infection, and total and phosphorylated TBK1, IRF3, were measured by immunoblotting in cell lysates. β-actin was used to confirm equal loading of protein.

**B** BMDMs isolated from MARCH2^+/+^ and MARCH2^-/-^ mice were infected with VSV-GFP (MOI=5). At 24 hpi total RNA were extracted. Expression of mRNA encoding IFN-β, IFN-α, OAS-1β, MX-1, ISG-15, ISG-20, p56, GBP1 was analyzed by qRT-PCR. GAPDH was used for normalization.

**C** BMDMs isolated from MARCH2^+/+^ and MARCH2^-/-^ mice were treated with LPS (50 ng/ml) in time-dependent manner. Cells were harvested after treatment, total and phosphorylated IҡB-α, and p65 were measured by immunoblotting. β-actin was used to confirm equal loading of protein.

**D** BMDMs isolated from MARCH2^+/+^ and MARCH2^-/-^ mice were infected with *L. monocytogenes* (MOI=1), *S. typhimurium* (MOI=1), or treated with zymosan (100 μg/ml), LPS (50 ng/ml). Expression level of IL-6 mRNA was determined by qRT-PCR. GAPDH was used for normalization.

Data information: Data are representative of at least three independent experiments, each with similar results, and expressed as the mean ± S.D. of two biological replicates.

Source data are available online for this figure.

**
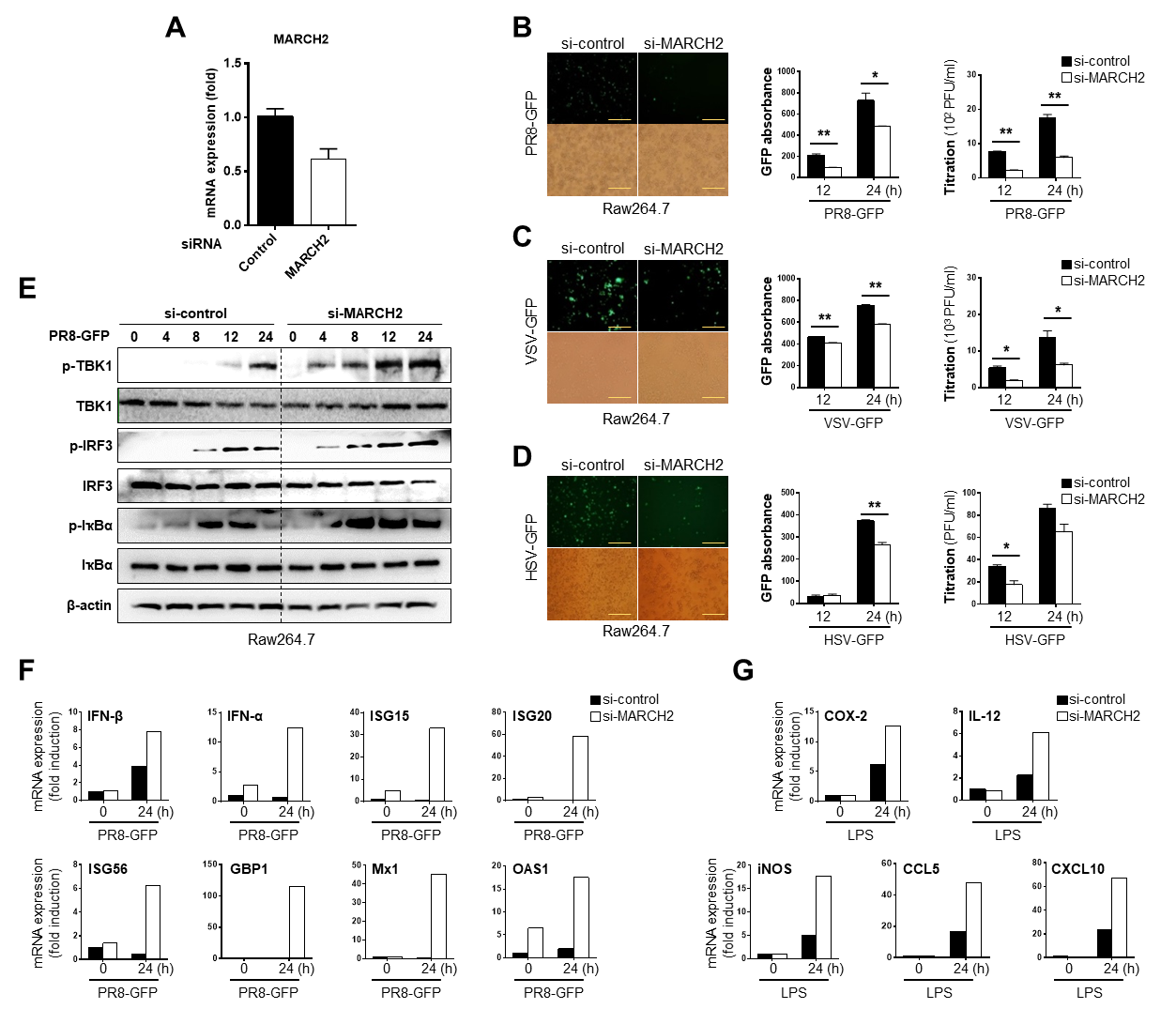
**

**Appendix Figure S2. MARCH2 knock down increases immune response upon pathogen infection in Raw264.7 cells.**

**A** qPCR analysis of MARCH2 depletion level in Raw264.7 cells transfected with control siRNA (si-Control) or MARCH2-specific siRNA (si-MARCH2).

**B-D** Raw264.7 cells were transfected with control siRNA (si-Control) or MARCH2-specific siRNA (si-MARCH2). Viral replication level was determined at 12 and 24 hpi by fluorescence microscopy, fluorescence absorbance and plaque assay, Scale bar, 50μm.

**E** Raw264.7 cells transfected with si-control or si-MARCH2 were infected with PR8-GFP (MOI=1). Cells were harvested at indicated time points after infection, total and phosphorylated TBK1, IRF3, IҡB-α were measured by immunoblotting in cell lysates. β-actin was used to confirm equal loading of protein.

**F, G** RAW 264.7 cells transfected with si-control or si-MARCH2 were infected with PR8-GFP (MOI=1) or LPS (50 ng/ml). Cells were harvested at indicated time points and mRNA transcription level of IFN and other antiviral gene (F) or pro-inflammatory cytokines (G) were evaluated.

Data information: **P* < 0.05, ***P* < 0.01 (two-tailed Student’s *t-*test). Data are representative of at least three independent experiments, each with similar results, and expressed as the mean ± S.D. of two biological replicates.

Source data are available online for this figure.

**
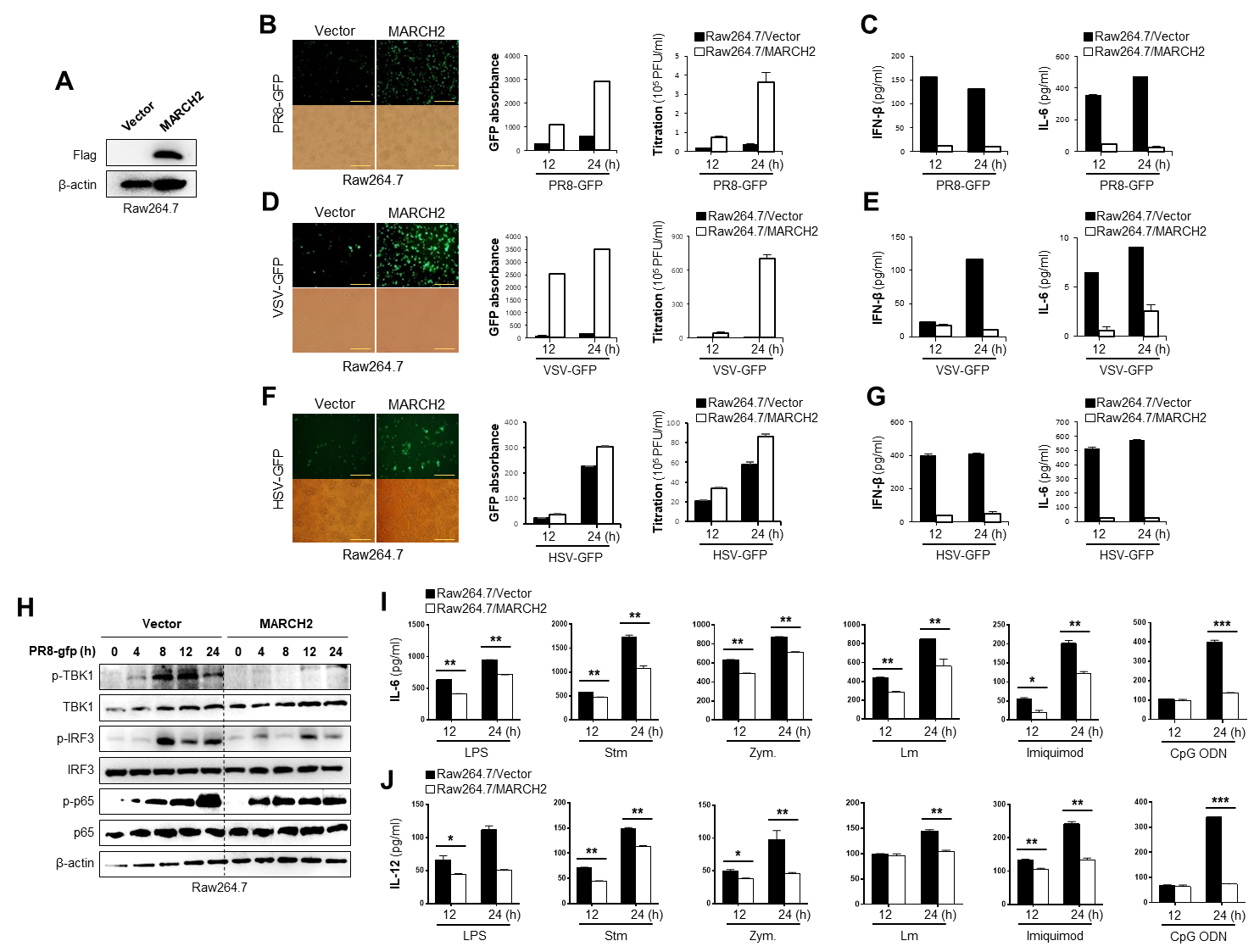
**

**Appendix Figure S3. MARCH2 stable expression in Raw264.7 reduces immune response upon pathogen infection.**

**A** Immunoblot analysis of MARCH2 expression in Raw264.7 cells stably expressing Flag-tagged empty vector or MARCH2.

**B-G** Raw264.7 cells harboring empty vector or MARCH2 were infected with PR8-GFP (B, C, MOI=1), VSV-GFP (D, E, MOI=0.5), HSV-GFP (F, G, MOI=2). Viral replication level was determined at 24 hpi by fluorescence microscopy, Fluorescence absorbance and plaque assay (B, D, F). Concentration of IFN-β and IL-6 secreted in supernatants were determined at 12 and 24 hpi by ELISA (C, E, G), Scale bar, 50μm.

**H** Raw264.7 harboring empty vector or MARCH2 were infected with PR8-GFP (MOI=1). Cells were harvested at indicated time points after infection, total and phosphorylated TBK1, IRF3, p65 were measured by immunoblotting in cell lysates. β-actin was used to confirm equal loading of protein.

**I, J** Raw264.7 harboring empty vector or MARCH2 were infected with *S. typhimurium* (MOI=1), *L. monocytogenes* (MOI=1) or treated with LPS (100 ng/ml), zymosan (50 μg/ml), imiquimod (5 mM), or CpG ODN (5 μg/ml). Concentration of IL-6 (I) or IL-12 (J) secreted in the cell supernatants was determined at 12 and 24 hpi by ELISA.

Data information: **P* < 0.05, ***P* < 0.01, ****P* < 0.001 (two-tailed Student’s *t-*test). Data are representative of at least two independent experiments, each with similar results, and expressed as the mean ± S.D. of two biological replicates**.**

Source data are available online for this figure.

**
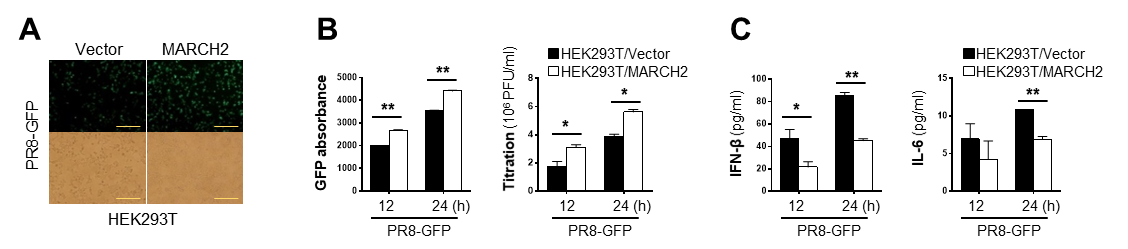
**

**Appendix Figure S4. Transient expression of MARCH2 in HEK293T cells reduces immune response upon pathogen infection.**

**A-C** HEK293T cells transiently transfected with Flag-tagged empty vector or MARCH2 were infected with PR8-GFP (MOI=0.5). Viral replication level was determined at 24 hpi by fluorescence microscopy (A), Fluorescence absorbance and plaque assay (B), concentration of IFN-β or IL-6 secreted in the cell supernatants was determined at 12 and 24 hpi by ELISA (C), Scale bar, 50μm.

Data information: **P* < 0.05, ***P* < 0.01 (two-tailed Student’s *t-*test). Data are representative of at least two independent experiments, each with similar results, and expressed as the mean ± S.D. of two biological replicates.

Source data are available online for this figure.


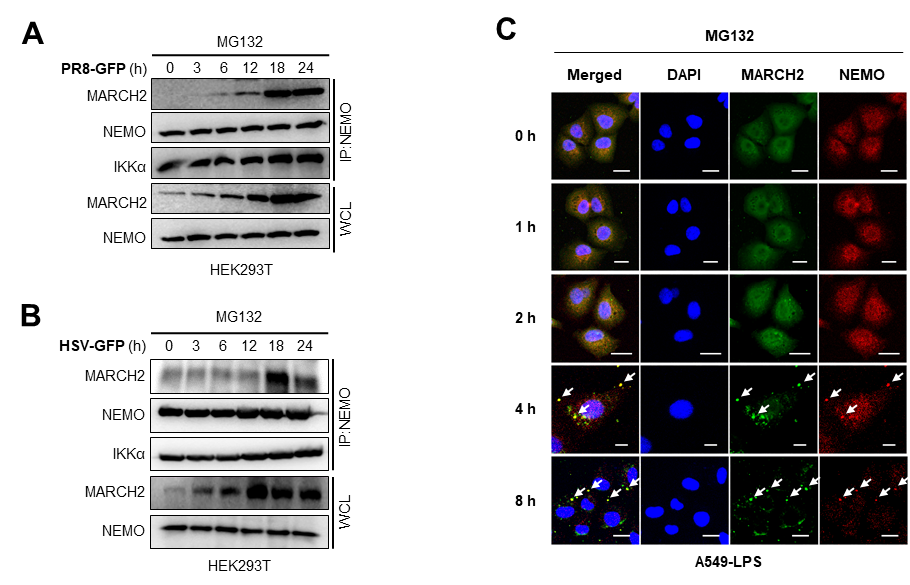


**Appendix Figure S5. MARCH2 endogenously interacts with NEMO upon pathogen infection or PAMP stimulation.**

**A-B** HEK293T cells were infected with PR8-GFP (A), HSV-GFP (B) in a time-dependent manner in the presence of MG132 (proteasome inhibitor, 10 μM). Whole cell lysates were subjected to immunoprecipitation with an anti-NEMO antibody, followed by immunoblotting with an anti-MARCH2 antibody.

**C** Co-localization between MARCH2 (green) and NEMO (red) in A549 cells upon LPS treatment (50 ng/ml) in the presence of MG132 (10 μM). Scale bar, 10μm. Arrow indicates the co-localized NEMO and MARCH2 protein.

Data information: Data are representative of at least two independent experiments, each with similar results.

Source data are available online for this figure (A, B).

**
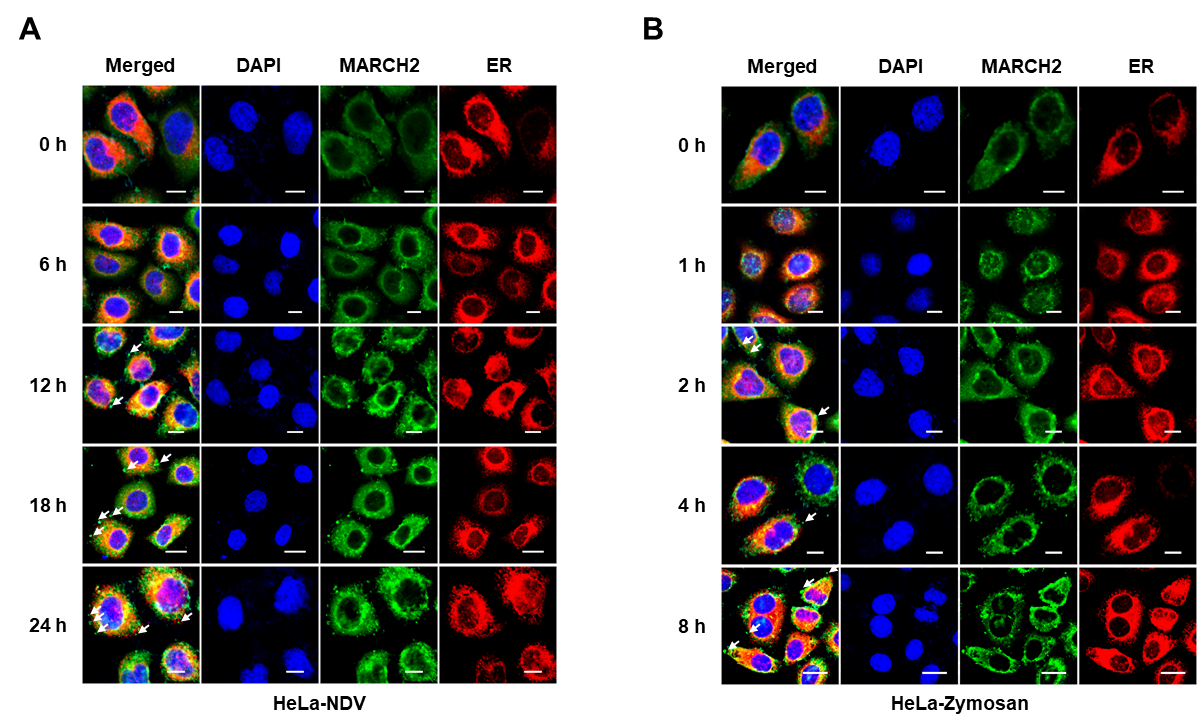
**

**Appendix Figure S6. MARCH2 localizes on and out of ER membrane at late time of pathogen infection or PAMP stimulation.**

**A, B** Localization of MARCH2 (green) with ER (red) in HeLa cells. NDV infection (A, MOI=1) or zymosan treatment (100μg/ml) in HeLa cells expressing TLR2 (B). Scale bar, 10μm. Arrow, non-localized MARCH2 protein in ER.

Data information: Data are representative of three independent experiments, each with similar results.

**
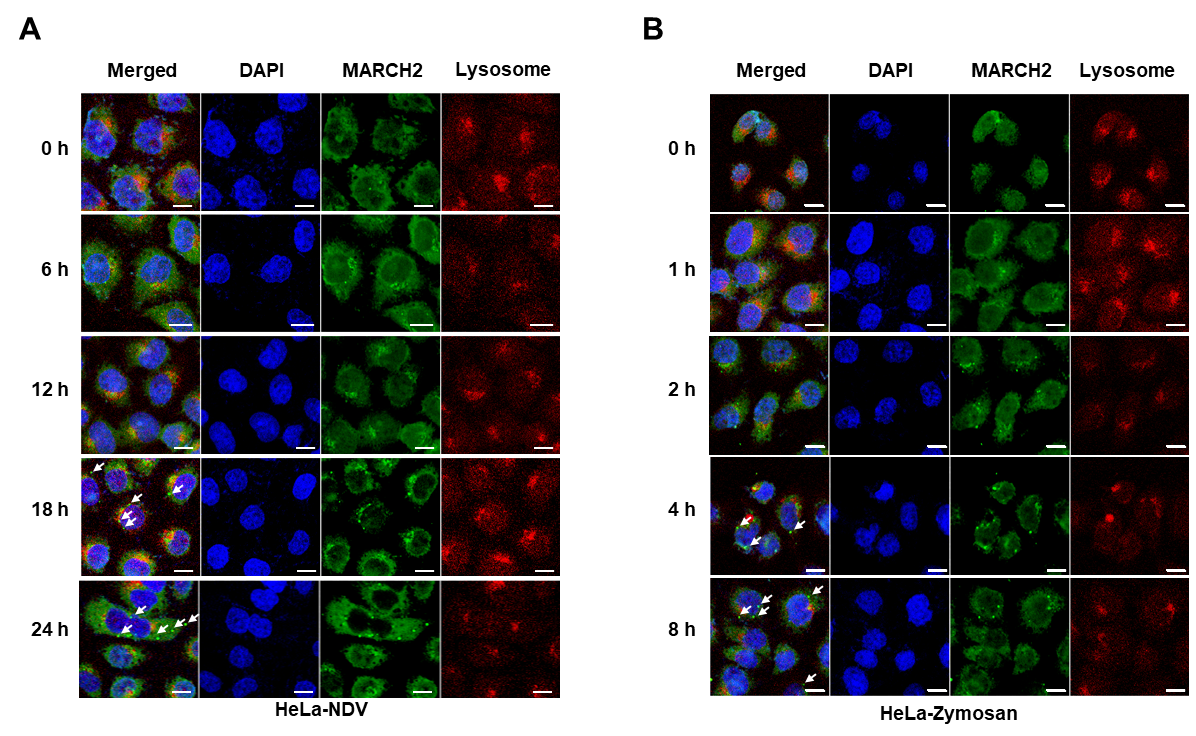
**

**Appendix Figure S7. MARCH2 localizes on and out of lysosome at late time of pathogen infection or PAMP stimulation.**

**A, B** Localization of MARCH2 (green) with lysosome (red) in HeLa cells. NDV infection (A, MOI=1) or zymosan treatment (100μg/ml) in HeLa cells expressing TLR2 (B). Scale bar, 10μm. Arrow, non-localized MARCH2 protein in lysosome.

Data information: Data are representative of three independent experiments, each with similar results.

**
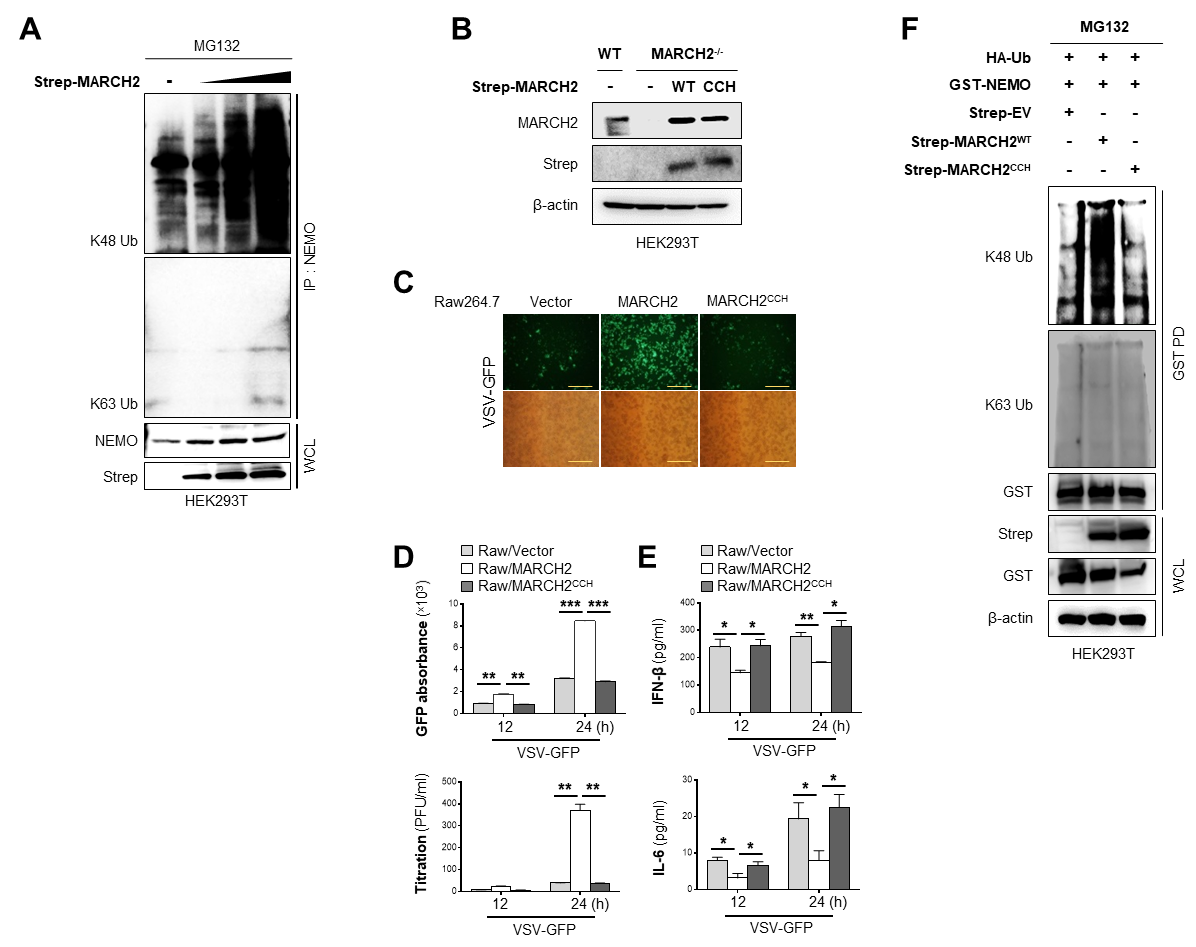
**

**Appendix Figure S8. E3 ubiquitin ligase activity of MARCH2 is important for negative regulation of innate immune response.**

**A** NEMO ubiquitination assay. HEK293T cells were transfected with different doses of Strep-tagged MARCH2. Whole cell lysates were immunoprecipitated with anti-NEMO antibody, followed by immunoblotting with anti-K48 or anti-K63 antibodies.

**B** Immunoblot analysis of whole cell lysates from MARCH2^+/+^, MARCH2^-/-^, MARCH2^-/-^ HEK293T reconstituted with Strep-tagged MARCH2 or MARCH2^CCH^. Blots were determined with anti-MARCH2 or anti-Strep antibody. β-actin was used to confirm equal loading of protein

**C-E** Raw264.7 cells harboring empty vector, Flag-tagged MARCH2, or MARCH2^CCH^ were infected with VSV-GFP (MOI=0.5). Viral replication (C, D). Levels of IFN-β and IL-6 in the cell supernatant (E), Scale bar, 50μm.

**F** HEK293T cells transfected with Strep-tagged empty vector, MARCH2 or MARCH2^CCH^ together with GST-tagged NEMO and HA-tagged ubiquitin under MG132 treatment were subjected to pull-down with GST beads, followed by immunoblotting with anti-Lys48 (K48)-linkage specific polyubiquitin (K48-ub), anti-Lys63 (K63)-linkage specific polyubiquitin (K63-ub), or anti-GST antibodies. Whole cell lysates were determined by immunoblotting with the indicated antibodies.

Data information: **P* < 0.05, ***P* < 0.01 (two-tailed Student’s *t-*test). Data are representative of at least two independent experiments, each with similar results, and expressed as the mean ± S.D. of two biological replicates.

Source data are available online for this figure.

**
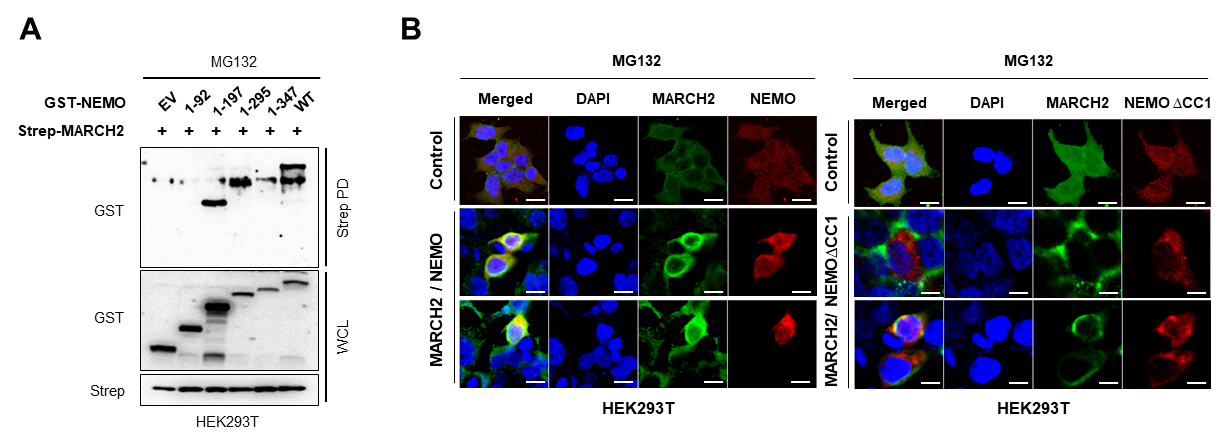
**

**Appendix Figure S9. NEMO CC1 domain is important for interaction with MARCH2.**

**A** HEK293T cells were transfected with GST-tagged NEMO domain constructs together with Strep-tagged MARCH2. Whole cell lysates were subjected to pull down with Strep beads, followed by immunoblotting with anti-GST antibody, or determined by immunoblotting with the indicated antibodies.

**B** Co-localization between MARCH2 (green) and either NEMO-WT (red) or NEMO-∆CC1 (red) in HEK293T cells in presence of MG132. Scale bar, 10μm.

Data information: Data are representative of three independent experiments, each with similar results.

Source data are available online for this figure.

**Appendix Table S1: List of antibody used for immunoblotting experiments**

| **Target or material name** | **Manufacture** | **Catalog number** | **Source** | **Dilution** |
| --- | --- | --- | --- | --- |
| MARCH2 |  | Not applicable | Rabbit polyclonal | 1:200 |
| MARCH2 | abcam | Ab123136 | Rabbit polyclonal | 1:1000 |
| NEMO (DA10-12) | Cell Signaling Technology | 2695 | Mouse monoclonal | 1:3000 |
| K48-Ubi | Millipore | 05-1305 | Rabbit monoclonal | 1:1000 |
| K63-Ubi | Millipore | 05-1308 | Rabbit monoclonal | 1:1000 |
| IKKα | Santa Cruz Biotechnology | sc-7606 | Mouse polyclonal | 1:1000 |
| IKKβ (D30C6) | Cell Signaling Technology | 8943S | Rabbit monoclonal | 1:1000 |
| HA-Probe(Y-11) | Santa Cruz | sc-805 | Rabbit polyclonal | 1:3000 |
| FLAG | Sigma | F1804 | Mouse monoclonal | 1:3000 |
| GST (26H1) | Cell Signaling Technology | 2624S | Mouse monoclonal | 1:3000 |
| Strep | IBA | 2-1509-001 | Mouse monoclonal | 1:5000 |
| P-IRF3 (S396) (4D4G) | Cell Signaling Technology | 4947S | Rabbit monoclonal | 1:1000 |
| IRF3 (D83B9) | Cell Signaling Technology | 4302S | Rabbit monoclonal | 1:1000 |
| P-P65 (Ser536) (93H1) | Cell Signaling Technology | 3033S | Rabbit monoclonal | 1:1000 |
| P65 (C22B4) | Cell Signaling Technology | 4764 | Rabbit monoclonal | 1:1000 |
| P-TBK1 (Ser172) (D52C2) | Cell Signaling Technology | 5483S | Rabbit monoclonal | 1:1000 |
| TBK1/NAK (D1B4) | Cell Signaling Technology | 3504S | Rabbit monoclonal | 1:1000 |
| P-IҡBα (Ser32) (14D4) | Cell Signaling Technology | 2859S | Rabbit monoclonal | 1:1000 |
| IҡBα | Cell Signaling Technology | 9242S | Rabbit monoclonal | 1:1000 |
| NA/K ATPase beta 1 | Gene Tex | GTX113390 | Rabbit polyclonal | 1:1000 |
| β-actin | BD Biosciences | 610153 | Mouse monoclonal | 1:3000 |
| HRP-conjugated anti-mouse IgG | Gene Tex | GTX213111-01 | Goat | 1:3000 |
| HRP-conjugated anti-rabbit IgG | Cell Signaling Technology | 7074S | Goat | 1:3000 |

**Appendix Table S2: List of antibody used for immunofluorescence experiments**

| **Name of the antibody** | **Manufacture** | **Catalog number** | **Source** | **Dilution** |
| --- | --- | --- | --- | --- |
| MARCH2 |  | Not applicable | Rabbit polyclonal | 1:50 |
| NEMO (DA10-12) | Cell Signaling Technology | 2695 | Mouse monoclonal | 1:200 |
| Calnexin (E-10) | Santa Cruz Biotechnology | sc-46669 | Mouse monoclonal | 1:100 |
| Cy3-conjugated donkey anti-mouse  IgG | The Jackson Laboratory | 715-165-150 | Mouse | 1:400 |
| Alexa 488 goat anti-rabbit IgG | Invitrogen | A11034 | Rabbit | 1:400 |

**Appendix Table S3: List of primers used for MARCH2 mice genotyping**

| **Product size (Base pair)** | **Forward primer** | **Reverse primer** |
| --- | --- | --- |
| 582 bp | ATCTCTGGAAGCTGGGTGTG | TTGGACCTCCTGACCCTAGA |
| 264 bp | TGACGACAGGTGACTGTTGC | TTGGACCTCCTGACCCTAGA |

**Appendix Table S4: List of primers used for site-directed mutagenesis**

| **Amino acid** | **Forward primer** | **Reverse primer** |
| --- | --- | --- |
| MARCH2 C64S | CACCGAGTGATGGTCCTTTCAGCCGGATCTGCCATGAGGG | CCCTCATGGCAGATCCGGCTGAAAGGACCATCACTCGGTG |
| MARCH2 C67S | GATGGTCCTTTCAGCCGGATCAGCCATGAGGGAGCGAACGGG | CCCGTTCGCTCCCTCATGGCTGATCCGGCTGAAAGGACCATC |
| MARCH2 H90Q | GGCACGCTGGGTGCCGTGCAAAAGAGCTGTCTGGAGAAGTG | CACTTCTCCAGACAGCTCTTTTGCACGGCACCCAGCGTGCC |
| NEMO K302R | GAGACCGTTCCGGTGCTGAAGGCCCAGGCGGATATC | GATATCCGCCTGGGCCTTCAGCACCGGAACGGTCTC |
| NEMO K309R | GCGGATATCTACAGGGCGGACTTC | GAAGTCCGCCCTGTAGATATCCGC |
| NEMO K321R | CAGGCCCGGGAGAGGCTGGCCGAGAAGAAG | CTTCTTCTCGGCCAGCCTCTCCCGGGCCTG |
| NEMO K325R | AAGCTGGCCGAGAGGAAGGAGCTC | GAGCTCCTTCCTCTCGGCCAGCTT |
| NEMO K326R | CTGGCCGAGAAGAGGGAGCTCCTG | CAGGAGCTCCCTCTTCTCGGCCAG |
| NEMO K342R | CAGAGGGAGTACAGCAAACTGAAGGCCAGCTGTCAG | CTGACAGCTGGCCTTCAGTTTGCTGTACTCCCTCTG |
| NEMO K344R | TACAGCAAACTGAGGGCCAGCTGT | ACAGCTGGCCCTCAGTTTGCTGTA |
| NEMO K358R | GCCAGGATCGAGGACATGAGGAAGCGGCATGTCGAGGTC | GACCTCGACATGCCGCTTCCTCATGTCCTCGATCCTGGC |

**Appendix Table S5: List of primers used for real time PCR**

| **Target gene** | **Forward primer** | **Rivers primer** |
| --- | --- | --- |
| IFN-β | TCCAAGAAAGGACGAACATTCG | TGCGGACATCTCCCACGTCAA |
| IFN-α | ATAACCTCAGGAACAACAG | TCATTGCAGAATGAGTCTAGGAG |
| ISG-15 | CAATGGCCTGGGACCTAAA | CTTCTTCAGTTCTGACACCGTCAT |
| ISG-20 | AGAGATCACGGACTACAGAA | TCTGTGGACGTGTCATAGAT |
| ISG-56 | AGAGAACAGCTACCACCTTT | TGGACCTGCTCTGAGATTCT |
| P56 | TCAAGTATGGCAAGGCTGTG | GAGGCTCTGCTTCTGCATCT |
| OAS-1β | AGGTGGTAAAGGGTGGCT | TGCTTGACTAGGCGGATG |
| OSA-1 | GAGGCGGTTGGCTGAAGAGG | GAGGAAGGCTGGCTGTGATTGG |
| Mx1 | ACAAGCACAGGAAACCGTATCAG | AGGCAGTTTGGACCATCTTAGTG |
| GBP1 | AAAAACTTCGGGGACAGCTT | CTGAGTCACCTCATAAGCCAAA |
| IL6 | GACAACTTTGGCATTGTGG | ATGCAGGGATGATGTTCTG |
| IL12 | CAGAAGCTAACCATCTCCTGGTTTG | TCCGGAGTAATTTGGTGCTTCACAC |
| IL1- β | TTGTGGCTGTGGAGAAGCTGT | AACGTCACACACCAGCAGGTT |
| CXCL10 | GCCGTCATTTTCTGCCTCA | CGTCCTTGCGAGAGGGATC |
| TNF-α | AGCAAACCACCAAGTGGAGGA | GCTGGCACCACTAGTTGGTGGT |
| COX-2 | TGAGTACCGCAAACGCTTCT | CTCCCCAAAGATAGCATCTGG |
| iNOS | TGGGAATGGAGACTGTCCCAG | GGGATCTGAATGTGATGTTTG |
| MARCH2-Mouse | ATGACGACAGGTGACTGTTGC | CCGAATCTTCAGGCGGACTTTC |
| GAPDH Mouse | TGACCACAGTCCATGCCATC | GACGGACACATTGGGGGTAG |
| GAPDH Human | GTAAAGTGGATATTGTTGCCATCA | AAATTCGTTGTCATACCAGGAAAT |

**Appendix Table S6: List of MARCH2 siRNA sequence**

| Forward primer | Reverse primer |
| --- | --- |
| GGAAGACAGCCAUUUCUCC | GGAGAAAUGGCUGUCUUCC |
