# Supplementary material for: Negative regulation of NEMO signaling by the ubiquitin E3 ligase MARCH2
Source: EMBO J. 2020 Sep 16;39(21):e105139. doi: 10.15252/embj.2020105139 (PMC7604578; doi:10.15252/embj.2020105139)
Supplement: Supplementary file 2 — Expanded View Figures PDF [file EMBJ-39-e105139-s002.pdf]

Expanded View Figures

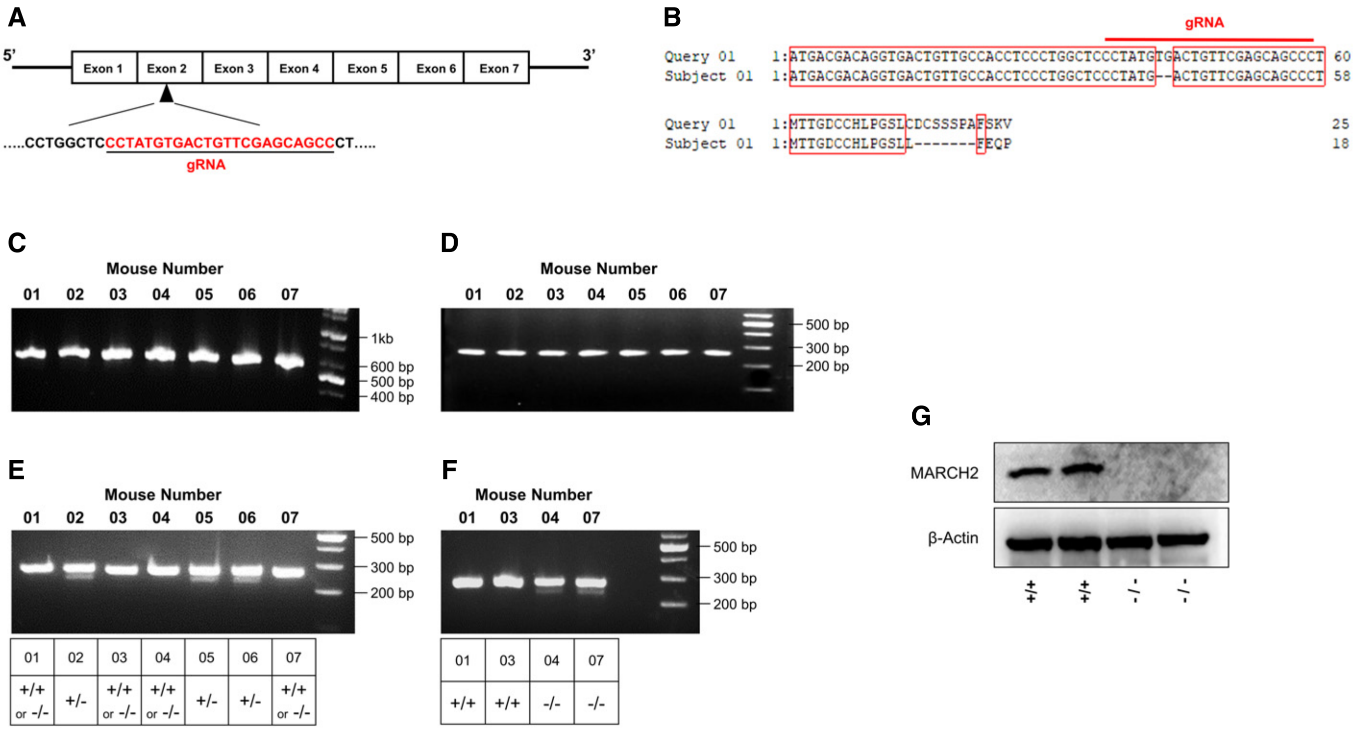

Figure EV1. MARCH2 knockout mice generation.

A Schematic representation of the genomic target site in the MARCH2 gene and gRNA sequence.

B Sequencing alignment result of MARCH2 gene and mice genomic DNA.

C–F Genotyping of MARCH2 mice (F2 generation). Heterozygous MARCH2$^{+/-}$ mice (F1 generation) were mated, and genomic DNA was extracted from tail ends from the pups (F2 generation) (C, D) Agarose gel electrophoresis of nested PCR products. First PCR product size, 582 bp (C). Second PCR product size, 264 bp (D). (E) Agarose gel electrophoresis of melted, reannealed, and T7 endonuclease 1-treated DNA. (F) MARCH2$^{+/+}$ or MARCH2$^{-/-}$ mice DNA was (Mouse number: 1, 3, 4, 7) individually mixed with equal amount of MARCH2$^{+/+}$ DNA then, melted, and reannealed, and T7 endonuclease 1 was treated.

G BMDMs isolated from MARCH2$^{+/+}$ and MARCH2$^{-/-}$ mice were infected with PR8-GFP (MOI = 3) virus. Whole-cell lysates were used for immunoblot with anti-MARCH2 antibody, which is normalized by β-actin.

Data information: (+/+, wild-type; +/−, heterozygous; −/−, knockout), gRNA: guide RNA.

Source data are available online for this figure.

© The Authors

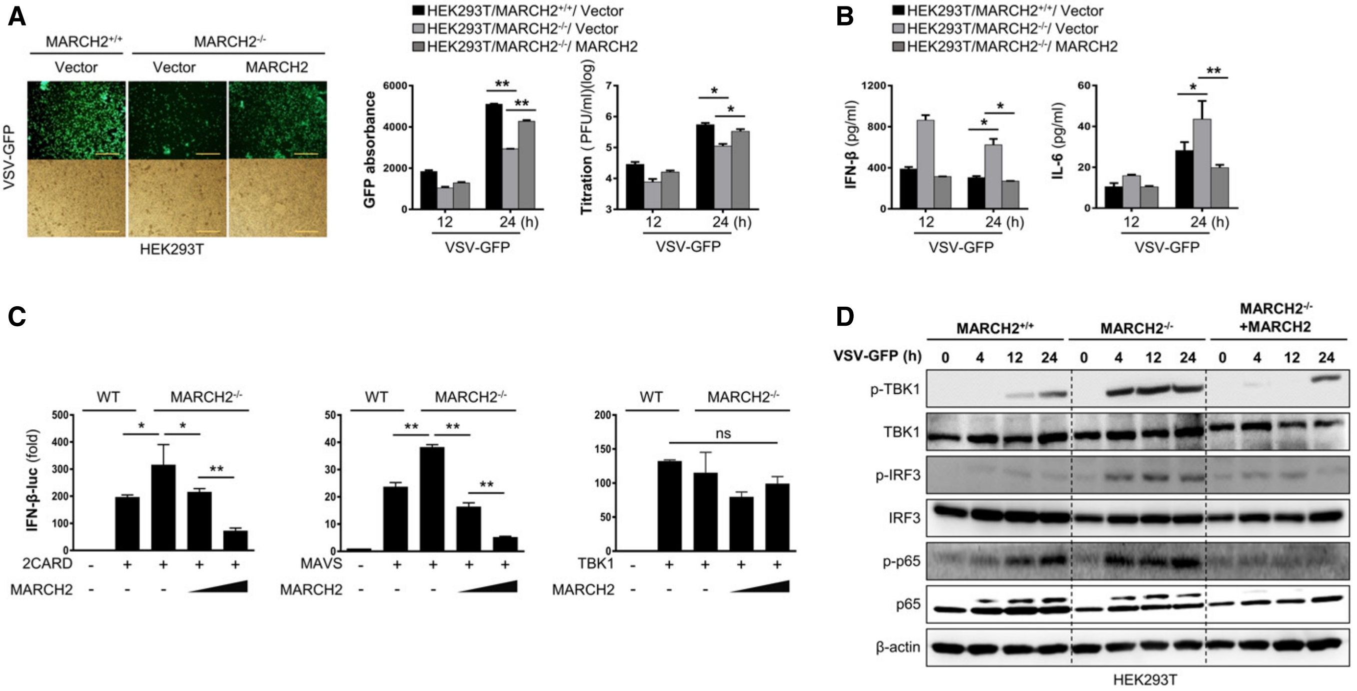

Figure EV2. Reconstitution of MARCH2 in MARCH2$^{-/-}$ HEK293T cells reduces the immune response leading to increased virus replication.

A, B MARCH2$^{+/+}$ HEK293T cells transfected with Flag-tagged empty vector or MARCH2$^{-/-}$ HEK293T cells transfected with Flag-tagged empty vector or MARCH2^{WT} were infected with VSV-GFP (MOI = 0.5). (A) Viral replication was determined at 24 hpi by fluorescence microscopy, fluorescence absorbance, and plaque assay. (B) Concentration of IFN-β and IL-6 secreted in supernatants was determined at 12 and 24 hpi by ELISA, Scale bar, 50 μm.

C IFN-β luciferase reporter assay. MARCH2$^{+/+}$ or MARCH2$^{-/-}$ HEK293T cells were transfected with firefly luciferase reporter plasmid encoding INF-β promoter, TK-Renilla plasmid, and expression plasmids of RIG-I 2CARD, MAVS, or TBK-1. MARCH2$^{-/-}$ HEK293T cells were reconstituted with increasing amount of Flag-tagged MARCH2 plasmid (100, 200 ng).

D MARCH2$^{+/+}$, MARCH2$^{-/-}$, or MARCH2$^{-/-}$ HEK293T reconstituted with Flag-tagged MARCH2^{WT} were infected with VSV-GFP (MOI = 0.5). Cells were harvested at indicated hours post-infection; total and phosphorylated TBK1, IRF3, and p65 were measured by immunoblotting in whole-cell lysates. β-actin was used to confirm equal loading of protein.

Data information: *$P < 0.05$, **$P < 0.01$ (two-tailed Student's *t*-test). Data are representative of at least two independent experiments, each with similar results, and expressed as the mean ± SD of two biological replicates.

Source data are available online for this figure.

 © The Authors

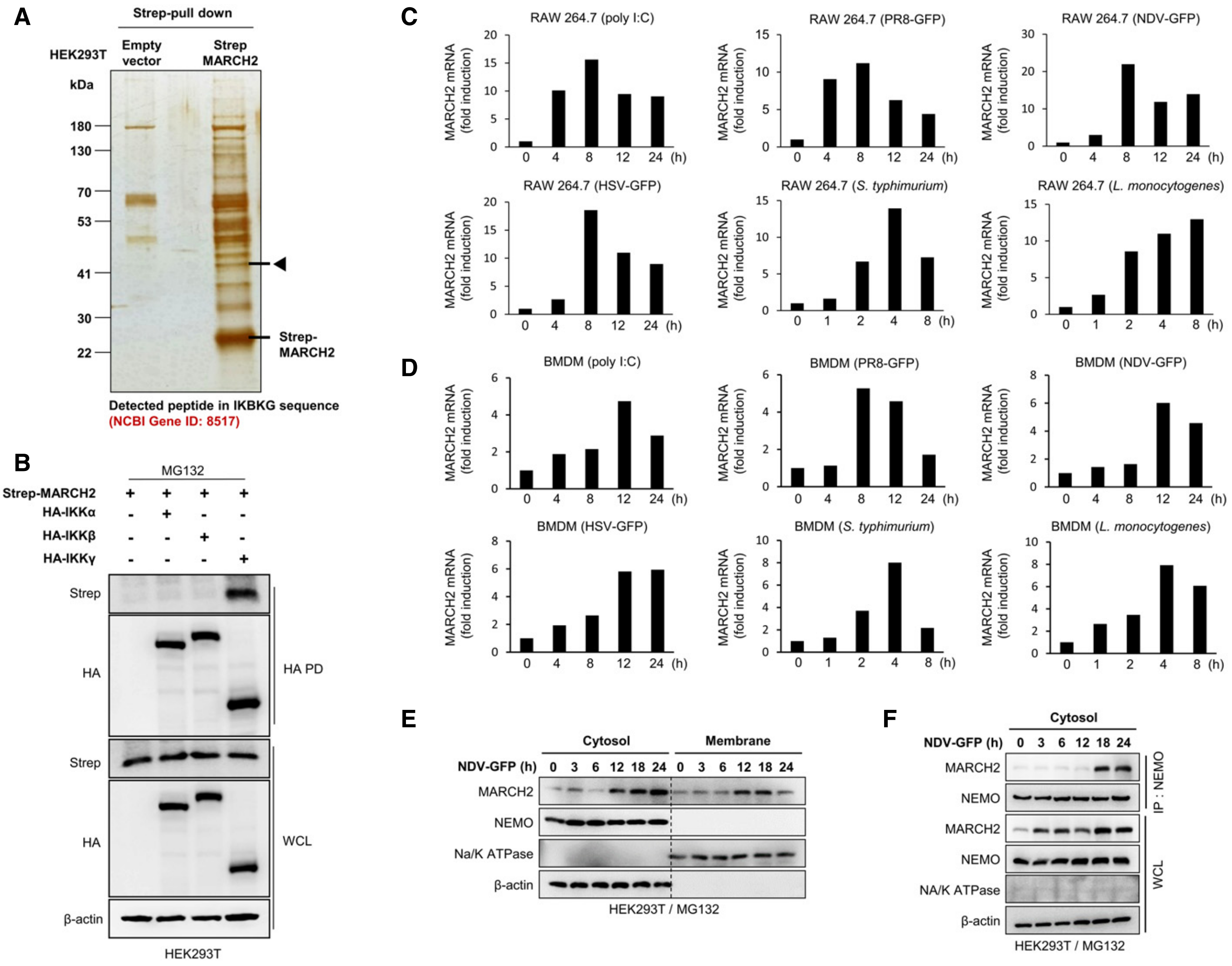

Figure EV3. MARCH2 is expressed by pathogen infection and interacts with NEMO in cytosol.

A Silver staining for MARCH2 interactome assay. Arrow indicates the expected protein expression of NEMO.

B HEK293T cells transfected with Strep-tagged MARCH2 together with HA-tagged IKKα, IKKβ, or NEMO under MG132 treatment were subjected to immunoprecipitation with an anti-HA antibody, followed by immunoblot with anti-Strep antibody or anti-HA antibody. Whole-cell lysates were determined by immunoblotting with anti-Strep, anti-HA, or anti-β-actin antibody.

C, D MARCH2 expression upon virus and bacterial infection. RAW 264.7 cells (C) and BMDMs isolated from C57BL/6 mice (D) were treated with poly I:C or infected with PR8-GFP, NDV-GFP, HSV-GFP, *S. typhimurium*, and *Listeria monocytogenes*, followed by qPCR with MARCH2 gene-specific primers.

E, F HEK293T cells were infected with NDV-GFP (MOI = 1) under MG132 treatment and harvested at indicated time points. (E) Protein expression in cytosolic and total membrane fraction was determined by immunoblotting with anti-MARCH2, anti-NEMO, anti-Na/K ATPase, or anti-β-actin antibody. (F) Cytosolic fraction was subjected to immunoprecipitation with an anti-NEMO antibody, followed by immunoblotting with anti-MARCH2 or anti-NEMO antibody. Protein expression in total cytosolic fraction was determined by immunoblotting with anti-MARCH2, anti-NEMO, anti-Na/K ATPase, or anti-β-actin antibody.

Data information: Data are representative of at least two independent experiments, each with similar results (B–F).

Source data are available online for this figure.

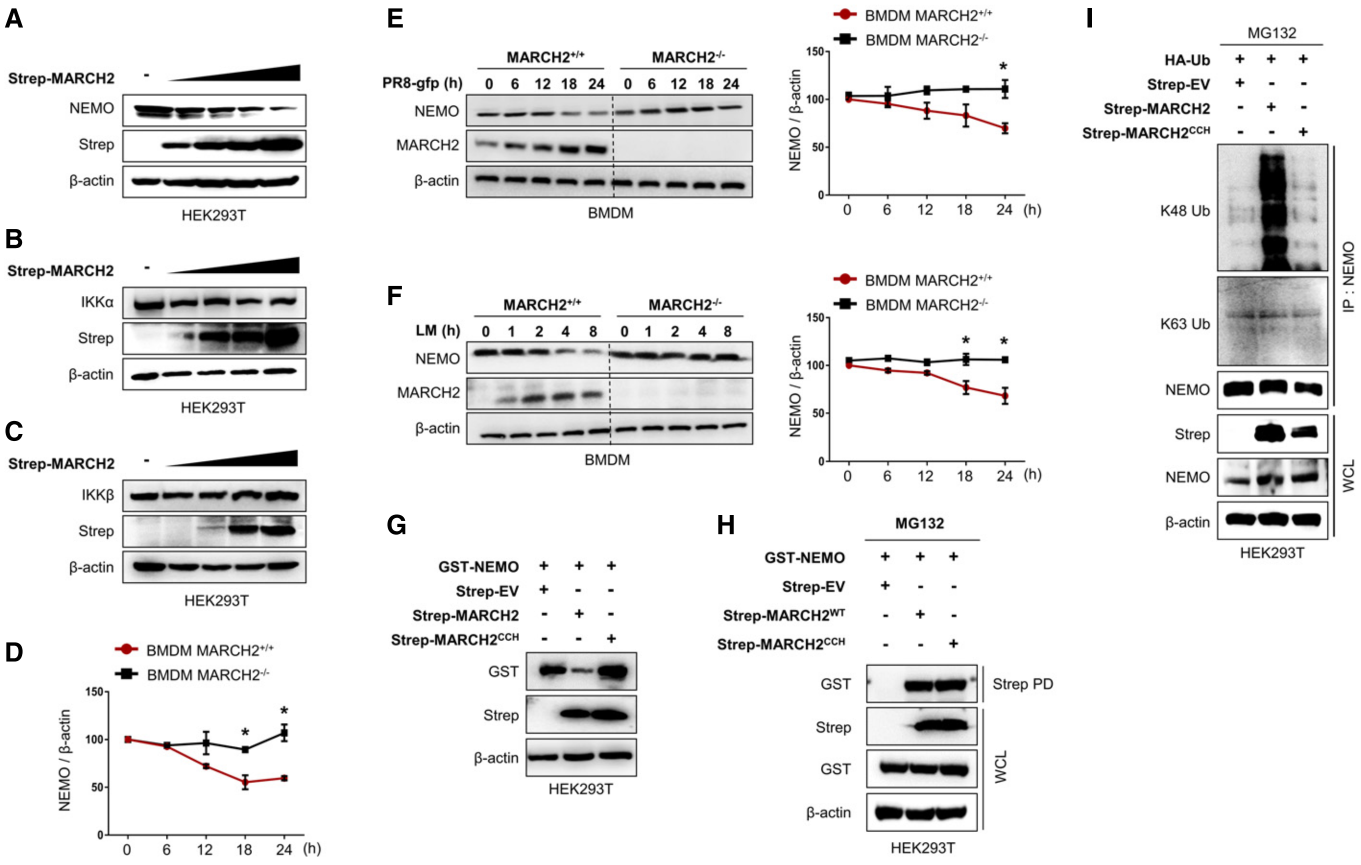

Figure EV4. MARCH2 induces NEMO degradation via E3 ubiquitin ligase function.

A–C HEK293T cells were transfected with an increasing amount of Strep-tagged MARCH2, followed by immunoblot with anti-NEMO (A), anti-IKKα (B), or anti-IKKβ (C) and the other indicated antibodies.

D Arbitrary value of band intensity of NEMO blot shown in Fig 5B.

E, F MARCH2$^{+/+}$ and MARCH2$^{-/-}$ BMDMs were infected with PR8-GFP (E, MOI = 3) or *Listeria monocytogenes* (F, MOI = 1) in time-dependent manner. Cells were lysed, where NEMO and MARCH2 expression was determined by immunoblotting. β-actin was used to confirm equal loading of protein. Band intensity of NEMO blots was shown in graphs (right).

G HEK293T cells were transfected with a Strep-tagged empty vector, MARCH2, or MARCH2^{CCH} together with GST-tagged NEMO. Whole-cell lysates were subjected to immunoblotting with the indicated antibodies.

H HEK293T cells transfected with Strep-tagged empty vector, MARCH2, or MARCH2^{CCH} together with GST-tagged NEMO were subjected to pull-down with Strep beads, followed by immunoblotting with anti-GST antibody. Whole-cell lysates were determined by immunoblotting with the indicated antibodies.

I HEK293T cells transfected with a Strep-tagged empty vector, MARCH2, or MARCH2^{CCH} together with HA-tagged ubiquitin were treated with MG132 (10 μM) for 6 h before harvest. Whole-cell lysates were subjected to immunoprecipitation with an anti-NEMO antibody, followed by immunoblotting with anti-K48 or anti-K63 linkage-specific polyubiquitin antibodies

Data information: *$P < 0.05$ (two-tailed Student's *t*-test). Data are representative of at least two independent experiments, each with similar results, and expressed as the mean ± SD of two biological replicates.

Source data are available online for this figure.

 © The Authors

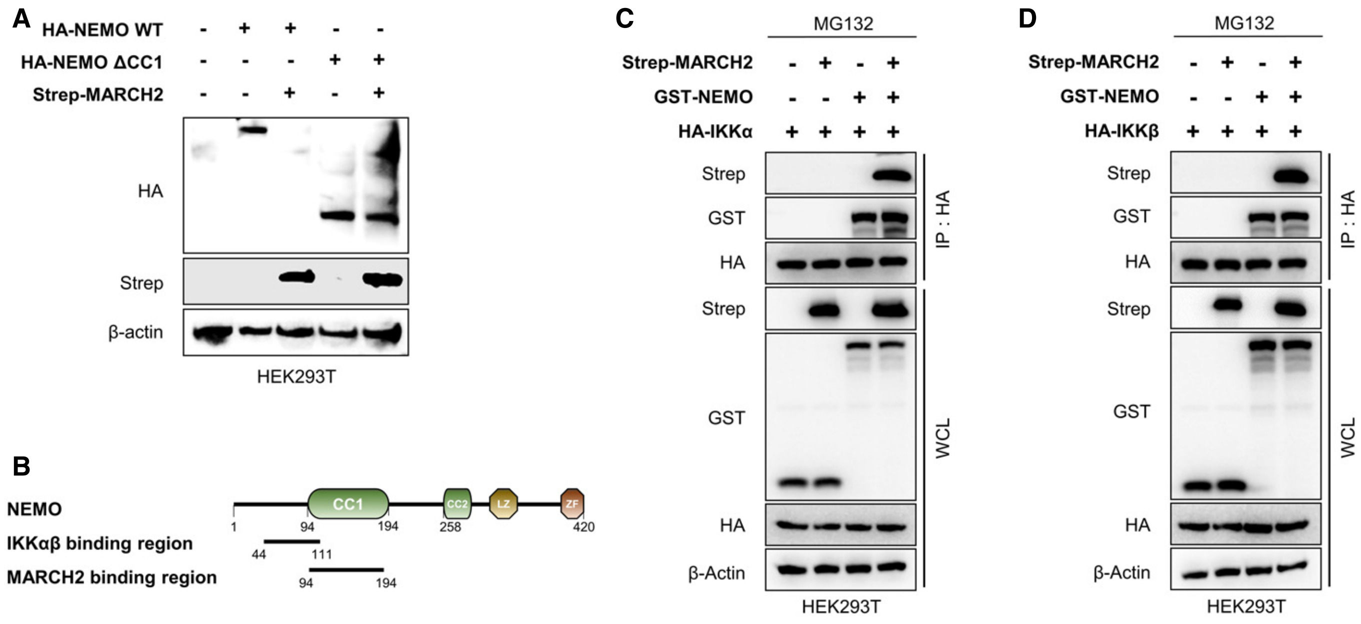

Figure EV5. MARCH2 leads to NEMO degradation, but not its dissociation from IKK complex via interaction with CC1 domain.

A HEK293T cells were transfected with HA-tagged NEMO-WT or NEMO-ΔCC1 in the absence or presence of Strep-tagged MARCH2. Whole-cell lysates were subjected to immunoblotting with anti-HA, anti-Strep, or anti-β-actin antibody.

B Schematic presentation of NEMO domain, interacting with IKK complex (IKKα, IKKβ) or MARCH2.

C, D HEK293T cells transfected with HA-tagged (C) IKKα or (D) IKKβ together with indicated plasmids were treated with MG132 (10 μM) for 6 h before harvest. Whole-cell lysates were subjected to immunoprecipitation with an anti-HA antibody, followed by immunoblotting with an anti-HA, anti-Strep, or anti-GST antibody. Whole-cell lysate was subjected to above antibody together with anti-β-actin.

Data information: Data are representative of at least two independent experiments, each with similar results.

Source data are available online for this figure.
