# Supplementary figures and images for: Negative regulation of NEMO signaling by the ubiquitin E3 ligase MARCH2
Source: EMBO J. 2020 Sep 16;39(21):e105139. doi: 10.15252/embj.2020105139 (PMC7604578; doi:10.15252/embj.2020105139)

**Fig EV5A**

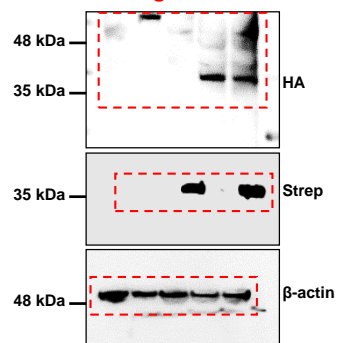

**Fig EV5C**

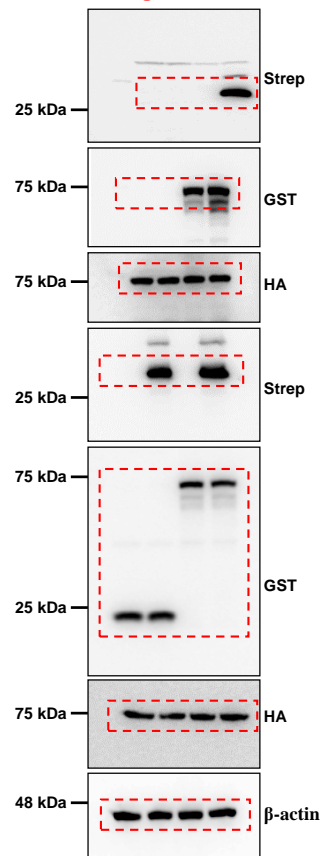

**Fig EV5D**

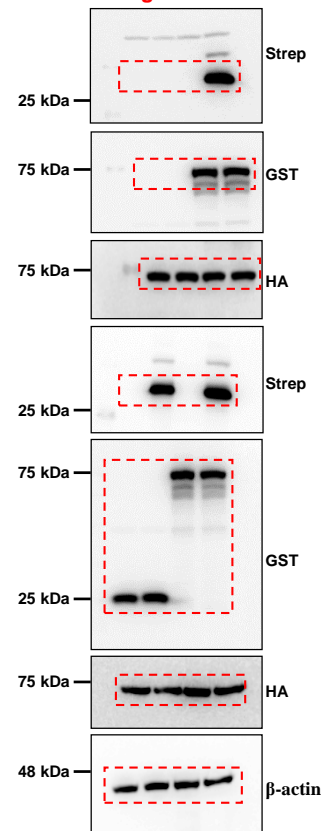

Supplement: Supplementary file 3 — Source Data for Expanded View and Appendix [file EMBJ-39-e105139-s010.zip › Appendix_and_EV_Figure_Source_Data/EV_figure_Source_Data/Source Data for Figure EV05.pdf]

Figure EV1C

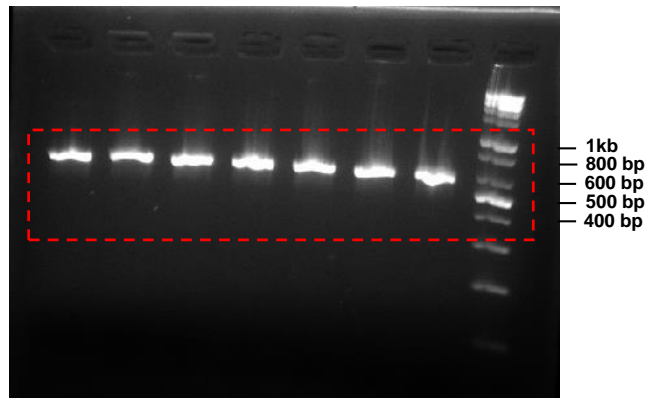

Figure EV1D

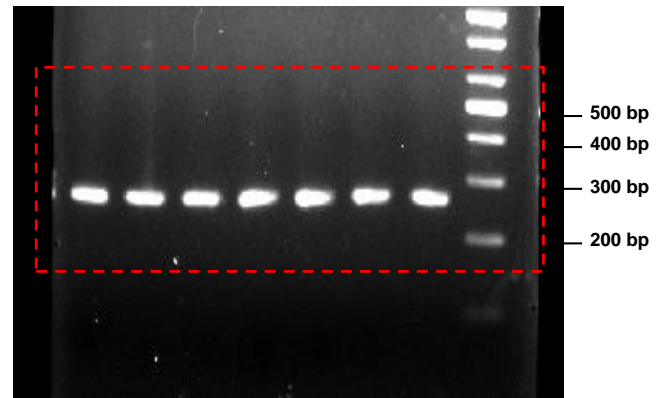

Figure EV1E

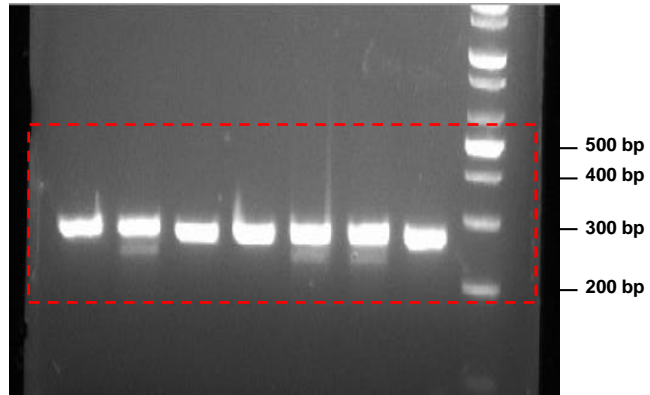

Figure EV1F

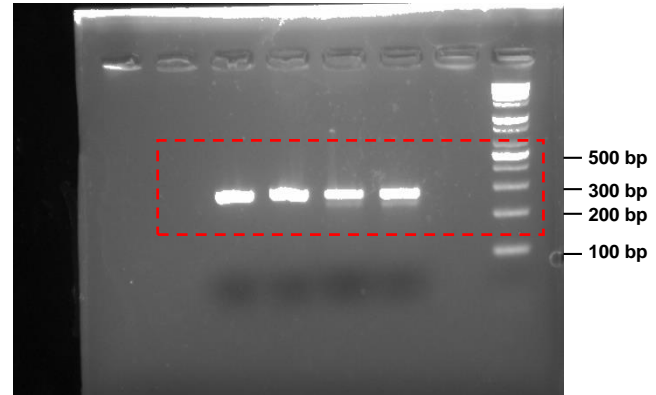

Figure EV1G

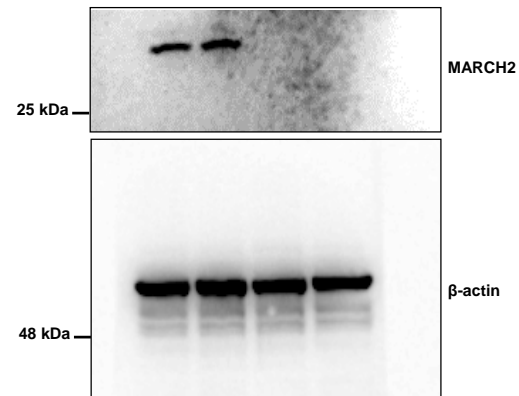

Supplement: Supplementary file 3 — Source Data for Expanded View and Appendix [file EMBJ-39-e105139-s010.zip › Appendix_and_EV_Figure_Source_Data/EV_figure_Source_Data/Source Data for Figure EV01.pdf]

Appendix Fig S9A

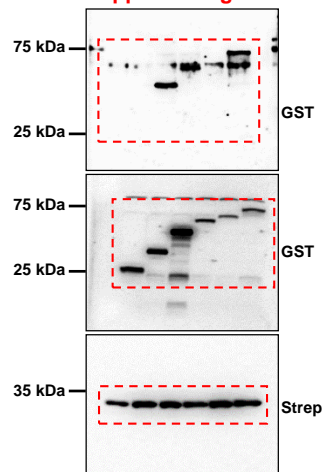

Supplement: Supplementary file 3 — Source Data for Expanded View and Appendix [file EMBJ-39-e105139-s010.zip › Appendix_and_EV_Figure_Source_Data/Appendix_Figure_Source_Data/Source Data for Appendix Figure S09.pdf]

**Appendix Fig S5A**

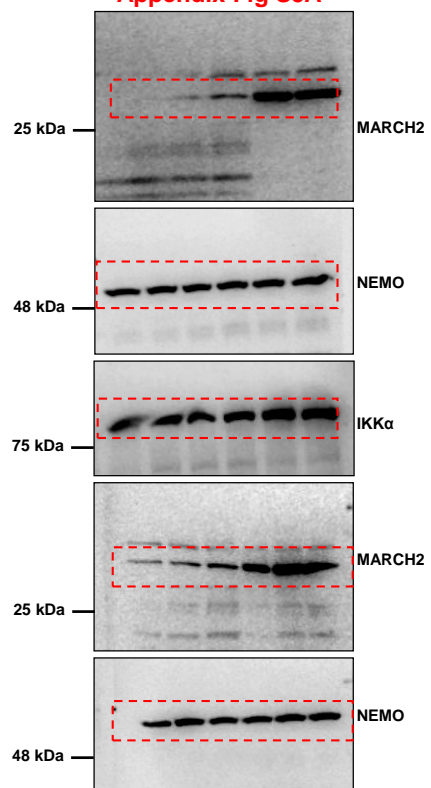

**Appendix Fig S5B**

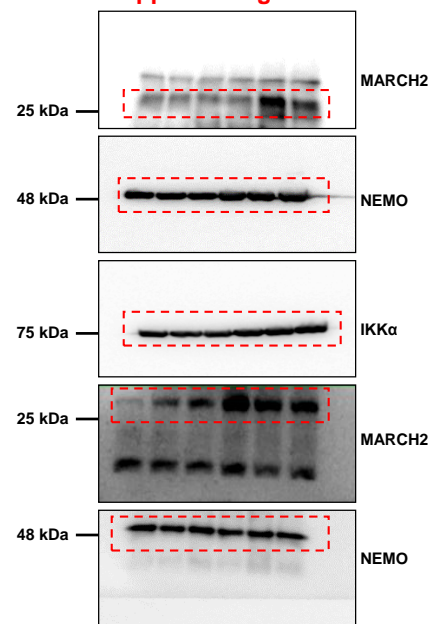

Supplement: Supplementary file 3 — Source Data for Expanded View and Appendix [file EMBJ-39-e105139-s010.zip › Appendix_and_EV_Figure_Source_Data/Appendix_Figure_Source_Data/Source Data for Appendix Figure S05.pdf]

**Fig 4e**

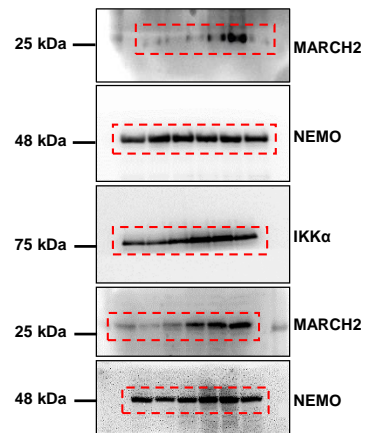

**Fig 4f**

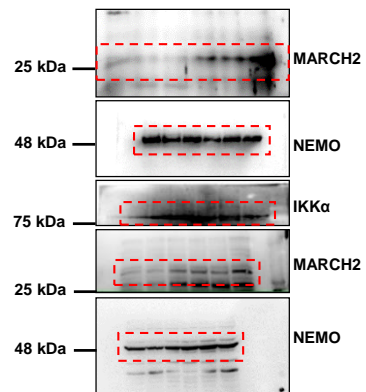

**Fig 4h**

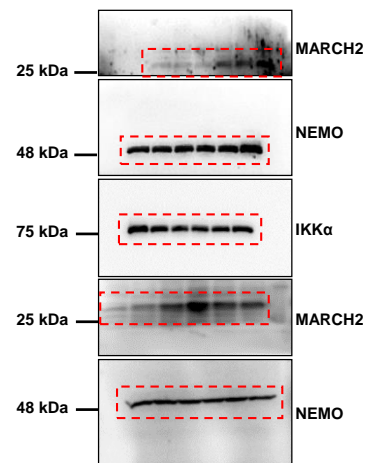

**Fig 4i**

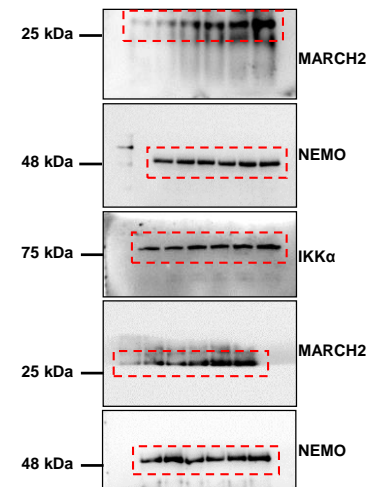

Supplement: Supplementary file 8 — Source Data for Figure 4 [file EMBJ-39-e105139-s006.zip › EMBOJ-2020-105139R-Source Data for Figure 04.pdf]

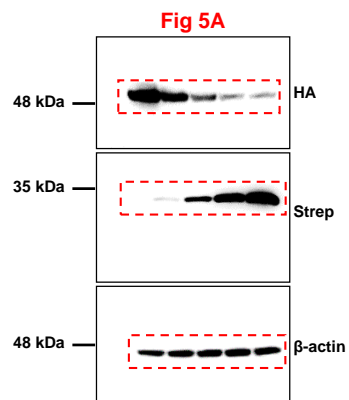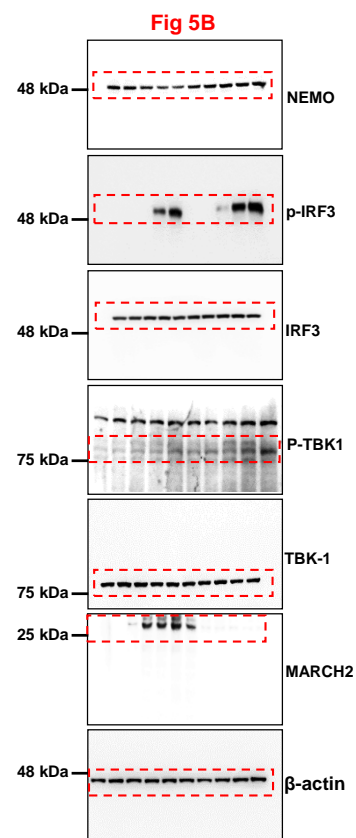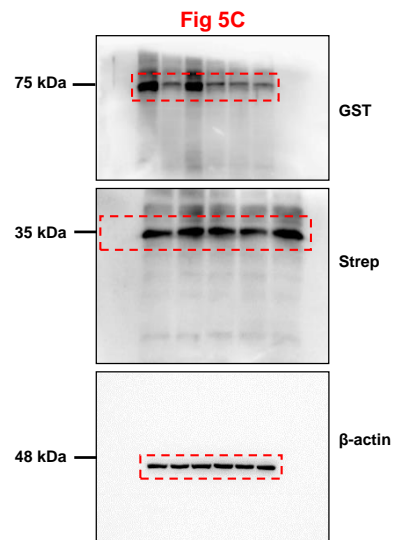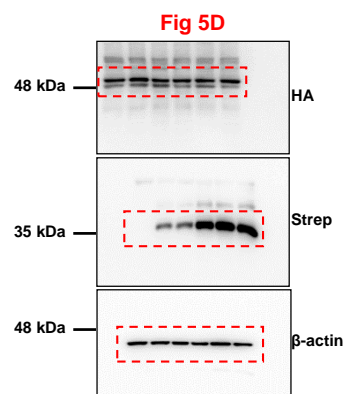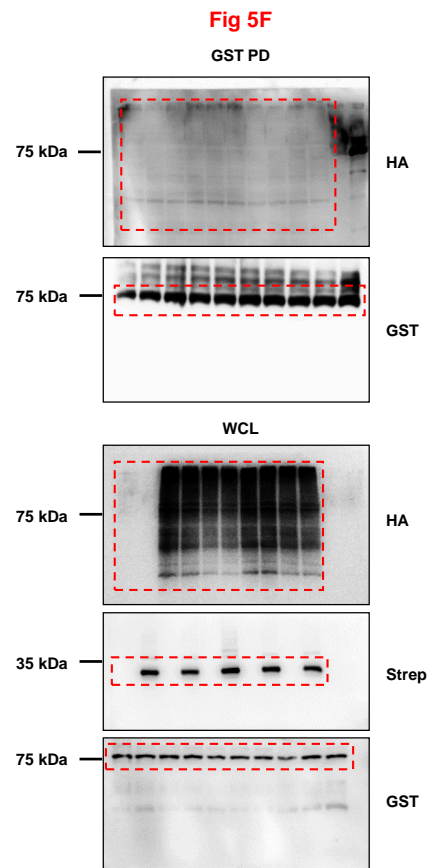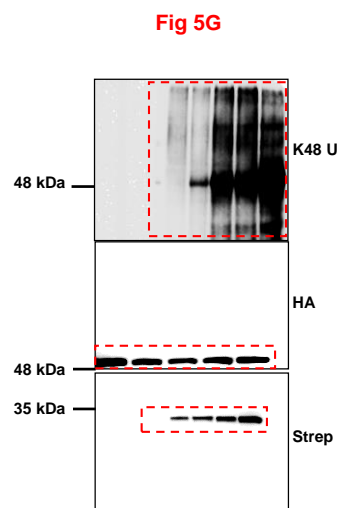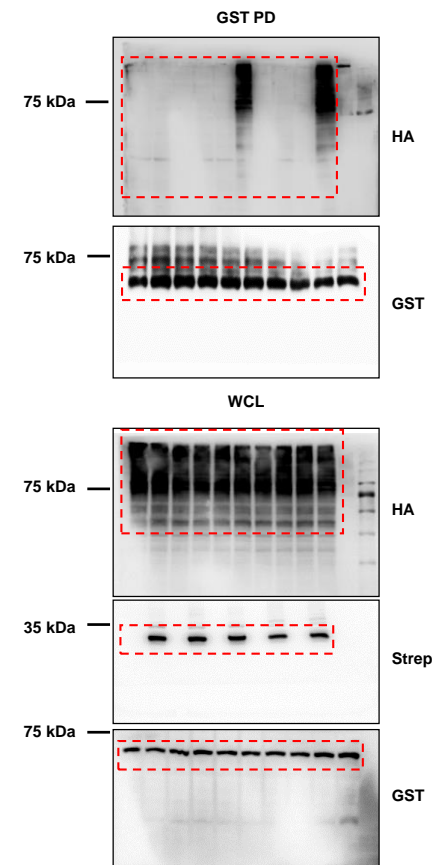

Supplement: Supplementary file 9 — Source Data for Figure 5 [file EMBJ-39-e105139-s007.zip › EMBOJ-2020-105139R-Source Data for Figure 05.pdf]

**Fig 6B**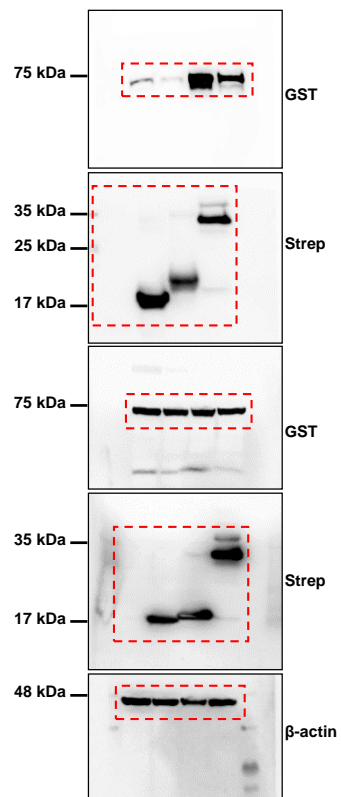**Fig 6D**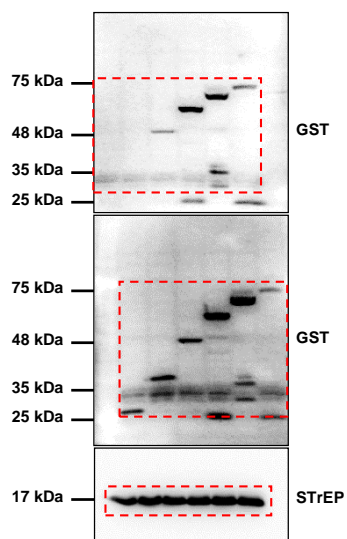**Fig 7F**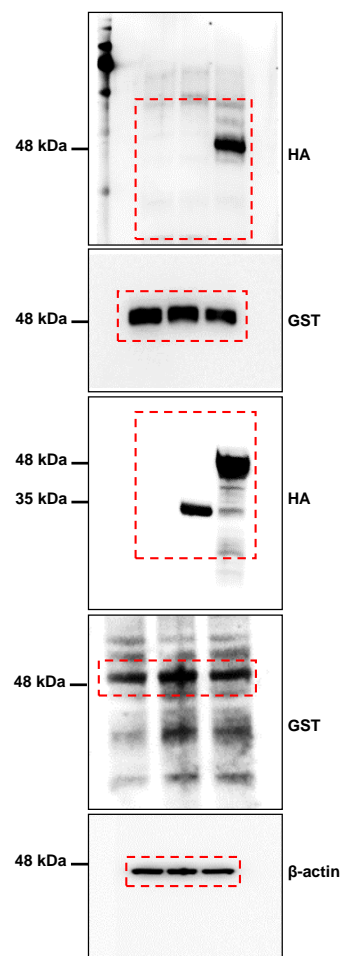**Fig 7G**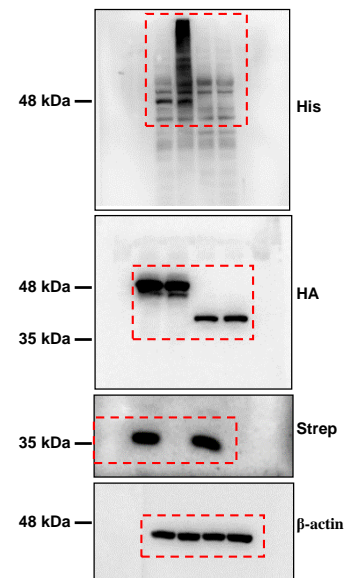**Fig 7J**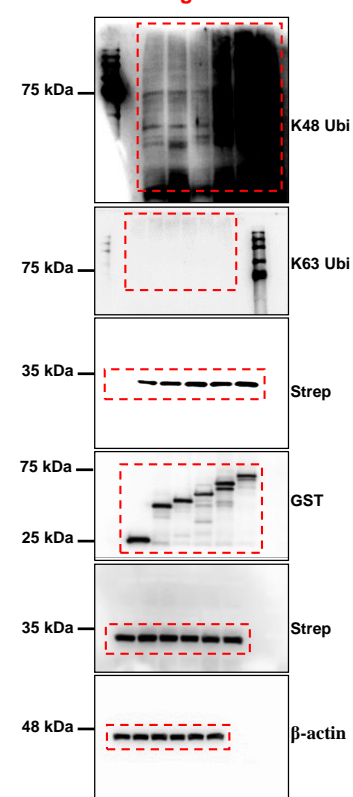**Fig 7I**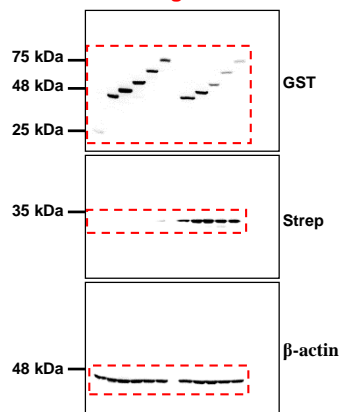

Supplement: Supplementary file 10 — Source Data for Figure 6 [file EMBJ-39-e105139-s008.pdf]

**Fig 7A**

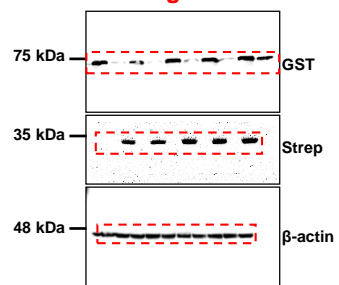

**Fig 7A**

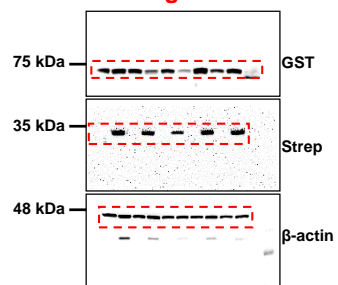

**Fig 7B**

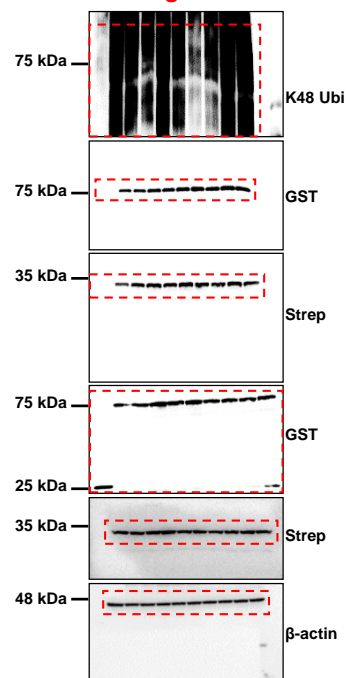

**Fig 7C**

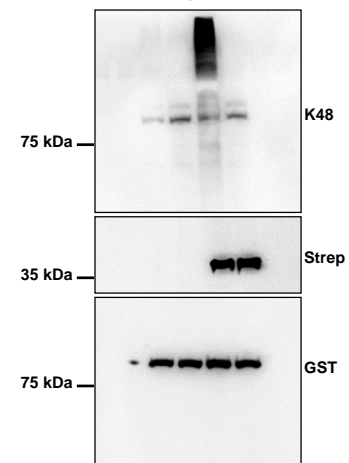

Supplement: Supplementary file 11 — Source Data for Figure 7 [file EMBJ-39-e105139-s009.zip › EMBOJ-2020-105139R-Source Data for Figure 07.pdf]
