# Supplementary material for: Negative regulation of NEMO signaling by the ubiquitin E3 ligase MARCH2
Source: EMBO J. 2020 Sep 16;39(21):e105139. doi: 10.15252/embj.2020105139 (PMC7604578; doi:10.15252/embj.2020105139)
Supplement: Supplementary file 4 — Review Proccess File [file EMBJ-39-e105139-s011.pdf]

THE EMBO JOURNAL

Negative regulation of the NEMO signaling by the ubiquitin E3 ligase MARCH2

Kiramage Chathuranga, Tae-Hwan Kim, Hyuncheol Lee, Jun-Seol Park, Jae-Hoon Kim, W. A. Gayan Chathuranga, Pathum Ekanayaka, Youn Jung Choi, Chul-Ho Lee, Chul-Joong Kim, and Jae U. Jung, Jong-Soo Lee
DOI: doi.org/10.15252/embj.2020105139

Corresponding author: Jong-Soo Lee (jongsool@cnu.ac.kr)

Review Timeline:

Submission Date:	29th Mar 20
Editorial Decision:	22nd Apr 20
Revision Received:	30th Jun 20
Editorial Decision:	28th Jul 20
Revision Received:	7th Aug 20
Accepted:	11th Aug 20

Editor: Karin Dumstrei

Transaction Report:

(Note: With the exception of the correction of typographical or spelling errors that could be a source of ambiguity, letters and reports are not edited. Depending on transfer agreements, referee reports obtained elsewhere may or may not be included in this compilation. Referee reports are anonymous unless the Referee chooses to sign their reports.)

1st Editorial Decision 22nd Apr 2020

Dear Jong-Soo,

Thank you for submitting your manuscript to The EMBO Journal. Your study has now been seen by two referees and their comments are provided below.

As you can see from the comments, the referees find the analysis interesting. However, they also find that the analysis needs to be extended and that we need further data to support that MARCH2 antagonizes innate immunity via NEMO regulation. Should you be able to address the raised concerns in full then we can consider a revised version. I am happy to discuss the raised points further and maybe it would be most helpful to do so via phone or skype. I will contact you in the next few days to discuss this further.

When preparing your letter of response to the referees' comments, please bear in mind that this will form part of the Review Process File, and will therefore be available online to the community. For more details on our Transparent Editorial Process, please visit our website: https://www.embopress.org/page/journal/14602075/authorguide#transparentprocess

I thank you for the opportunity to consider your work for publication. I look forward to discuss the revisions further with you.

Yours sincerely,

Karin

Karin Dumstrei, PhD
Senior Editor
The EMBO Journal

Instructions for preparing your revised manuscript:

Please make sure you upload a letter of response to the referees' comments together with the revised manuscript.

Please also check that the title and abstract of the manuscript are brief, yet explicit, even to non-specialists.

When assembling figures, please refer to our figure preparation guideline in order to ensure proper formatting and readability in print as well as on screen:
http://bit.ly/EMBOPressFigurePreparationGuideline

IMPORTANT: When you send the revision we will require
- a point-by-point response to the referees' comments, with a detailed description of the changes made (as a word file).
- a word file of the manuscript text.
- individual production quality figure files (one file per figure)

- a complete author checklist, which you can download from our author guidelines (https://www.embopress.org/page/journal/14602075/authorguide).
- Expanded View files (replacing Supplementary Information)
Please see out instructions to authors
https://www.embopress.org/page/journal/14602075/authorguide#expandedview

Please remember: Digital image enhancement is acceptable practice, as long as it accurately represents the original data and conforms to community standards. If a figure has been subjected to significant electronic manipulation, this must be noted in the figure legend or in the 'Materials and Methods' section. The editors reserve the right to request original versions of figures and the original images that were used to assemble the figure.

Further information is available in our Guide For Authors:
https://www.embopress.org/page/journal/14602075/authorguide

The revision must be submitted online within 90 days; please click on the link below to submit the revision online before 21st Jul 2020.

Link Not Available

Please do not share this URL as it will give anyone who clicks it access to your account.

Referee #1:

In the present manuscript, Chathuranga et al identify the E3 ligase MARCH2 as a negative regulator of innate immune responses against viral and bacterial infection. Mechanistically, they show that MARCH2 directly associates with NEMO/IKKg, the essential regulator of canonical IkB kinase/NF-kB signaling. MARCH2 conjugates K48-linked ubiquitin chains to NEMO, which triggers its proteasomal degradation post-induction. Thus, the authors suggest that MARCH2 restricts anti-viral and anti-bacterial responses by limiting the amounts of NEMO and thus canonical NF-kB signaling upon innate immune stimulation.

The authors present compelling evidence that MARCH2 acts as a negative regulator of innate immune responses. These include the data on MARCH2-deficient mice as well as a different cellular models using knock-out, knock-down, overexpression and reconstitution. In addition, a role of MARCH2 for NEMO ubiquitination and degradation is documented by overexpression, knock-out/knock-downs, mutagenesis, mapping of the ubiquitin attachment, providing clear evidence that MARCH2 can act as a NEMO ubiquitin ligase.

My major concern is that both parts are not well-connected and that there is no clear functional evidence that MARCH2 antagonizes innate immunity via NEMO degradation. Previous studies showed that MARCH2 localizes largely to endosomal vesicles and the plasma membrane and anti-viral responses may still be connected for instance to the endosomal sorting machinery. To confirm that MARCH2 antagonizes innate immunity via NEMO binding/ubiquitination, I think the following points need be addressed:

1) It is shown that mutation of the ubiquitin acceptor site K326 in NEMO is abolishing the negative

regulatory function of MARCH2 on anti-viral responses in HEK293 cells, but there are no data showing that VSV-GFP infection is actually decreasing expression of NEMO wt, but not NEMO K326R. I think such data need to be included to lend support to the model.

2) With the exception in Figure 5c, NEMO degradation is only observed after MARCH2 overexpression. To confirm their mechanistic claim, the authors need to show that NEMO stability is increased in primary MARCH2-/- cells (e.g. BMDM) after viral infection (e.g. VSV) and/or upon PAMP recognition (e.g. poly(I:C), LPS, Lm). This is important, because at least I am not aware of publications demonstrating that NEMO is indeed prone to proteasomal degradation post-induction.

3) While loss of MARCH2 decreases IKK/NF-kB signaling, MARCH2 seems to have a stronger effect in counteracting TBK1 and IRF3 phosphorylation. Are the authors claiming that NEMO degradation is also enhancing TBK1-IRF3 signaling? If so, can the authors show that TBK1-IRF3 is controlled by NEMO in HEK293 cells? I think it is very likely that MARCH2 may have additional targets to control innate activation of TBK1/IRF pathway. It may go beyond the scope to identify these targets, but at least it needs to be discussed.

Specific points:

The description of the generation and validation of March2-/- mice is not acceptable. The description in Material and Methods is not sufficient to understand the KO strategy. What were the sgRNAs, what were expected genomic alterations and how did they verify these by PCR? Genotyping in Supplementary Figure 2 is not understandable and not helpful in its present form. A scheme showing targeting and verification should be included. Further, apparently the author do not have an antibody for detection of murine MARCH2. To confirm the expected effects, at least a decrease or truncation of March2 transcripts should be demonstrated on mRNA level.

Figure 4e, f, h, i: MARCH2 expression is induced by the stimulation. How is MARCH2 regulated? Is March2 mRNA induced or is this simply post-translational stabilization due to MG132 treatment?

Figure 7 and S9: Does MARCH2 bind to NEMO when bound to IKKs? MARCH2 binding to the CC1 domain of NEMO could also lead to dissociation of NEMO from IKKs.

Minor concern:

Are the Western Blots with a dashed line (e.g. 5c, S3a, c, S4e, S5h etc.) from different gels? If so, the Blots should be separated.

Additional comment:

I think the manuscript can be shortened and streamlined. For example, biochemical studies showing MARCH2-NEMO interaction and regulation are dispersed between Figures 5-8 and the studies can be better combined and shortened. Also, appearance of figures and the description in the text is not always aligned and this should be corrected.

Referee #2:

In this paper, Chathuranga et al. identified the ubiquitin ligase MARCH2 as a negative regulator of IFN and NF-κB signaling in response to viral and bacterial infection (or stimulation with PAMPs) in vitro and in vivo. The authors show that MARCH2 deficiency protects mice from viral or bacterial infection, accompanied by increased cytokine levels. Mechanistically, the authors report that MARCH2 catalyzes K48 ubiquitination of NEMO, leading to its proteasomal degradation. The experiments are clear and well controlled. Although these findings are novel and potentially interesting, there are several shortcomings that need to be addressed:

Major concerns:
- The authors suggest in the discussion that MARCH2-deficient mice could potentially suffer from autoimmunity or chronic inflammation. Yet control mice in their in vivo experiments seem to have cytokine levels similar to wild type mice. This is highly surprising given the phenotype of mice lacking other negative regulators of NF-κB, such as A20. It would be important to investigate/document in more detail whether MARCH2 deficiency causes any spontaneous autoimmune phenotype.
- In relation to the above comment (normal cytokine levels/absence of inflammation in control knockout group): does MARCH2 regulate only PAMP-initiated signaling? What about DAMPs (eg IL-1 stimulation)?
- The authors mention that MARCH2 interacts with NEMO at the late stages of infection, which coincides with increased expression levels of MARCH2 (Fig. 4e,f,h,i). In fact, PAMP stimulation seems to induce MARCH2 expression (also it seems to localize in distinct puncta, which is not happening upon overexpression of MARCH2 alone (Suppl 13C)). However, in these experiments one cannot rule out the effect of MG132. Does MARCH2 expression increases in the absence of MG132? If so, this would suggest a negative feedback mechanism and has to be further investigated/discussed.
- MARCH2 is a membrane-associated protein. Which membranes? Where does interaction with NEMO take place? Is MARCH2 recruited to the signaling complex?
- The discussion section is often repetitive (eg lines 423-426 and 443-448; 451-460) and largely descriptive. It needs a better reflection on the obtained results placing the findings in a general context. Although the authors suggest potential therapeutic implications, these remain unclear. Is there a specific MARCH2 inhibitor? Are there human patients with MARCH2 mutations?

Minor concerns:
- The authors switch a lot between several cell lines (BMDMs, PM, RAW, HEK, HeLa, A549) and different viruses plus other PAMPs, which makes it sometimes hard to follow. I would suggest to better introduce why sometimes other cellular models/cell lines or viruses are used
- Supplementary figure 1 feels unnecessary and a poor justification for selecting 'MARCH2 as a primary target' (as several other family members have the same effect)
- Lower exposure of K48 ubiquitin blots in several figures is desirable
- Figure 4e,f: why all of a sudden these experiments are done with NDF virus, while all previous experiments were done with VSV, HSV or PR-8? Yet PR-8 is used again in 5c and VSV in figure 6.
- Supplementary figure 9b-d requires a MARCH2 pull-down western blot and preferably IKKalpha-beta-gamma pull-down on the same blot for a proper comparison. It looks like IKKalpha is also pulled-down by MARCH2.

Responses to the Reviewers' comments (comments from reviewers in **black,** responses to the reviewers in **blue,** changes in the text are marked in **red.)**

Reviewer #1 (Comments for the Author):

Comment)

In the present manuscript, Chathuranga et al identify the E3 ligase MARCH2 as a negative regulator of innate immune responses against viral and bacterial infection. Mechanistically, they show that MARCH2 directly associates with NEMO/IKKg, the essential regulator of canonical IkB kinase/NF-kB signaling. MARCH2 conjugates K48-linked ubiquitin chains to NEMO, which triggers its proteasomal degradation post-induction. Thus, the authors suggest that MARCH2 restricts anti-viral and anti-bacterial responses by limiting the amounts of NEMO and thus canonical NF-kB signaling upon innate immune stimulation.

The authors present compelling evidence that MARCH2 acts as a negative regulator of innate immune responses. These include the data on MARCH2-deficient mice as well as a different cellular models using knock-out, knock-down, overexpression and reconstitution. In addition, a role of MARCH2 for NEMO ubiquitination and degradation is documented by overexpression, knock-out/knock-downs, mutagenesis, mapping of the ubiquitin attachment, providing clear evidence that MARCH2 can act as a NEMO ubiquitin ligase.

Response)

We would like to thank the reviewer for evaluating our work as well as for giving an opportunity to improve the study. We have thoughtfully revised the manuscript and addressed the reviewer's comments below.

Comment)

My major concern is that both parts are not well-connected and that there is no clear functional evidence that MARCH2 antagonizes innate immunity via NEMO degradation. Previous studies showed that MARCH2 localizes largely to endosomal vesicles and the plasma membrane and anti-viral responses may still be connected for instance to the endosomal sorting machinery. To confirm that MARCH2 antagonizes innate immunity via NEMO binding/ubiquitination, I think the following points need be addressed:

Response)

We thank the reviewer for the insightful comment. Previous reports have identified the subcellular localization of MARCH2 in Endoplasmic Reticulum (ER) and lysosomal, endosomal vesicles, where MARCH2 is embedded into membranes via two transmembrane domains[1, 2]. To address reviewer`s concern, we employed ER marker or lyso-tracker and verified the subcellular localization of MARCH2 upon PAMP stimulation by confocal microscopy (Appendix Figure S6 and S7). Interestingly, we could find that a certain amount of MARCH2 localizes in ER or lysosome while a substantial amount of MARCH2 protein retains in the cytoplasm following virus infection (NDV) or TLR2 agonist (Zymosan) without translocating to the membranes at late times of virus infection or TLR2 agonist stimulation.

Furthermore, we performed fractionation assay (membrane and cytosolic fraction) following pathogen infection to examine whether MARCH2 localizes in cytoplasm, where it can interact with NEMO. At the late stage after NDV infection, MARCH2 protein was also a bit observed in the membrane, although it abundantly located in the cytoplasm (Expanded View Figure 3G). Next, immunoprecipitation assay with cytoplasmic fraction showed that the interaction of MARCH2 with NEMO occurs in cytoplasm following NDV infection (Expanded View Figure 3H). Taken together, our findings suggest that MARCH2 localize in cytoplasm and directly interacts with NEMO to regulate innate immunity in response to pathogen infection. Revised manuscript explanation in Line 276-286.

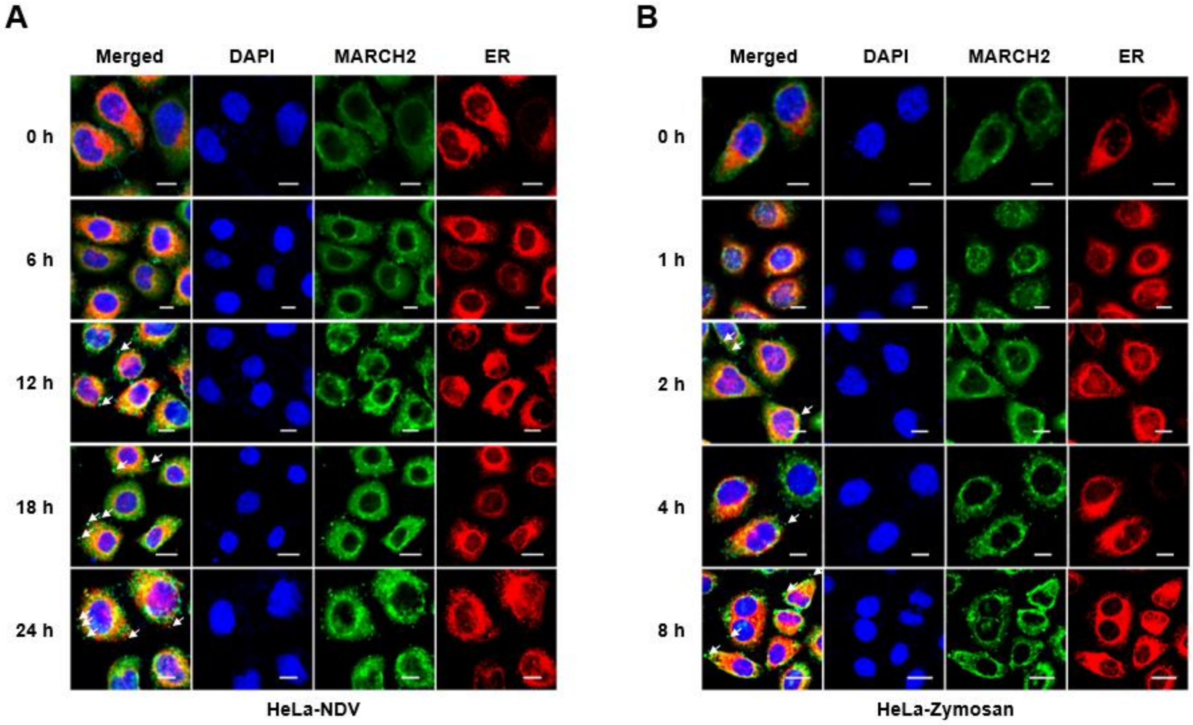

Appendix Figure S6A and S6B

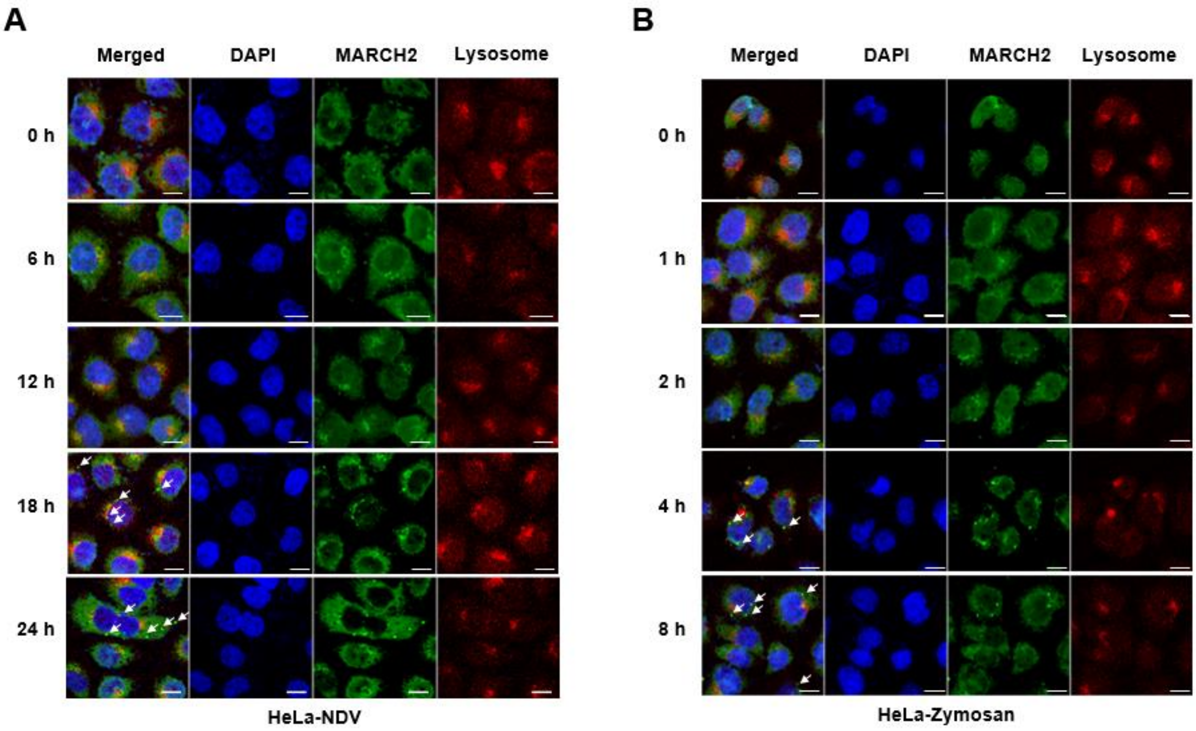

Appendix Figure S7A and S7B

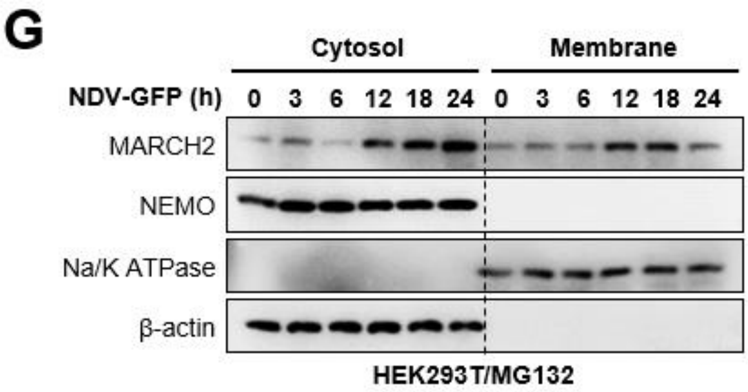

Expanded View Figure 3G

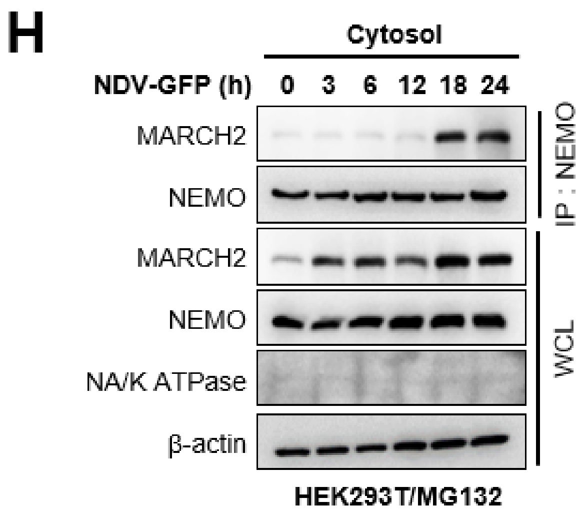

Expanded View Figure 3H

Supporting references:

1. Bartee, Eric, et al. Downregulation of major histocompatibility complex class I by human ubiquitin ligases related to viral immune evasion proteins. Journal of virology 78.3 (2004): 1109-1120.

2. Nakamura N, Fukuda H, Kato A, Hirose S (2005) MARCH-II is a syntaxin-6–binding protein involved in endosomal trafficking. Molecular biology of the cell 16: 1696-1710

Comment)

1) It is shown that mutation of the ubiquitin acceptor site K326 in NEMO is abolishing the negative regulatory function of MARCH2 on anti-viral responses in HEK293 cells, but there are no data showing that VSV-GFP infection is actually decreasing expression of NEMO wt, but not NEMO K326R. I think such data need to be included to lend support to the model.

Response)

We thank reviewer for raising the point. As we have shown in the Figure 7A, NEMO-WT expression level was drastically reduce while NEMO-K326R not when co-transfected with MARCH2. Therefore, it clearly emphasizes that MARCH2 could not lead to proteasomal degradation of K32R mutant highlighting its importance of recruiting ubiquitin from MARCH2.

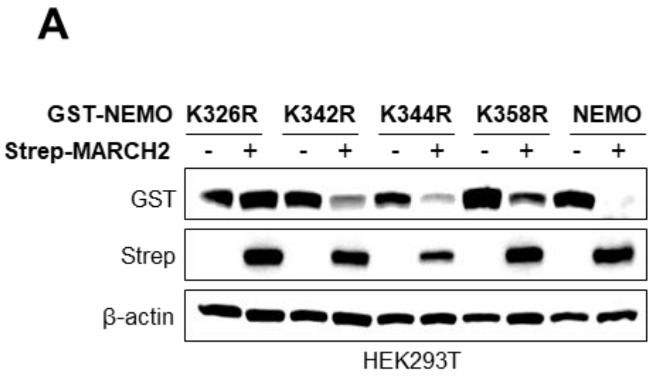

Figure 7A

Furthermore, as we have addressed in the reviewers' below comment, endogenous NEMO expression was dramatically reduced in the wild type HEK293T cells or BMDM cells but not in the MARCH2 knock out HEK293T cells or BMDM cells upon viral or bacterial infection. These results suggest that endogenous MARCH2 could control endogenous NEMO stability via degradation upon pathogen infection to regulate immune homeostasis.

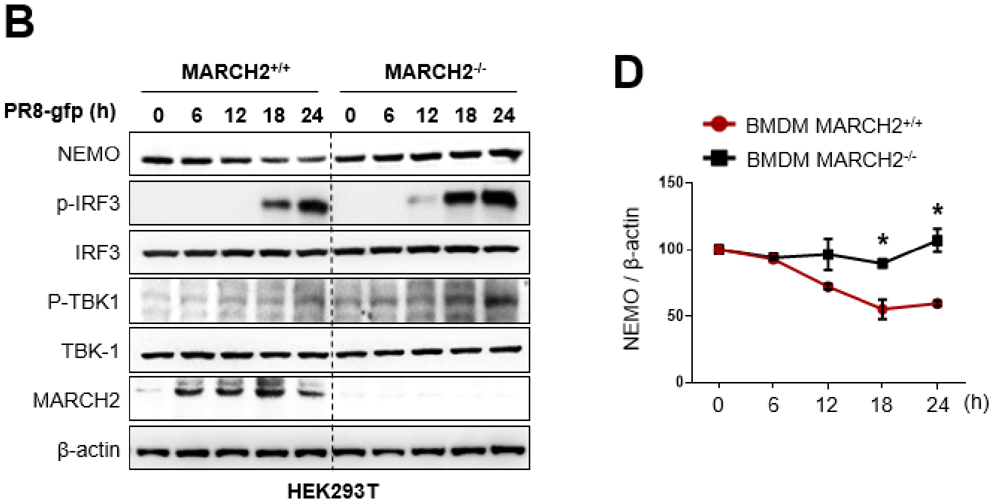

Figure 5B and Expanded View Figure 4D

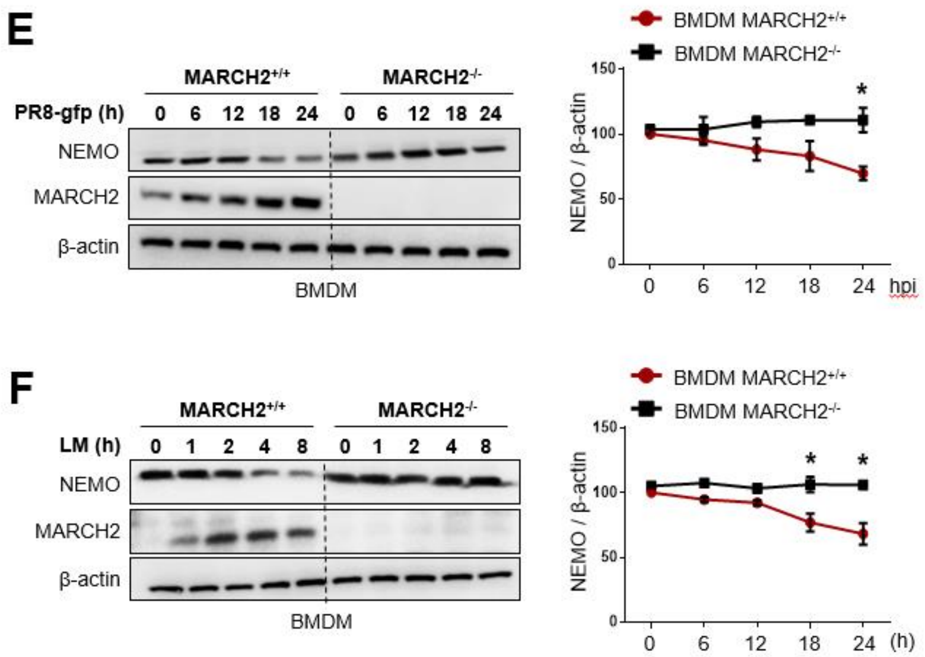

Expanded View Figure 4E-F

To address the point raised by the reviewer, we transfected NEMO-WT and K326R expression plasmids to HEK293T cells and infected with different doses of PR8-GFP virus at 24 hours post transfection. Then cells were harvested at indicated time points following infection.

We make every effort to address the reviewer comment at our level best. However, as following data, we could observe some degradation of overexpressed NEMO-WT by even upon high virus MOI infection. Similarly, overexpressed NEMO mutant was not degraded upon virus infection. Based on our results, we believe that endogenous MARCH2 could not sufficient to degrade overexpressed NEMO via K48-linked poly-ubiquitination. Therefore, we decided to include this information in the reviewer comment section.

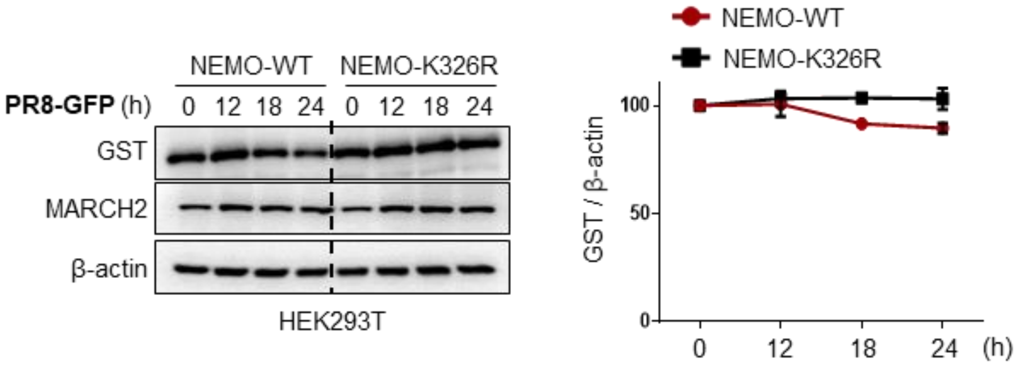

Additionally, our phenotype results observed in the Figure 7D-G in the revised manuscript are also line with the above results. Over expression of NEMO-WT or NEMO-K326R alone did not show any significant difference in the virus replication or cytokine secretion, highlighting that endogenous MARCH2 not sufficient enough to degrade overexpressed NEMO.

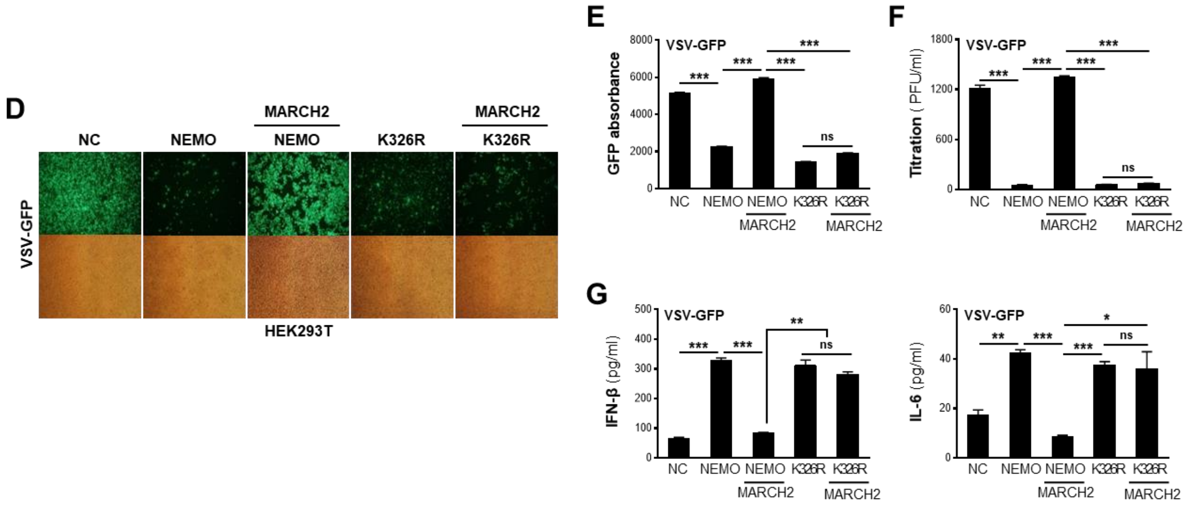

Figure 7D-G

Comment)

2) With the exception in Figure 5c, NEMO degradation is only observed after MARCH2 overexpression. To confirm their mechanistic claim, the authors need to show that NEMO stability is increased in primary MARCH2-/- cells (e.g. BMDM) after viral infection (e.g. VSV) and/or upon PAMP recognition (e.g. poly(I:C), LPS, Lm). This is important because at least I am not aware of publications demonstrating that NEMO is indeed prone to proteasomal degradation post-induction.

Response)

We would be thankful to reviewer for the insightful comment. As we have shown in Fig. 5C of the original manuscript, endogenous NEMO protein expression level was decreased in MARCH2+/+ HEK293T cells compared to MARCH2-/- HEK293T upon Influenza virus (PR8-GFP) infection in the absence of MG132. According to the reviewer`s suggestion, we performed additional experiments with primary macrophages (BMDMs) in the absence of MG132 following the same protocol with PR8-GFP or *Listeria monocytogenes* infection. Then, we confirmed that endogenous NEMO was degraded in wild-type BMDMs at late time points while it was stable in MARCH2 knock-out BMDMs, suggesting that MARCH2 controls NEMO stability via degradation to subvert host innate immune responses (Expanded View Figure 4E and F). Furthermore, Xing *et al* have previously demonstrated that endogenous NEMO degradation upon influenza virus infection in alveolar macrophage cells and mechanistically they demonstrate as direct binding of TRIM29 with NEMO induces its ubiquitination and proteolytic degradation. Revised manuscript explanation in Line 303-307 and 491-496.

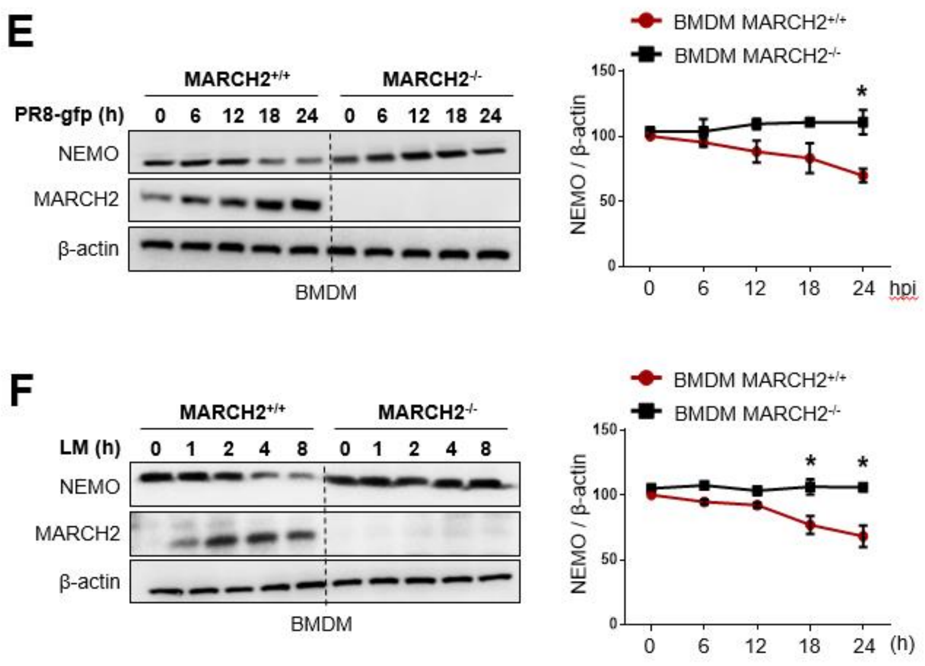

Expanded View Figure 4E and F

Supporting reference:

1. Xing J, Weng L, Yuan B, Wang Z, Jia L, Jin R, Lu H, Li XC, Liu Y-J, Zhang Z. 2016. Identification of a role for TRIM29 in the control of innate immunity in the respiratory tract.

Nature immunology 17:1373-1380.

Comment)

3) While loss of MARCH2 decreases IKK/NF-kB signaling, MARCH2 seems to have a stronger effect in counteracting TBK1 and IRF3 phosphorylation. Are the authors claiming that NEMO degradation is also enhancing TBK1-IRF3 signaling? If so, can the authors show that TBK1-IRF3 is controlled by NEMO in HEK293 cells? I think it is very likely that MARCH2 may have additional targets to control innate activation of TBK1/IRF pathway. It may go beyond the scope to identify these targets, but at least it needs to be discussed.

Response)

We thank the reviewer for the comment. It has been previously reported that NEMO is important to activate TBK1-IRF3 signaling axis following virus infection. Fang *et al.* demonstrated in two separate studies that loss of NEMO impaired the TBK1-IRF3 activation upon RNA and DNA virus infection. As we have shown in Figure 5B, Expanded View Figure 4D, Appendix Figure S1A and S2E, loss of MARCH2 increased TBK1 and IRF3 phosphorylation upon virus infection in HEK293T cells, BMDMs and Raw264.7 cells, respectively. These results strongly suggest that MARCH2 has a critical role in regulation of type I IFN signaling pathway via NEMO degradation. As the reviewer suggested, there could be some additional target of MARCH2 to regulate the innate immune system. However, we could not find any specific molecule that regulates TBK1-IRF3 signaling axis by a comprehensive analysis of Strep-MARCH2 pull-down and mass spectrometry results. Thus, with present knowledge, we believe that NEMO and MARCH2 interaction play a possible, an essential role in regulating the homeostasis of the innate immune system.

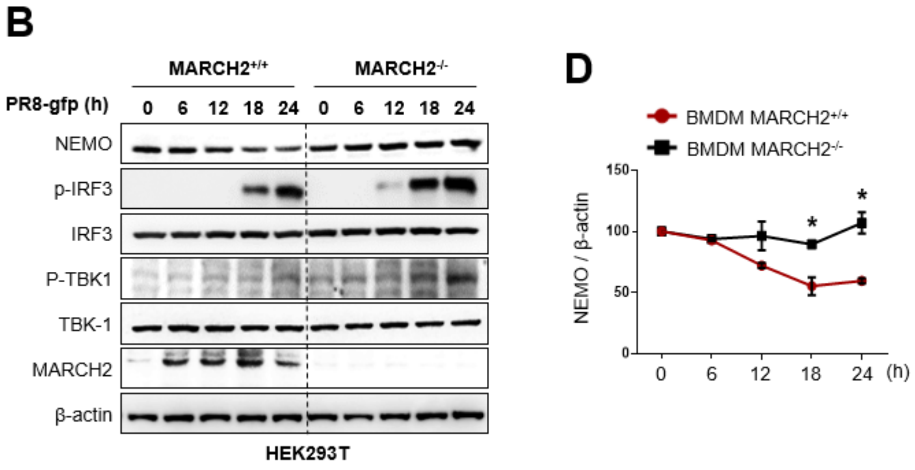

Figure 5B and Expanded View Figure 4D

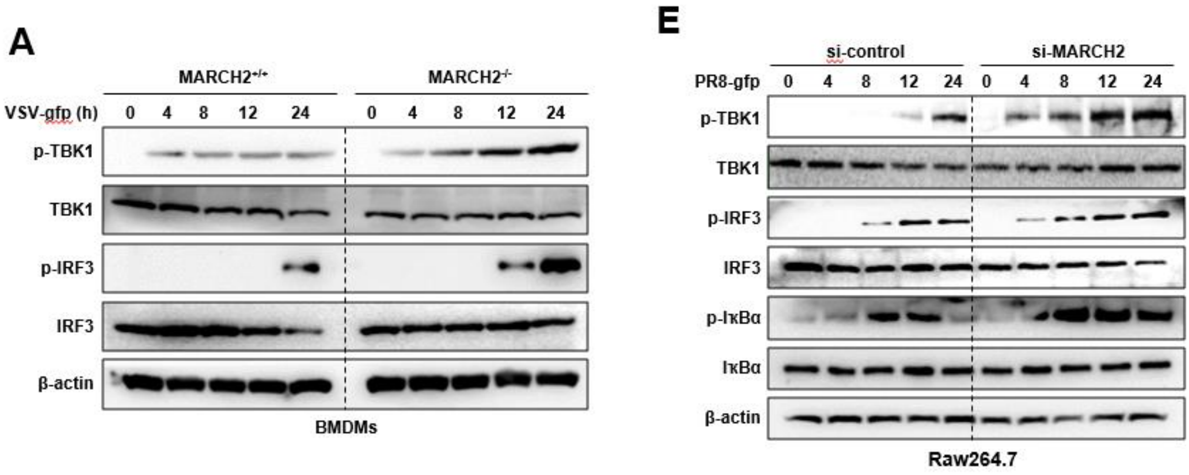

Appendix Figure S1A and S2E

Supporting references:

1. Fang R, Jiang Q, Zhou X, Wang C, Guan Y, Tao J, Xi J, Feng J-M, Jiang Z. 2017. MAVS activates TBK1 and IKKε through TRAFs in NEMO dependent and independent manner. PLoS pathogens 13:e1006720.

2. Fang R, Wang C, Jiang Q, Lv M, Gao P, Yu X, Mu P, Zhang R, Bi S, Feng J-M. 2017. NEMO–IKKβ are essential for IRF3 and NF-κB activation in the cGAS–STING pathway. The Journal of Immunology 199:3222-3233.

Comment)

Specific points:

The description of the generation and validation of March2-/- mice is not acceptable. The description in Material and Methods is not sufficient to understand the KO strategy. What were the sgRNAs, what were expected genomic alterations and how did they verify these by PCR? Genotyping in Supplementary Figure 2 is not understandable and not helpful in its present form. A scheme showing targeting and verification should be included. Further, apparently the author do not have an antibody for detection of murine MARCH2. To confirm the expected effects, at least a decrease or truncation of MARCH2 transcripts should be demonstrated on mRNA level.

Response)

We thank the reviewer for raising the point. As the reviewer`s instruction, we have included schematic diagrams that show a targeting position and sequence of gRNA and the edited

sequence of MARCH2 gene and amino acid (Expanded View Figure 1A and B). Other than the agarose gel electrophoresis analysis used for genotyping the mice (Expanded View Figure 1C-F), we have verified knock-out of MARCH2 through western blotting with a MARCH2 specific antibody (ab123136) using wild-type and MARCH2 knock-out BMDMs infected with virus (Expanded View Figure 1G). A detailed explanation of the generation and validation of MARCH2 KO mice were described in Materials and Methods. Revised manuscript explanation in Line 550-561. We believe that the revised manuscript would be more understandable than previous.

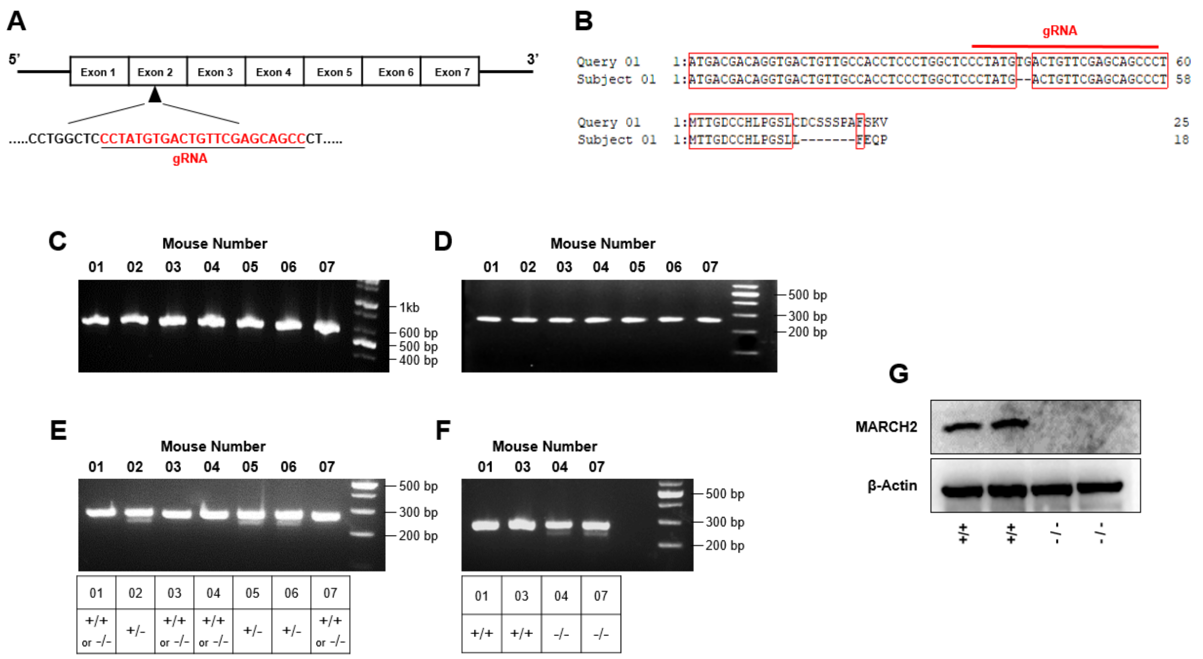

Expanded View Figure 1

Comment)

Figure 4e, f, h, i: MARCH2 expression is induced by the stimulation. How is MARCH2 regulated? Is March2 mRNA induced or is this simply post-translational stabilization due to MG132 treatment?

Response)

We thank the reviewer for the critical comment. First, we could identify that endogenous MARCH2 expression maintains at a low level, and is increased upon virus or bacterial infection without the post-translational stabilization by MG132 treatment (Figure 5B, Expanded View Figure 4D-F).

Next, to address the reviewer's comment with the additional evidence, we have assessed the

mRNA induction level of MARCH2 gene in response to virus infection (PR8-GFP, NDV-GFP, HSV-GFP), ligand stimulation (Poly(I:C)), or bacterial infection (Salmonella, Listeria) in RAW264.7 cells as well as BMDMs isolated from wild-type mice (Expanded View Figure 3E and F). As results, the qPCR showed the mRNA induction of MARCH2 gene was significantly induced by those stimulations without MG132 treatment, in line with the increasing expression of MARCH2 shown in Figure 5B, Expanded View Figure 4E and F. However, as a specific mechanism driving MARCH2 expression remains unknown, further investigation (e.g. Mass Spectrometry to detect any possible PTM of MARCH2 against infection or host factor screening affecting MARCH2 induction etc.) would be valuable as described by the reviewer. Revised manuscript explanation in Line 275-279.

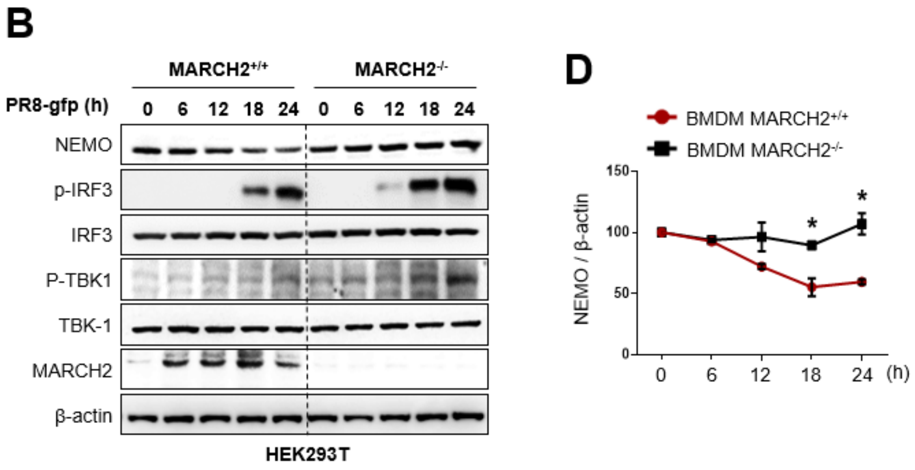

Figure 5B and Expanded View Figure 4D

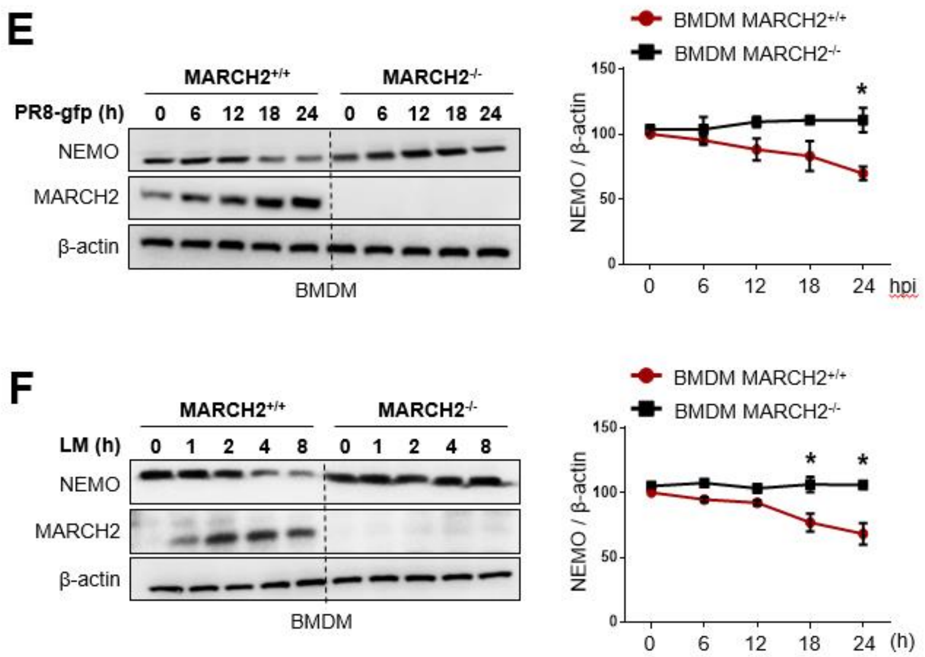

Expanded View Figure 4E-F

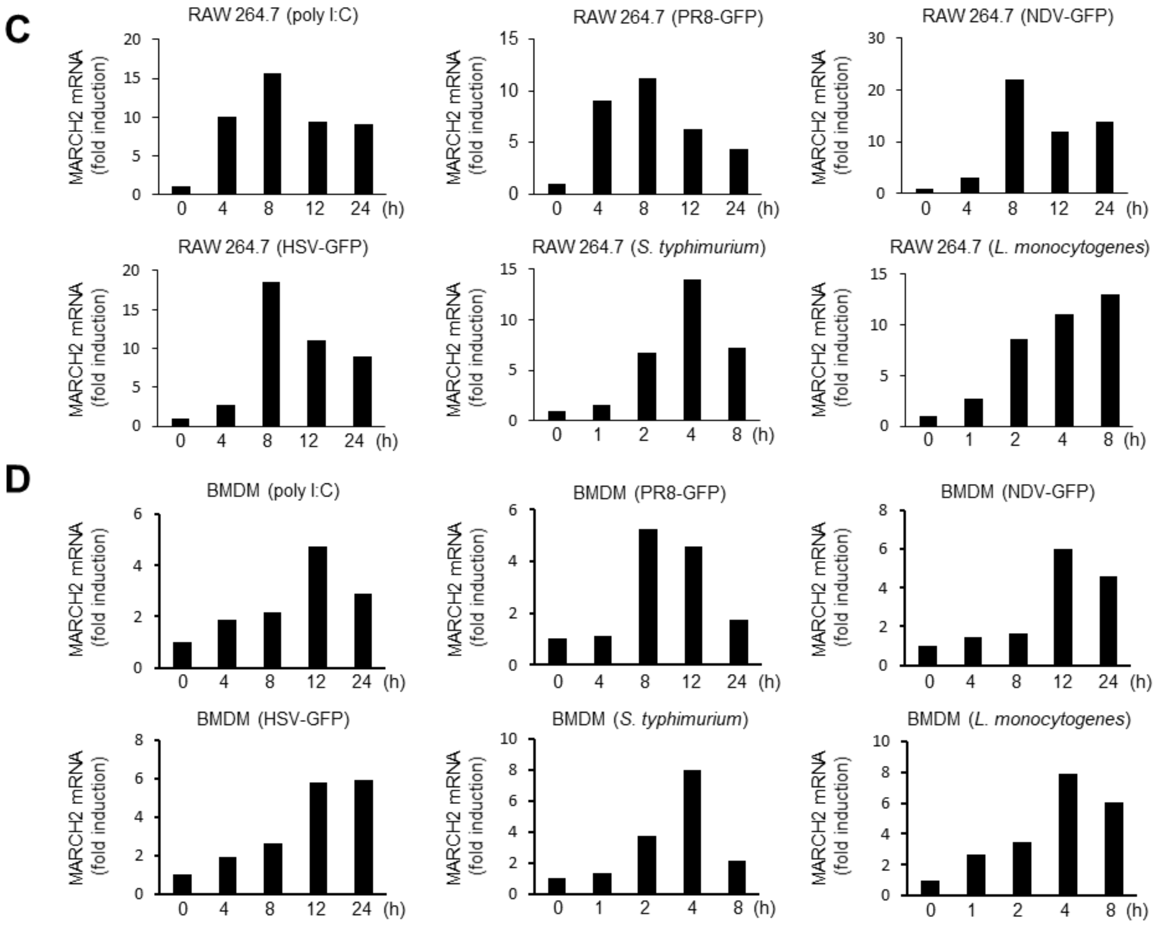

Expanded View Figure 3C and D

Comment)

Figure 7 and S9: Does MARCH2 bind to NEMO when bound to IKKs? MARCH2 binding to the CC1 domain of NEMO could also lead to dissociation of NEMO from IKKs.

Response)

We would like to thank the reviewer for raising the rational concern. It has been reported that NEMO CC1 domain is from 100 amino acid to 194 amino acid and 44-111 residue of NEMO is interacting with IKKs. As our results showed, the endogenous interaction of NEMO with MARCH2 did not affect its interaction with IKKα (Figure 4E, F, H, and I and Appendix Figure S5A and B). Moreover, we have confirmed there is no change in the interaction of NEMO with IKKα or IKKβ regardless of MARCH2 expression in the overexpression system (Expanded View Figure 5C and D). These results show that MARCH2 is not implicated in the dissociation of NEMO with IKKα or IKKβ. It is most likely that MARCH2 regulates NEMO via its K-48 linked ubiquitination-mediated degradation, not physical disruption. Furthermore, it was shown that MARCH2 cannot interact with IKKα or IKKβ in absence of NEMO, suggesting that MARCH2 interacts with IKK complex via NEMO to negatively regulate its signaling. Revised manuscript explanation in Line 369-374.

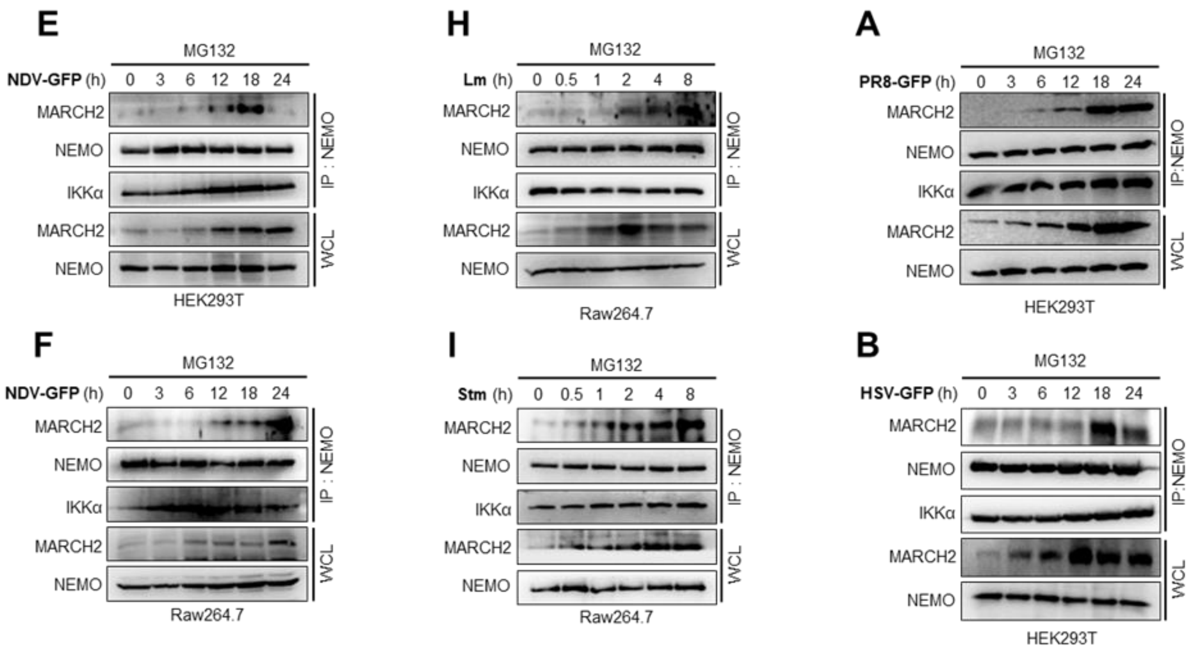

Figure 4E, F, H, I and Appendix Figure S5 A and B

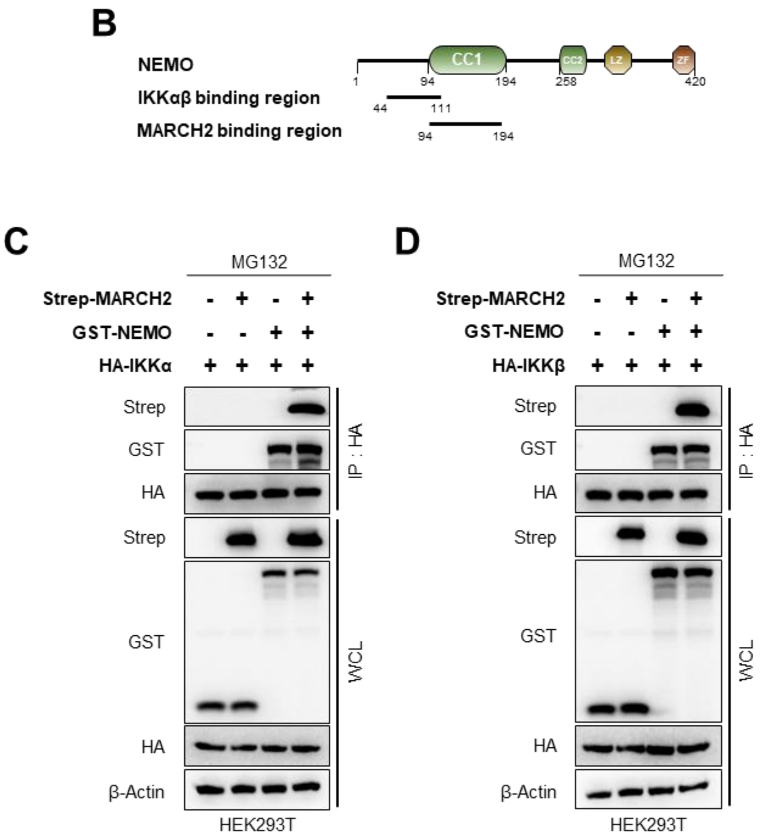

Expanded View Figure 5B-D

Supporting reference:

1. Israël A. 2010. The IKK complex, a central regulator of NF-κB activation. Cold Spring Harbor perspectives in biology 2:a000158.

2. Rushe M, Silvian L, Bixler S, Chen LL, Cheung A, Bowes S, Cuervo H, Berkowitz S, Zheng T, Guckian K. 2008. Structure of a NEMO/IKK-associating domain reveals architecture of the interaction site. Structure 16:798-808.

Comment)

Minor concern:

Are the Western Blots with a dashed line (e.g. 5c, S3a, c, S4e, S5h etc.) from different gels? If so, the Blots should be separated.

Response)

In figure 5c, S3a, c, S4e, S5h (Figure 5B, Appendix Figure S1A, C, S2E, S3H, respectively in the revised manuscript), all blots are from the same gels. We have given the uncropped full images in source data file.

Comment)

Additional comment:

I think the manuscript can be shortened and streamlined. For example, biochemical studies showing MARCH2-NEMO interaction and regulation are dispersed between Figures 5-8 and the studies can be better combined and shortened. Also, appearance of figures and the description in the text is not always aligned and this should be corrected.

Response)

As reviewer suggested, Figure 5 and 6 were combined, now our manuscript have 7 main figures. We re-arranged the figures to make it aligned with the floor of manuscript text. We appreciate the reviewer for a valuable advice, which made our manuscript more intensive and improved.

<u>Reviewer #2 (Comments for the Author):</u>

Comment)

In this paper, Chathuranga et al. identified the ubiquitin ligase MARCH2 as a negative regulator of IFN and NF-□B signaling in response to viral and bacterial infection (or stimulation with PAMPs) in vitro and in vivo. The authors show that MARCH2 deficiency protects mice from viral or bacterial infection, accompanied by increased cytokine levels. Mechanistically, the authors report that MARCH2 catalyzes K48 ubiquitination of NEMO, leading to its proteasomal degradation. The experiments are clear and well controlled. Although these findings are novel and potentially interesting, there are several shortcomings that need to be addressed:

Response)

We would like to thank the reviewer for evaluating our work as well as for giving an opportunity to improve the study. We thoughtfully revised the manuscript and addressed the reviewer's suggestions below.

Major concerns:

Comment)

The authors suggest in the discussion that MARCH2-deficient mice could potentially suffer from autoimmunity or chronic inflammation. Yet control mice in their in vivo experiments seem to have cytokine levels similar to wild type mice. This is highly surprising given the phenotype of mice lacking other negative regulators of NF-□B, such as A20. It would be important to investigate/document in more detail whether MARCH2 deficiency causes any spontaneous autoimmune phenotype**.**

Response)

We thank the reviewer for raising the point as well as totally agree with the comment. However, in our experimental data, we did not observe any external abnormality in the MARCH2-KO mice compared to MARCH2-WT mice in resting state. Therefore, it needs to be investigated more to hypothesize MARCH2 is involved in autoimmune disorders and that will be an interesting study.

MARCH2 is a negative regulator that is induced by stimulation. As suggested by the reviewer

below (Comment 03), we have assessed the mRNA induction level of MARCH2 in response to the virus/bacterial infection. The qPCR data showed that MARCH2 is induced a few hours after infection (EV 3E and F). Moreover, in Figure 5B, EV4D-F, the expression level of endogenous MARCH2 is low but increased with viral or bacterial infection. These features of MARCH2 suggest that MARCH2 is employed to maintain homeostasis when it comes to immune activation or stimulation. For this reason, we agree that our discussion about the autoimmune disorder is not fully supported by present data and in the revised manuscript we have removed the relevant text from the discussion. (Original manuscript Line 439-441)

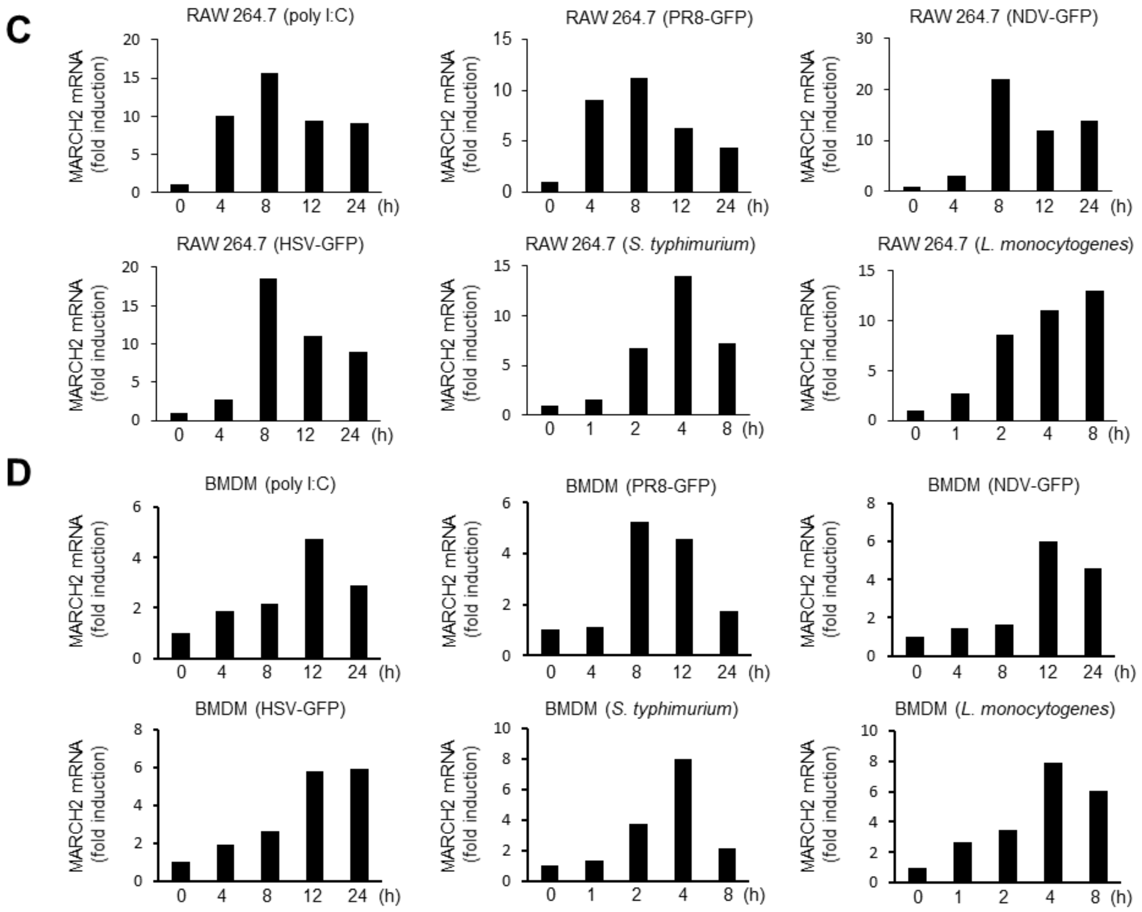

Expanded View Figure 3C and D

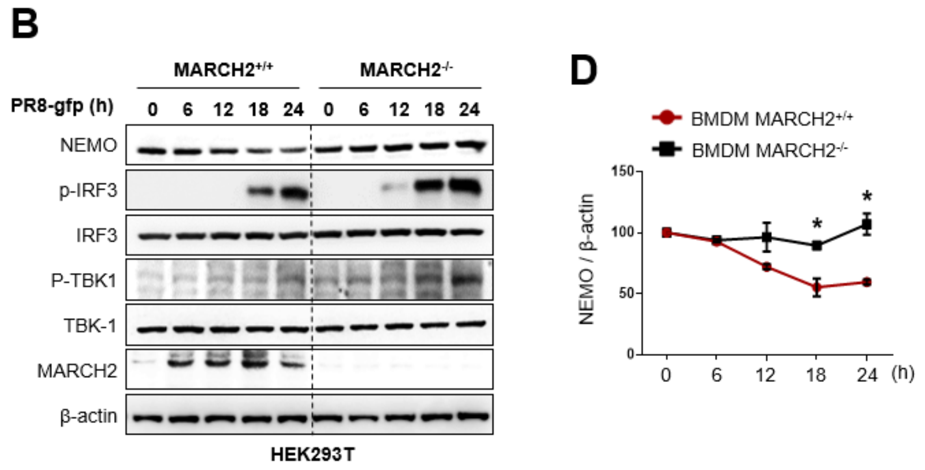

Figure 5B and Expanded View Figure 4D

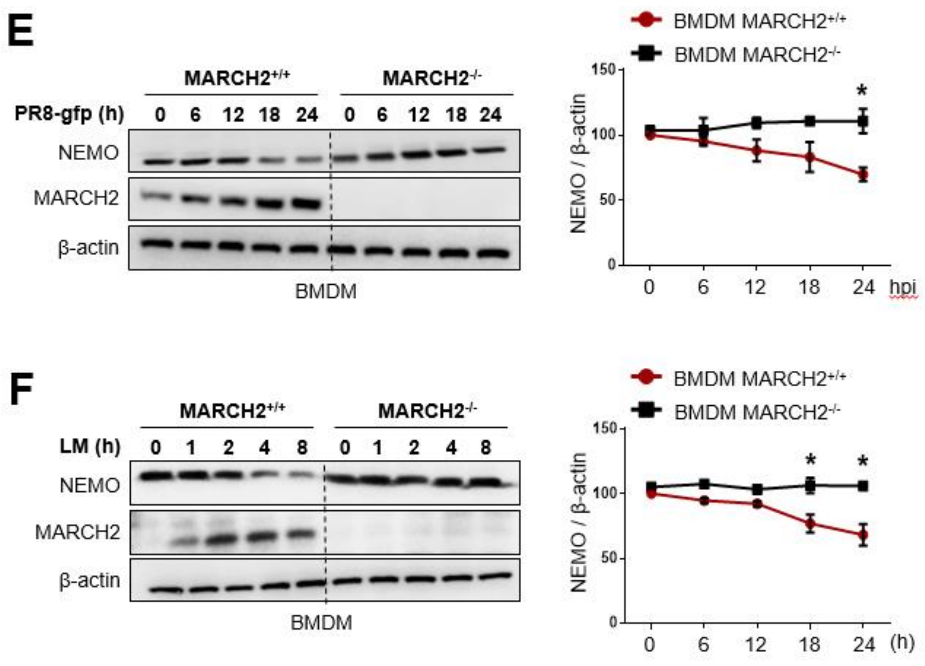

Expanded View Figure 4E-F

Comment)

In relation to the above comment (normal cytokine levels/absence of inflammation in control knockout group): does MARCH2 regulate only PAMP-initiated signaling? What about DAMPs (eg IL-1 stimulation)?

Response)

We thank the reviewer for the insightful comments. We assessed the effect of MARCH2 on NEMO in response to the DAMP-related signaling. Given that DAMP-initiated signaling

shares some molecules in the pathway with PAMP signaling[1], we expected that MARCH2 might be implicated in control of cytokine secretion.

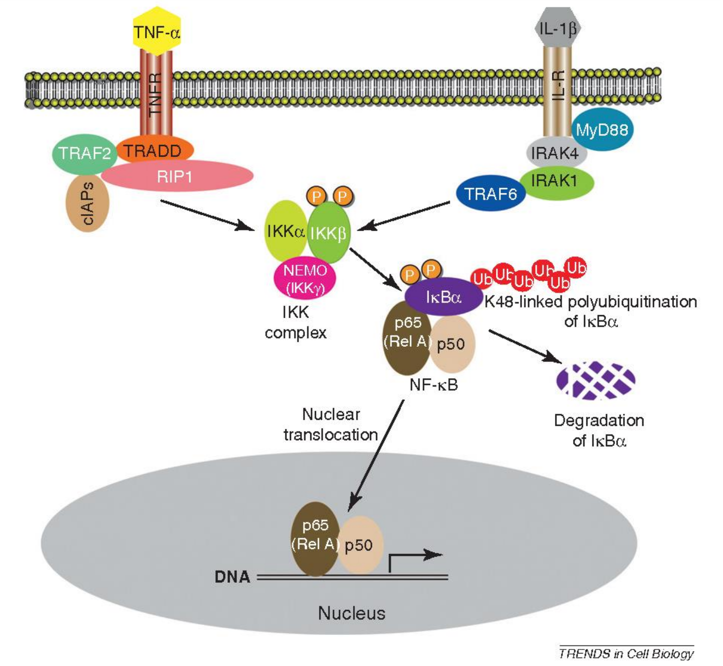

Iwai, Kazuhiro. "Diverse ubiquitin signaling in NF-κB activation."
Trends in cell biology 22 7 (2012): 355-64.

To address this, we have isolated BMDMs from WT and MARCH2-/- mice and treated the cells with mouse TNF-α or mouse IL-1β. We collected the cell pellets and supernatant at the indicated time points and measured the mRNA induction level and the secretion amount of the IL-6, respectively. As a result, qPCR and ELISA showed the deficiency of MARCH2 caused increased mRNA induction of IL-6 as well as the secretion level, which implies MARCH2 could also be involved in the DAMP-initiated signaling pathway. However, we do not insist that MARCH2 regulates NEMO in response to the DAMP stimulation at this stage, because it needs to be investigated more with additional experiments such as binding assay or ubiquitination assay of NEMO. For this reason, we are now conducting further studies about the role of MARCH2 in the pathway initiated with TNF-α or IL-1β stimulation, possible immune diseases and hope to report more detail afterward.

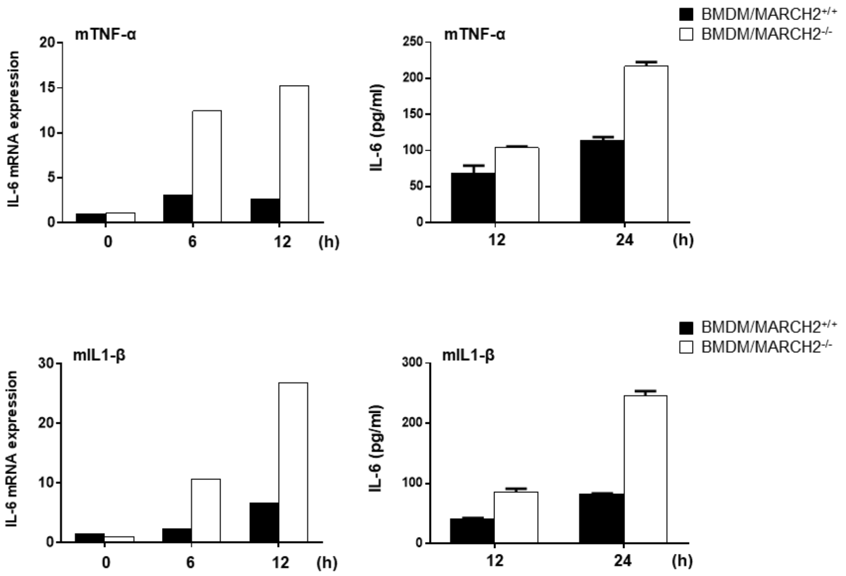

mRNA induction and secretion level of IL-6 in response to TNF-α or IL-1-β treatment in BMDMs.

Comment)

The authors mention that MARCH2 interacts with NEMO at the late stages of infection, which coincides with increased expression levels of MARCH2 (Fig. 4e,f,h,i). In fact, PAMP stimulation seems to induce MARCH2 expression (also it seems to localize in distinct puncta, which is not happening upon overexpression of MARCH2 alone (Suppl 13C)). However, in these experiments one cannot rule out the effect of MG132. Does MARCH2 expression increases in the absence of MG132? If so, this would suggest a negative feedback mechanism and has to be further investigated/discussed.

Response)

We thank the reviewer for the insightful comment. First, we could identify that the expression level of endogenous MARCH2 maintained at a low level. However, the expression level was increased against virus infection without the post-translational stabilization by MG132 treatment (Figure 5C, now 5B in the revised manuscript). Next, to address the reviewer's comment with the additional evidence, we have assessed the mRNA induction level of MARCH2 in response to virus infection (PR8-GFP, NDV-GFP, HSV-GFP), ligand stimulation(Poly(I:C)), or bacterial infection (Salmonella, Listeria) in RAW264.7 cells as well as in BMDMs isolated from wild type mice (Expanded View Figure 3E and F). As result, the qPCR showed the mRNA induction of MARCH2 gene was significantly induced by those stimulations without MG132 treatment, in line with the increased expression of MARCH2

shown in Figure 5B, Expanded View Figure 4E and F.

As the reviewer highlighted, there would be a negative feedback to transcribe MARCH2 gene at a recovery phase. However, it remains unknown which kinds of cellular factor triggers the MARCH2 expression. Moreover, specific molecular mechanisms driving post-translational modifications (e.g. phosphorylation) on MARCH2 to initiate or stop its catalytic function for NEMO ubiquitination is needed to be elucidated. Therefore, further investigation would be important to strengthen this hypothesis. Revised manuscript explanation in Line 275-278 and line 506-510.

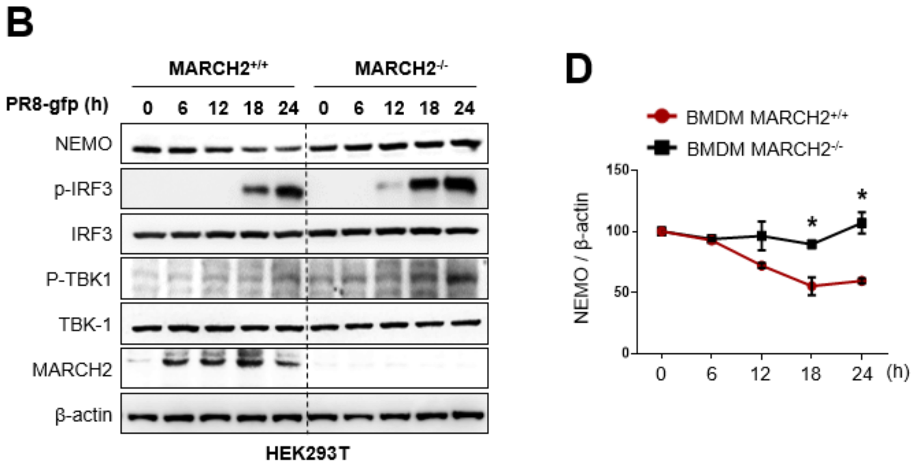

Figure 5B and Expanded View Figure 4D

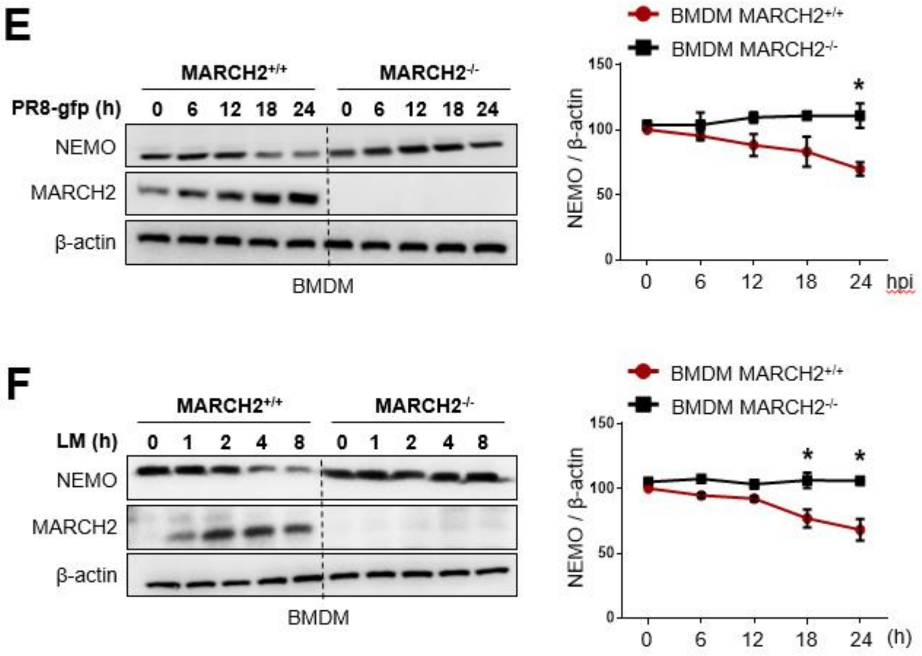

Expanded View Figure 4E-F

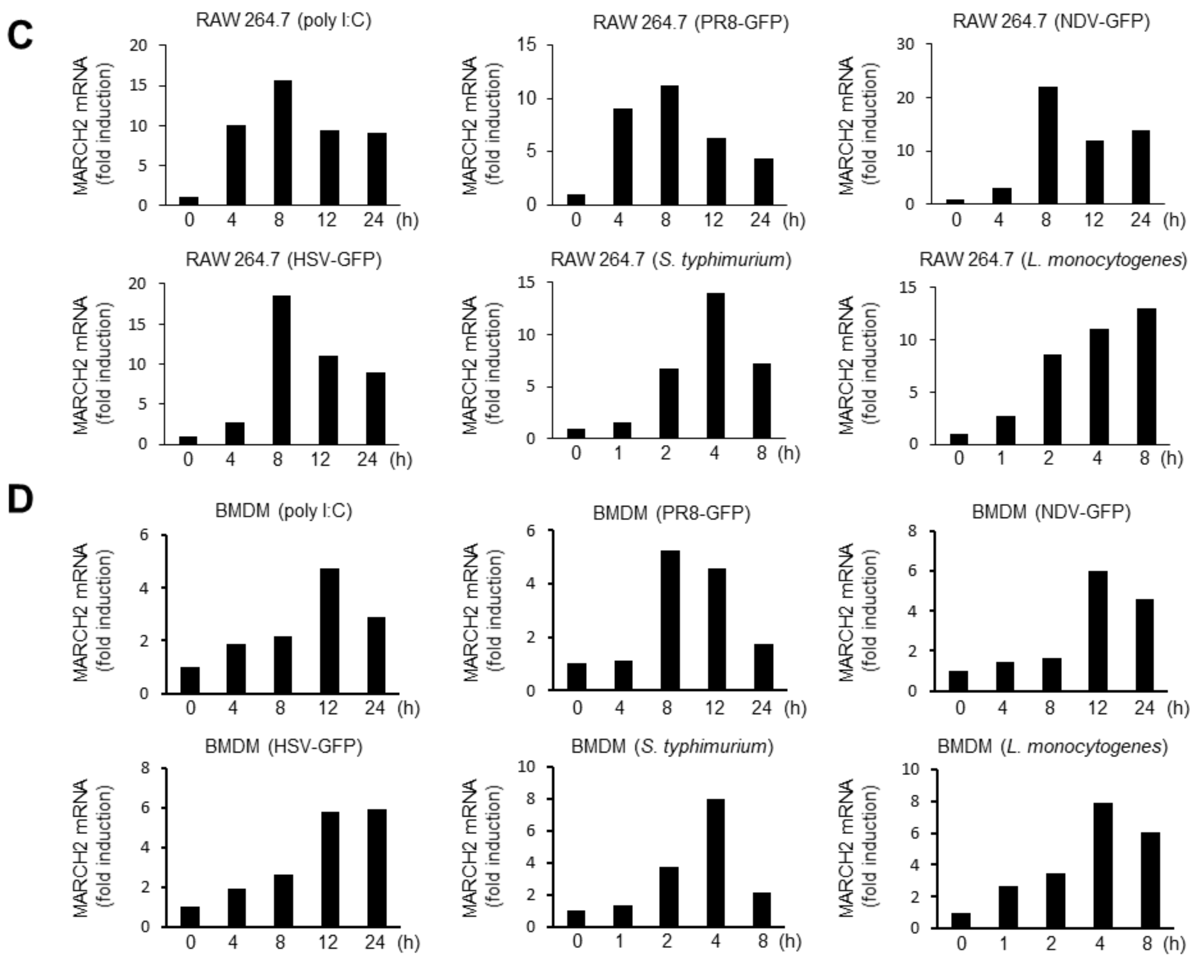

Expanded View Figure 3C and D

Comment)

MARCH2 is a membrane-associated protein. Which membranes? Where does interaction with NEMO take place? Is MARCH2 recruited to the signaling complex?

We thank the reviewer for the insightful comment. Previous reports have identified the subcellular localization of MARCH2 in Endoplasmic Reticulum (ER) and lysosomal, endosomal vesicles, where MARCH2 is embedded into membranes via two transmembrane domains[1, 2]. To address reviewer`s concern, we employed ER marker or lyso-tracker and verified the subcellular localization of MARCH2 upon PAMP stimulation by confocal microscopy (Appendix Figure S6 and S7). Interestingly, we could find that a certain amount of MARCH2 localizes in ER or lysosome while a substantial amount of MARCH2 protein retains in the cytoplasm following virus infection (NDV) or TLR2 agonist (Zymosan) without translocating to the membranes at late times of virus infection or TLR2 agonist stimulation. Furthermore, we performed fractionation assay (membrane and cytosolic fraction) following

pathogen infection to examine whether MARCH2 localizes in cytoplasm, where it can interact with NEMO. At the late stage after NDV infection, newly synthesized MARCH2 protein was also a bit observed in the membrane, although it abundantly located in the cytoplasm (Expanded View Figure 3G). Next, immunoprecipitation assay with cytoplasmic fraction showed that the interaction of MARCH2 with NEMO occurs in cytoplasm following NDV infection (Expanded View Figure 3H). Taken together, our findings suggest that MARCH2 localize in cytoplasm and directly interacts with NEMO to regulate innate immunity in response to pathogen infection. Revised manuscript explanation in Line 279-288.

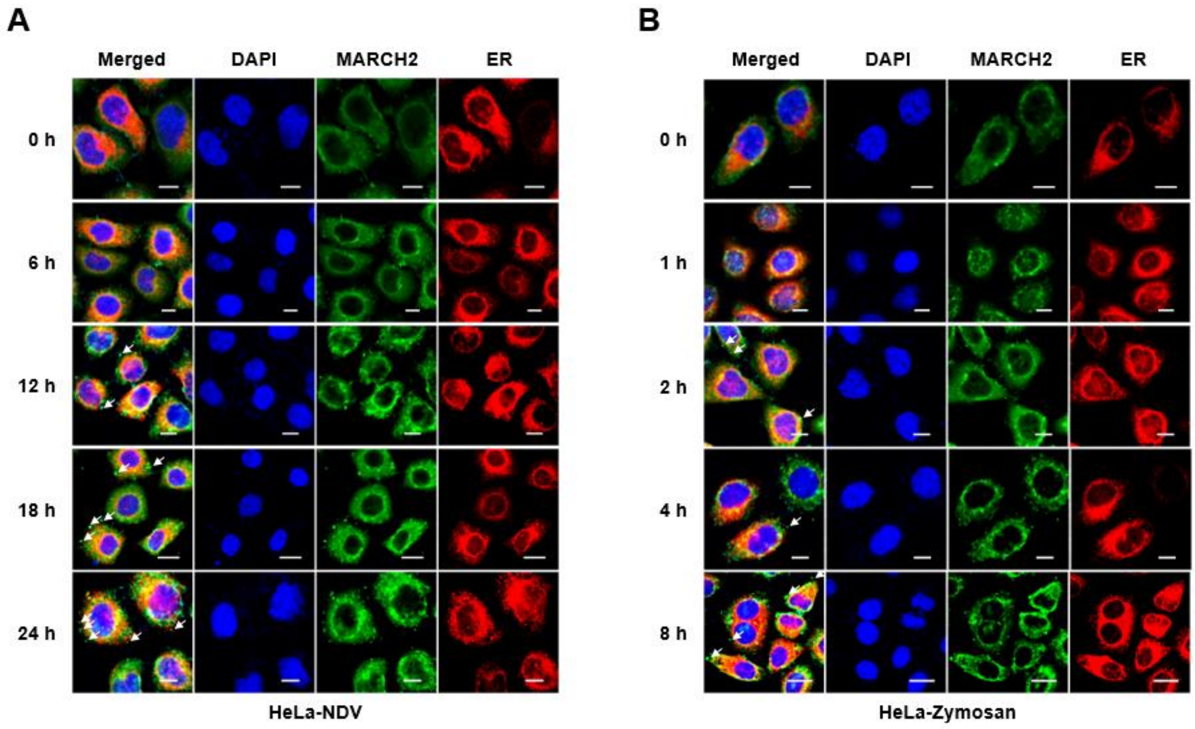

Appendix Figure S6A and S6B

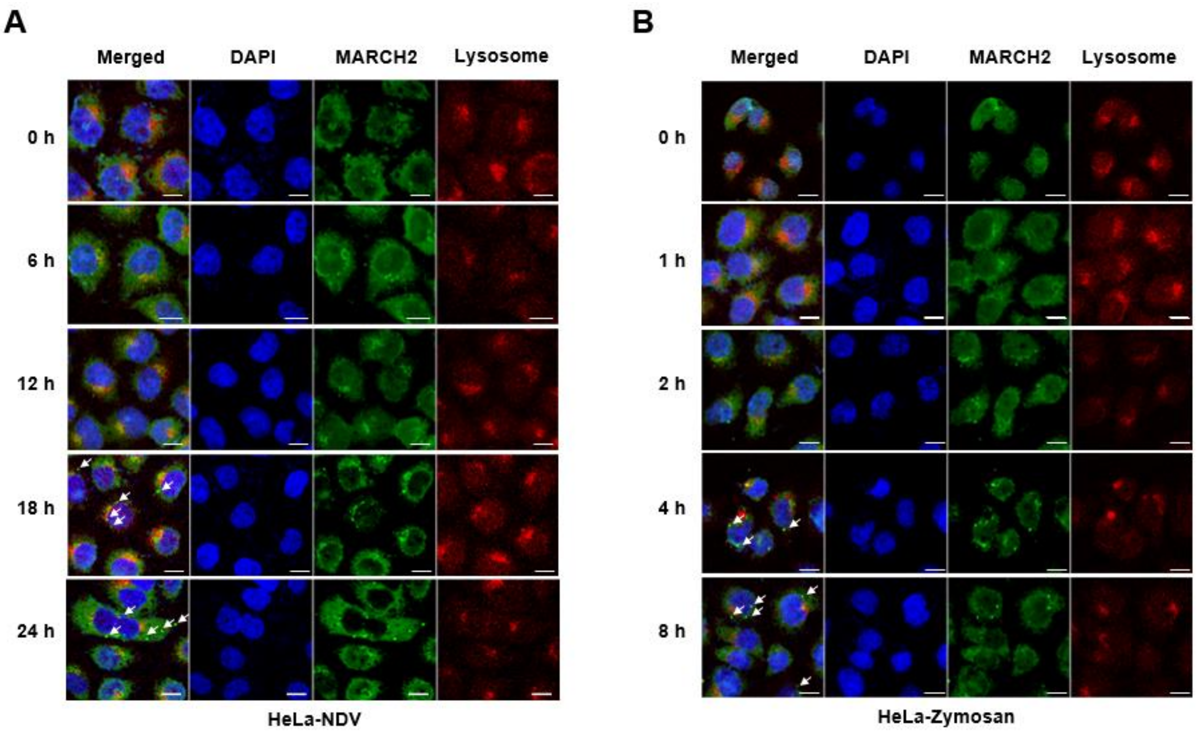

Appendix Figure S7A and S7B

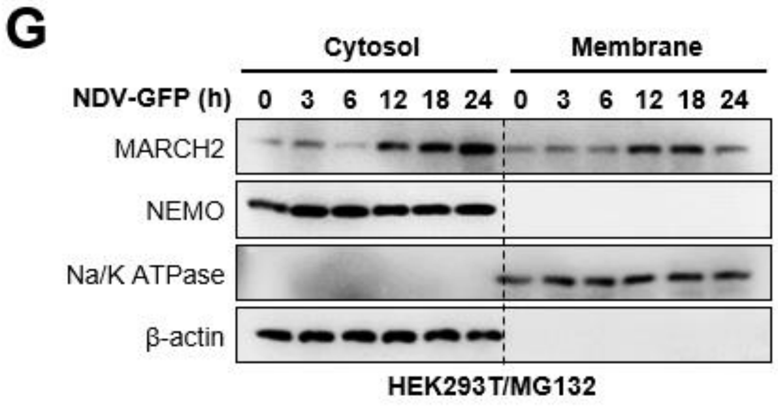

Expanded View Figure 3G

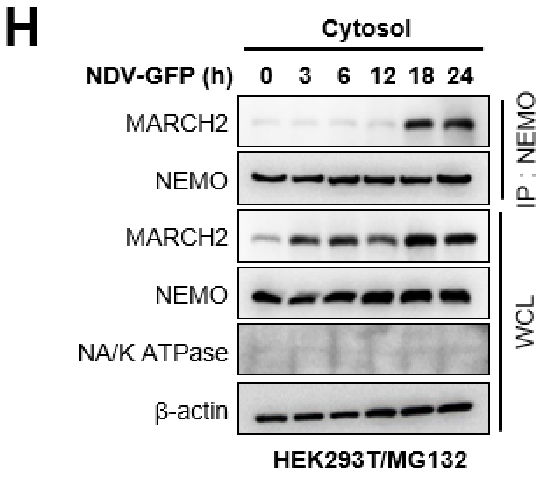

Expanded View Figure 3H

Supporting references:

1. Bartee, Eric, et al. Downregulation of major histocompatibility complex class I by human ubiquitin ligases related to viral immune evasion proteins. Journal of virology 78.3 (2004): 1109-1120.

2. Nakamura N, Fukuda H, Kato A, Hirose S (2005) MARCH-II is a syntaxin-6–binding protein involved in endosomal trafficking. Molecular biology of the cell 16: 1696-1710

Comment)

The discussion section is often repetitive (eg lines 423-426 and 443-448; 451-460) and largely descriptive. It needs a better reflection on the obtained results placing the findings in a general context. Although the authors suggest potential therapeutic implications, these remain unclear. Is there a specific MARCH2 inhibitor? Are there human patients with MARCH2 mutations?

Response)

We thank the reviewer for important advice. We removed the line 451-452, line 453-456, line 408, and line 467-470 of the original manuscript since it has some repetitive wording or additional information. Also, with the consensus of reviewer 01 comment, we have removed the line 439-441 of original manuscript.

Up to date, there is no reported case of a human patient in MARCH2 related genetic disorders (Clinvar database[1]). One of next our plans in the MARCH2 project, is to find out a chemical that can induce the MARCH2 protein level thus, reduce the inflammation via enhanced

NEMO degradation and it would be used as a potential anti-inflammatory drug. At the movement, we are screening a chemical library composed of 4000 chemicals to grab a potential MARCH2 activator.

Supporting reference:

1. Landrum MJ, Lee JM, Benson M, et al. ClinVar: improving access to variant interpretations and supporting evidence. Nucleic Acids Res. 2018;46(D1):D1062-D1067. doi:10.1093/nar/gkx1153

Minor concerns:

Comment)

The authors switch a lot between several cell lines (BMDMs, PM, RAW, HEK, HeLa, A549) and different viruses plus other PAMPs, which makes it sometimes hard to follow. I would suggest to better introduce why sometimes other cellular models/cell lines or viruses are used

Response)

We thank the reviewer for insightful advice. In the present study, BMDMs and PBMCs were used for the matching in vivo experiment because virus or Poly(I:C) was injected intravenously. In the same way, PMs were used for in vitro bacterial infections due to the intraperitoneal injection of bacteria into mice. RAW264.7 and HEK293T cells were the commonly shared experimental model in the studies on innate immunity, which was to assess the effect of MARCH2 in immune cells as well as epithelial cells, respectively. However, we employed HeLa cells only for confocal microscopy because of the technical problem of HEK293T that can be easily detached in the washing step. Additionally, we also used A549 cells alternatively for some confocal microscopy due to the different expression of TLR receptors on the surface of HeLa and A549.

Comment)

Supplementary figure 1 feels unnecessary and a poor justification for selecting 'MARCH2 as a primary target' (as several other family members have the same effect)

Response)

We thank the reviewer's suggestion as well as apologize for the text making misunderstanding. We have removed it from the manuscript to resolve the concern from the

reviewer. We gave a solid and proper justification in the revised manuscript Line 112-113.

Comment)

Lower exposure of K48 ubiquitin blots in several figures is desirable

Response)

We have added lower exposure blots on Figure5F, and 5G. The revised manuscript now contains new K48 ubiquitin blots.

Comment)

Figure 4e,f: why all of a sudden these experiments are done with NDF virus, while all previous experiments were done with VSV, HSV or PR-8? Yet PR-8 is used again in 5c and VSV in figure 6.

Response)

In the overall study, we have evaluated the function of MARCH2 with several RNA and DNA viruses on innate immunity. That was the reason why we were intended to show if MARCH2 can affect verity of signaling pathways, results of which finally made us have a conclusion that NEMO could be a target. However, the virus strains used in those experiments were GFP-conjugated, which would not be able to be used for confocal microscopy in our hardware environment (due to the limitation in usable channel). To overcome this, we have employed wild type NDV for confocal microscopy and used NDV-GFP for binding assay to see the interaction between MARCH2 and NEMO. Other than NDV-GFP we used HSV-GFP virus for binding assay to see the interaction between MARCH2 and NEMO (Original manuscript Fig. S10a, Revised manuscript Appendix Figure S5B). Nonetheless, we also performed an additional binding assay using PR8-GFP infection to make the same phase with other experiments and now it's shown in the revised manuscript Appendix Figure S5A.

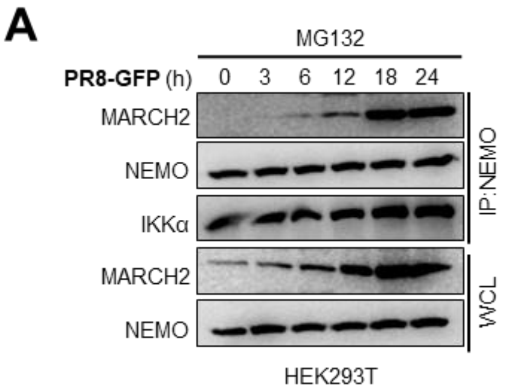

Appendix Figure S5A

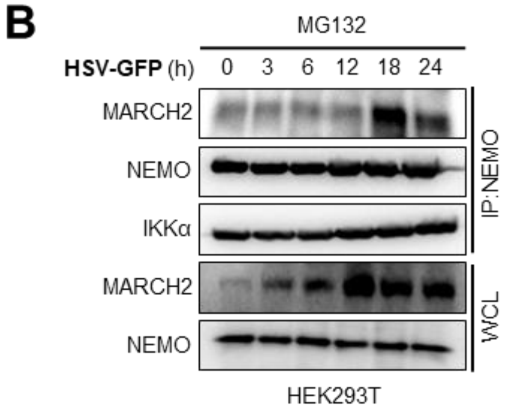

Appendix Figure S5B

Comment)

Supplementary figure 9b-d requires a MARCH2 pull-down western blot and preferably IKKalpha-beta-gamma pull-down on the same blot for a proper comparison. It looks like IKKalpha is also pulled-down by MARCH2.

Response)

We thank the reviewer's point. As indicated, we have performed binding assay between the molecules. The revised figure (Expanded View Figure 3B) is now providing a solid evidence that MARCH2 cannot interact with IKKα or IKKβ in absence of NEMO.

Reviewer's concern is further supported by the data in Expanded View Figure 5 C and D, which we conducted to explain one of reviewer 01's comment

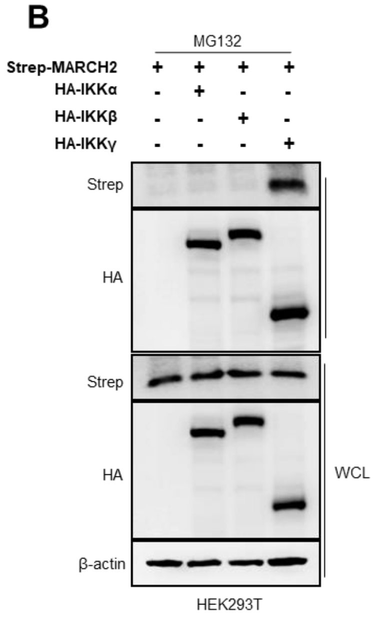

Expanded View Figure 3B

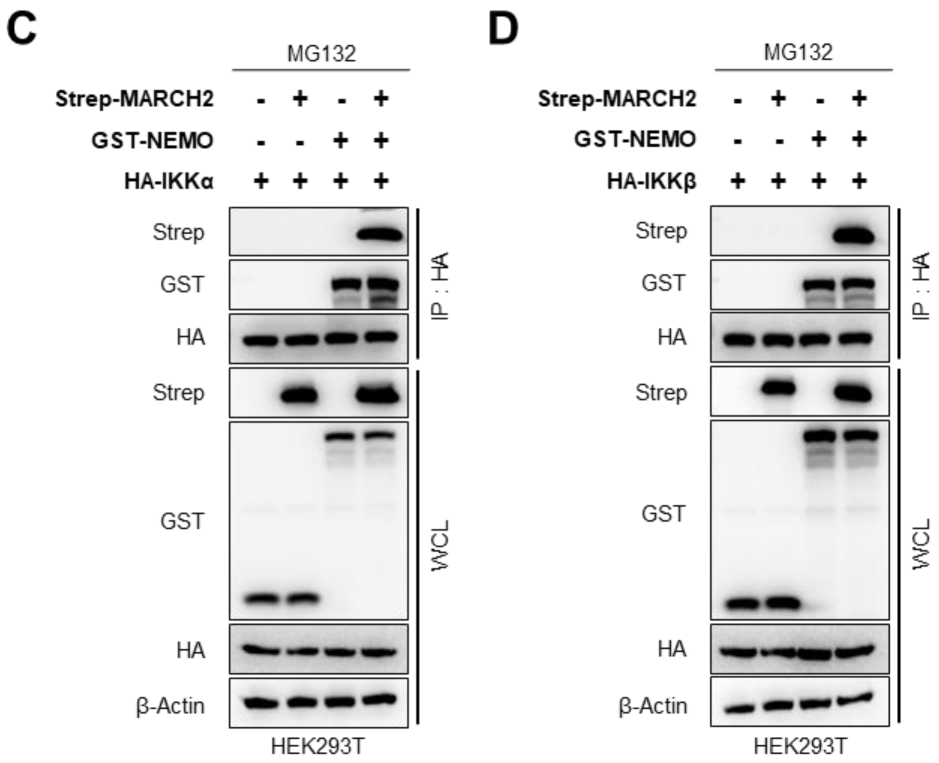

Expanded View Figure 5C and D

1st Revision - Editorial Decision 28th Jul 2020

Dear Prof. Lee,

Thank you for submitting your revise manuscript to the EMBO Journal. Your study has now been seen by the two referees and their comments are provided below. Both referees appreciate the introduced changes and support publication here.

As referee #2 points out would be great if you could carefully over the manuscript text and make sure that it reads well.

When you submit the revised version will you also take care of the following issues:

-I think would be good if we could have the figures in another format as power point like TIF. Is that possible? I don't know if you generated the files in power point.

- Can you please double check that the HA PD and WCL panels in figure EV3B are different blots as they look close to identical.

- The synopsis image looks good - the size should be 550 wide by [200-400] high (pixels). Could you double check that it is the right size.

- Figure call out to Appendix Figure S6 + S7 callout is missing the word "Figure".

- Call out to Appendix Table S2 is missing.

- Please double check that the images have scale bars (2C 5H, 7D, EV2A, S2, S3, S4A, S8C)

- The source data needs to be split in one file per figure. I think it would be good to separate the source data for the graphs and blots. If you have multiple files per figure please zip them together.

- I have asked our publisher to do their pre-publication checks on the paper. They will send me the file within the next few days. Please wait to upload the revised version until you have received their comments.

That should be all. You can use the link below to upload the revised version.

Best Karin

Karin Dumstrei, PhD
Senior Editor
The EMBO Journal

Instructions for preparing your revised manuscript:

Please check that the title and abstract of the manuscript are brief, yet explicit, even to non-specialists.

When assembling figures, please refer to our figure preparation guideline in order to ensure proper formatting and readability in print as well as on screen:
http://bit.ly/EMBOPressFigurePreparationGuideline

IMPORTANT: When you send the revision we will require
- a point-by-point response to the referees' comments, with a detailed description of the changes made (as a word file).
- a word file of the manuscript text.
- individual production quality figure files (one file per figure)
- a complete author checklist, which you can download from our author guidelines (https://www.embopress.org/page/journal/14602075/authorguide).
- Expanded View files (replacing Supplementary Information)
Please see out instructions to authors
https://www.embopress.org/page/journal/14602075/authorguide#expandedview

Please remember: Digital image enhancement is acceptable practice, as long as it accurately represents the original data and conforms to community standards. If a figure has been subjected to significant electronic manipulation, this must be noted in the figure legend or in the 'Materials and Methods' section. The editors reserve the right to request original versions of figures and the original images that were used to assemble the figure.

Further information is available in our Guide For Authors:
https://www.embopress.org/page/journal/14602075/authorguide

The revision must be submitted online within 90 days; please click on the link below to submit the revision online before 26th Oct 2020.

Link Not Available

Referee #1:

The reviewers have adequately addressed my comments and concerns. Even though it seems difficult in the experimental setup to assess, if ubiquitination at K326 by MARCH2 is fully responsible for NEMO degradation after viral infection, the data on MARCH2-triggered NEMO degradation are much stronger. Thus, the link of the physiological and mechanistic parts has been strongly improved.

Referee #2:

The authors have addressed all my comments and incorporated the necessary changes in the revised manuscript. I am fine with the results and conclusions. However, the manuscript will need significant editing for proper English writing by a native English speaking person. I also still think the discussion section is very repetitive with the results section (only minor efforts were made to improve this in the revised manuscript). Also the other reviewer mentioned that the manuscript can

be shortened and better streamlined.

Responses to comments (comments in **black,** responses in **blue,** changes in the text are marked in **red**.**)**

<u>(Comments for the Author):</u>

Comment)

As referee #2 points out would be great if you could carefully over the manuscript text and make sure that it reads well.

Response)

Now we have consulted an editing company and the manuscript has been edited by an immunology specialist who is a native English speaker. Moreover, we have carefully read the discussion section and made our every effort to avoid repetition and make the discussion more streamlined. Mainly we removed information on MARCH2 protein functional studies from discussion that were explain in the introduction section.

Comment)

I think would be good if we could have the figures in another format as power point like TIF. Is that possible? I don't know if you generated the files in power point.

Response)

In our first revision submission, we uploaded figure files in PowerPoint format. Now we uploaded our figures (07) and Expanded view figures (05) in TIFF format.

Comment)

Can you please double check that the HA PD and WCL panels in figure EV3B are different blots as they look close to identical?

Response)

We would like to thank for highlighting the point. HA-PD and WCL panels are two separate panels. Both samples were loaded in the same gel and blotted with anti-HA antibody. Un-cropped western blot image is given below.

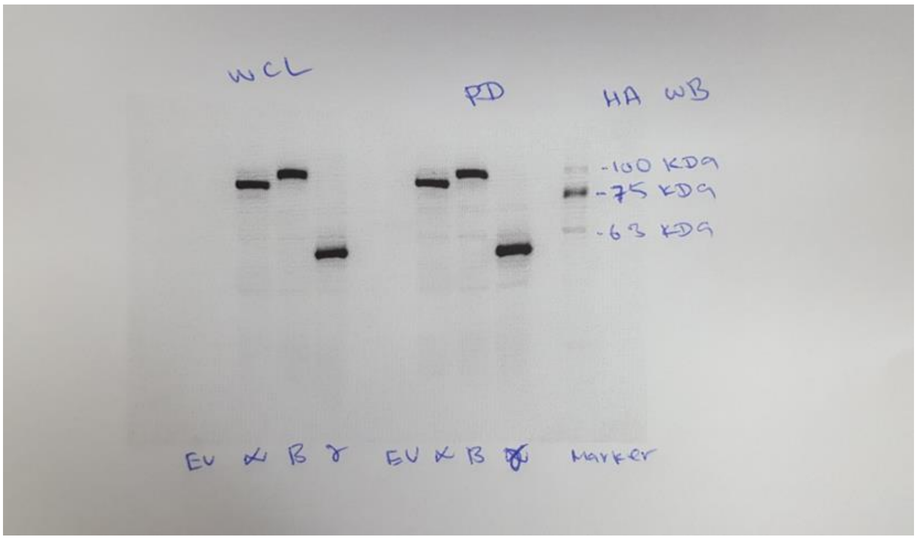

Comment)

The synopsis image looks good - the size should be 550 wide by [200-400] high (pixels). Could you double check that it is the right size.

Response)

Thank you. The size of the synopsis image is 550 x 332 pixels.

Comment)

Figure call out to Appendix Figure S6 + S7 callout is missing the word "Figure".

Response)

Thank you for the comment. We corrected the mistake. (Line 285)

Comment)

Call out to Appendix Table S2 is missing.

Response)

Thank you for the comment. We corrected the mistake. (Line 634)

Comment)

Please double check that the images have scale bars (2C 5H, 7D, EV2A, S2, S3, S4A, S8C)

Response)

Thank you for the attentive comment. Now we have carefully checked all the figures and added scale bars to missed images and the size of the scale bar is given in the figure legend.

Comment)

The source data needs to be split in one file per figure. I think it would be good to separate the source data for the graphs and blots. If you have multiple files per figure please zip them together.

Response)

Thank you. We make source data as one file per figure. We arranged the data for graphs and blots separately. When there are two files per one figure they were zipped together and kept as one file.

Reviewer #1 (Comments for the Author):

The reviewers have adequately addressed my comments and concerns. Even though it seems difficult in the experimental setup to assess, if ubiquitination at K326 by MARCH2 is fully responsible for NEMO degradation after viral infection, the data on MARCH2-triggered NEMO degradation are much stronger. Thus, the link of the physiological and mechanistic parts has been strongly improved.

Response)

We would like to thank and appreciate the support given by the reviewer to improve our manuscript.

Reviewer #2 (Comments for the Author):

The authors have addressed all my comments and incorporated the necessary changes in the revised manuscript. I am fine with the results and conclusions. However, the manuscript will need significant editing for proper English writing by a native English speaking person. I also still think the discussion section is very repetitive with the results section (only minor efforts were made to improve this in the revised manuscript). Also the other reviewer mentioned that the manuscript can be shortened and better streamlined.

Response)

We would like to thank and appreciate the support given by the reviewer to improve our manuscript. Now we have consulted an editing company and manuscript has been edited by an immunology specialist who is a native English speaker. As evidence we have attached the

editing certificate below. Moreover, we have carefully read the discussion section and made our every effort to avoid repetition and make the discussion more streamlined. Mainly we removed information on MARCH2 protein functional studies from discussion that were explain in the introduction section.

2nd Revision - Editorial Decision 11th Aug 2020

Dear Jong-Soo,

Thanks for sending us your revised manuscript. I have now had a chance to take a look at it and everything looks good.

I am therefore very pleased to accept the manuscript for publication here.

Congratulations on a nice study!

Best Karin

Karin Dumstrei, PhD
Senior Editor
The EMBO Journal

Please note that it is EMBO Journal policy for the transcript of the editorial process (containing referee reports and your response letter) to be published as an online supplement to each paper. If you do NOT want this, you will need to inform the Editorial Office via email immediately. More information is available here: http://emboj.embopress.org/about#Transparent_Process

Your manuscript will be processed for publication in the journal by EMBO Press. Manuscripts in the PDF and electronic editions of The EMBO Journal will be copy edited, and you will be provided with page proofs prior to publication. Please note that supplementary information is not included in the proofs.

Should you be planning a Press Release on your article, please get in contact with embojournal@wiley.com as early as possible, in order to coordinate publication and release dates.

If you have any questions, please do not hesitate to call or email the Editorial Office. Thank you for your contribution to The EMBO Journal.

EMBO PRESS

YOU MUST COMPLETE ALL CELLS WITH A PINK BACKGROUND ➔

PLEASE NOTE THAT THIS CHECKLIST WILL BE PUBLISHED ALONGSIDE YOUR PAPER

Corresponding Author Name: Jong-Soo Lee
Journal Submitted to: EMBO Journal
Manuscript Number: EMBOJ-2020-105139

USEFUL LINKS FOR COMPLETING THIS FORM

http://www.antibodypedia.com	Antibodypedia
http://1degreebio.org	1DegreeBio
http://www.equator-network.org/reporting-guidelines/improving-bioscience-research-report	ARRIVE Guidelines
http://grants.nih.gov/grants/olaw/olaw.htm	NIH Guidelines in animal use
http://www.mrc.ac.uk/Ourresearch/Ethicsresearchguidance/Useofanimals/index.htm	MRC Guidelines on animal use
http://ClinicalTrials.gov	Clinical Trial registration
http://www.consort-statement.org	CONSORT Flow Diagram
http://www.consort-statement.org/checklists/view/32-consort/66-title	CONSORT Check List
http://www.equator-network.org/reporting-guidelines/reporting-recommendations-for-tumo	REMARK Reporting Guidelines (marker prognostic studies)
http://datadryad.org	Dryad
http://figshare.com	Figshare
http://www.ncbi.nlm.nih.gov/gap	dbGAP
http://www.ebi.ac.uk/ega	EGA
http://biomodels.net/	Biomodels Database
http://biomodels.net/miriam/	MIRIAM Guidelines
http://jjj.biochem.sun.ac.za	JWS Online
http://oba.od.nih.gov/biosecurity/biosecurity_documents.html	Biosecurity Documents from NIH
http://www.selectagents.gov/	List of Select Agents

Reporting Checklist For Life Sciences Articles (Rev. June 2017)

This checklist is used to ensure good reporting standards and to improve the reproducibility of published results. These guidelines are consistent with the Principles and Guidelines for Reporting Preclinical Research issued by the NIH in 2014. Please follow the journal's authorship guidelines in preparing your manuscript.

A- Figures

1. Data

The data shown in figures should satisfy the following conditions:

- the data were obtained and processed according to the field's best practice and are presented to reflect the results of the experiments in an accurate and unbiased manner.
- figure panels include only data points, measurements or observations that can be compared to each other in a scientifically meaningful way.
- graphs include clearly labeled error bars for independent experiments and sample sizes. Unless justified, error bars should not be shown for technical replicates.
- if n< 5, the individual data points from each experiment should be plotted and any statistical test employed should be justified
- Source Data should be included to report the data underlying graphs. Please follow the guidelines set out in the author ship guidelines on Data Presentation.

2. Captions

Each figure caption should contain the following information, for each panel where they are relevant:

- a specification of the experimental system investigated (eg cell line, species name).
- the assay(s) and method(s) used to carry out the reported observations and measurements
- an explicit mention of the biological and chemical entity(ies) that are being measured.
- an explicit mention of the biological and chemical entity(ies) that are altered/varied/perturbed in a controlled manner.
- the exact sample size (n) for each experimental group/condition, given as a number, not a range;
- a description of the sample collection allowing the reader to understand whether the samples represent technical or biological replicates (including how many animals, litters, cultures, etc.).
- a statement of how many times the experiment shown was independently replicated in the laboratory.
- definitions of statistical methods and measures:
  - common tests, such as t-test (please specify whether paired vs. unpaired), simple χ2 tests, Wilcoxon and Mann-Whitney tests, can be unambiguously identified by name only, but more complex techniques should be described in the methods section;
  - are tests one-sided or two-sided?
  - are there adjustments for multiple comparisons?
  - exact statistical test results, e.g., P values = x but not P values < x;
  - definition of 'center values' as median or average;
  - definition of error bars as s.d. or s.e.m.

Any descriptions too long for the figure legend should be included in the methods section and/or with the source data.

In the pink boxes below, please ensure that the answers to the following questions are reported in the manuscript itself. Every question should be answered. If the question is not relevant to your research, please write NA (non applicable). We encourage you to include a specific subsection in the methods section for statistics, reagents, animal models and human subjects.

B- Statistics and general methods

Please fill out these boxes ➔ (Do not worry if you cannot see all your text once you press return)

1.a. How was the sample size chosen to ensure adequate power to detect a pre-specified effect size?	Standard protocols were followed. Sample numbers (n) for each data were detailed in figure legends.
1.b. For animal studies, include a statement about sample size estimate even if no statistical methods were used.	We determined the reasonable sample size by applying formal statistical power based on pilot experiments. In general we used 5-8 mice per genotype and condition(Virus and bacteria infection/LPS treatment for survival assay). Number of independent replication and biological replications are provided in each figure legend. The attempts at replication were successful .
2. Describe inclusion/exclusion criteria if samples or animals were excluded from the analysis. Were the criteria pre-established?	No data were excluded from the analyses.
3. Were any steps taken to minimize the effects of subjective bias when allocating animals/samples to treatment (e.g. randomization procedure)? If yes, please describe.	No randomization was used in this study.
For animal studies, include a statement about randomization even if no randomization was used.	No randomization was used in this study.
4.a. Were any steps taken to minimize the effects of subjective bias during group allocation or/and when assessing results (e.g. blinding of the investigator)? If yes please describe.	No.
4.b. For animal studies, include a statement about blinding even if no blinding was done	Studies was conducted with no blinding as conclusions do not rely on subjective measures.
5. For every figure, are statistical tests justified as appropriate?	The statistical tests used is specified in each figure. The justification is provided in the Materials and Methods section.
Do the data meet the assumptions of the tests (e.g., normal distribution)? Describe any methods used to assess it.	Performed Stastiscal analysis are described in the figure legends.
Is there an estimate of variation within each group of data?	No
Is the variance similar between the groups that are being statistically compared?	NA

C- Reagents

6. To show that antibodies were profiled for use in the system under study (assay and species), provide a citation, catalog number and/or clone number, supplementary information or reference to an antibody validation profile. e.g., Antibodypedia (see link list at top right), 1DegreeBio (see link list at top right).	Following antibodies were used for immuno-blotting or immunofluorescence experiments.<br>(Antibody/clone/manufacture/catalog#/source/dilution)<br>01. Antibodies used for immuno-blotting experiments<br>MARCH2,home,rabbite polyclonal,1:200<br>NEMO (DA10-12),Cell Signaling Technology,2695,Mouse monoclonal,1:300<br>K48-Ubi,Millipore,05-1305,Rabbit monoclonal,1:1000<br>K63-Ubi,Millipore,05-1308,Rabbit monoclonal,1:1000<br>IKKα,Santa Cruz Biotechnology,sc-7606,Mouse polyclonal,1:1000<br>IKKβ (D30C6),Cell Signaling Technology,8943S,Rabbit monoclonal,1:1000<br>HA-Probe(Y-11),Santa Cruz,sc-805,Rabbit polyclonal,1:3000<br>FLAG,Sigma,F1804,Mouse monoclonal,1:3000<br>GST (26H1),Cell Signaling Technology,2624S,Mouse monoclonal,1:3000<br>Strep,,IBA,2-1509-001,Mouse monoclonal,1:5000<br>P-IRF3 (S396) (4D4G),Cell Signaling Technology,4947S,Rabbit monoclonal,1:1000<br>IRF3 (D83B9),Cell Signaling Technology,4302S,Rabbit monoclonal,1:1000<br>P-P65 (Ser536) (93H1),Cell Signaling Technology,3033S,Rabbit monoclonal,1:1000<br>P65 (C22B4),Cell Signaling Technology,4764,Rabbit monoclonal,1:1000<br>P-TBK1 (Ser172) (D52C2),Cell Signaling Technology,5483S,Rabbit monoclonal,1:1000<br>[illegible]

	P-IκBα (Ser32) (14D4),Cell Signaling Technology,2859S,Rabbit monoclonal,1:1000<br>IκBα,Cell Signaling Technology,9242S,Rabbit monoclonal,1:1000<br>β-actin,BD Biosciences,610153,Mouse monoclonal,1:3000<br>HRP-conjugated anti-mouse IgG,Gene Tex GTX,213111-01,Goat,1:3000<br>HRP-conjugated anti-rabbit IgG,Cell Signaling Technology,7074S,Goat,1:3000<br>02. Antibodies used for immunofluorescence experiments<br>MARCH2,home made,Rabbit polyclonal,1:50<br>NEMO (DA10-12),Cell Signaling Technology,2695,Mouse monoclonal,1:200<br>Cy3-conjugated donkey anti-mouse IgG,The Jackson Laboratory,715-165-150,Mouse,1:400<br>Alexa 488 goat anti-rabbit IgG,Invitrogen,A11034,Rabbit,1:400<br>ER-Maker-Calnexin(E-10), Santa Cruz Biotechnology, Sc46669, Mouse monoclonal,1:50
7. Identify the source of cell lines and report if they were recently authenticated (e.g., by STR profiling) and tested for mycoplasma contamination.	Following cell lines were purchased from ATCC and used in this study<br>-HEK293T (ATCC-11268)<br>-Raw264.7 (ATCC TIB-71)<br>-HeLa (ATCC CCL-2)<br>-Vero (ATCC CCL-81)<br>-A549 (ATCC CCL-185)<br>-MARCH2 knock out HEK293T and IRES, MARCH2 or MARCH2 mutant over expressing Raw264.7 cells were produced as described in the manuscript<br>-Bone Marrow-Derived Macrophage (BMDM), Peripheral blood mononuclear cell (PBMC), Peritoneal Macrophage (PM) from MARCH2+/+ of MARCH2-/- mice were isolated and cultured as described in the manuscript. None of the cell line used were authenticated by ourselves. All cell lines were free of mycoplasma contamination.

* for all hyperlinks, please see the table at the top right of the document

D- Animal Models

8. Report species, strain, gender, age of animals and genetic modification status where applicable. Please detail housing and husbandry conditions and the source of animals.	C57B/6 J strain mice were used in this study. Both male and female animals were used for experiments. Details and reference for MARCH2 knockout mice generation and genotyping are described in the methods section. 6-8 weeks old animals were used for most experiments as specified in the text. All animal experiments were performed in bio-safety level BSL-2 laboratory facilities with the Guide for the Care and Use of Laboratory Animals (published by the US National Institutes of Health). No wild animals (mice) and no field-collected samples were used in this study.
9. For experiments involving live vertebrates, include a statement of compliance with ethical regulations and identify the committee(s) approving the experiments.	All animal experiments were approved by the Institutional Animal Use and Care Committee of Chungnam National University (Reference number CNU-00813, CNU-00921 and CNU-00927).
10. We recommend consulting the ARRIVE guidelines (see link list at top right) (PLoS Biol. 8(6), e1000412, 2010) to ensure that other relevant aspects of animal studies are adequately reported. See author guidelines, under 'Reporting Guidelines'. See also: NIH (see link list at top right) and MRC (see link list at top right) recommendations. Please confirm compliance.	We have complied with all guidelines to correctly report animal studies.

E- Human Subjects

11. Identify the committee(s) approving the study protocol.	NA
12. Include a statement confirming that informed consent was obtained from all subjects and that the experiments conformed to the principles set out in the WMA Declaration of Helsinki and the Department of Health and Human Services Belmont Report.	NA
13. For publication of patient photos, include a statement confirming that consent to publish was obtained.	NA
14. Report any restrictions on the availability (and/or on the use) of human data or samples.	NA
15. Report the clinical trial registration number (at ClinicalTrials.gov or equivalent), where applicable.	NA
16. For phase II and III randomized controlled trials, please refer to the CONSORT flow diagram (see link list at top right) and submit the CONSORT checklist (see link list at top right) with your submission. See author guidelines, under 'Reporting Guidelines'. Please confirm you have submitted this list.	NA
17. For tumor marker prognostic studies, we recommend that you follow the REMARK reporting guidelines (see link list at top right). See author guidelines, under 'Reporting Guidelines'. Please confirm you have followed these guidelines.	NA

F- Data Accessibility

18: Provide a "Data Availability" section at the end of the Materials & Methods, listing the accession codes for data generated in this study and deposited in a public database (e.g. RNA-Seq data: Gene Expression Omnibus GSE39462, Proteomics data: PRIDE PXD000208 etc.) Please refer to our author guidelines for 'Data Deposition'.<br><br>Data deposition in a public repository is mandatory for:<br>a. Protein, DNA and RNA sequences<br>b. Macromolecular structures<br>c. Crystallographic data for small molecules<br>d. Functional genomics data<br>e. Proteomics and molecular interactions	The data that support the findings of this study are available from the corresponding author upon reasonable request<br>Full scan unprocessed westernblot data are provided with source data file<br>NCBI reference sequence for Human MARCH2 https://www.ncbi.nlm.nih.gov/nuccore/BC032624.1,<br>NCBI reference sequence for Human NEMO https://www.ncbi.nlm.nih.gov/nuccore/NM_003639.4
19. Deposition is strongly recommended for any datasets that are central and integral to the study; please consider the journal's data policy. If no structured public repository exists for a given data type, we encourage the provision of datasets in the manuscript as a Supplementary Document (see author guidelines under 'Expanded View' or in unstructured repositories such as Dryad (see link list at top right) or Figshare (see link list at top right).	NA
20. Access to human clinical and genomic datasets should be provided with as few restrictions as possible while respecting ethical obligations to the patients and relevant medical and legal issues. If practically possible and compatible with the individual consent agreement used in the study, such data should be deposited in one of the major public access-controlled repositories such as dbGAP (see link list at top right) or EGA (see link list at top right).	NA
21. Computational models that are central and integral to a study should be shared without restrictions and provided in a machine-readable form. The relevant accession numbers or links should be provided. When possible, standardized format (SBML, CellML) should be used instead of scripts (e.g. MATLAB). Authors are strongly encouraged to follow the MIRIAM guidelines (see link list at top right) and deposit their model in a public database such as Biomodels (see link list at top right) or JWS Online (see link list at top right). If computer source code is provided with the paper, it should be deposited in a public repository or included in supplementary information.	Confocal microscopy data were analyzed using NIS-Elements software(NISE-4.20-b972)<br>Statistical analysis was performed using Prism 6 (GraphPad Software),https://www.graphpad.com/

G- Dual use research of concern

22. Could your study fall under dual use research restrictions? Please check biosecurity documents (see link list at top right) and list of select agents and toxins (APHIS/CDC) (see link list at top right). According to our biosecurity guidelines, provide a statement only if it could.	No
