# Supplementary material for: Negative regulation of NEMO signaling by the ubiquitin E3 ligase MARCH2
Source: EMBO J. 2020 Sep 16;39(21):e105139. doi: 10.15252/embj.2020105139 (PMC7604578; doi:10.15252/embj.2020105139)

Fig EV4A

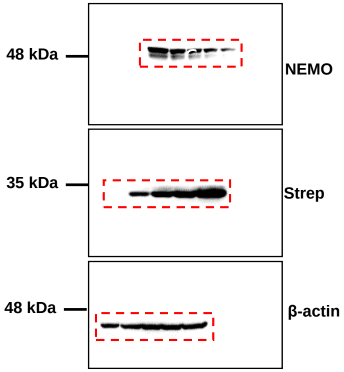

Fig EV4B

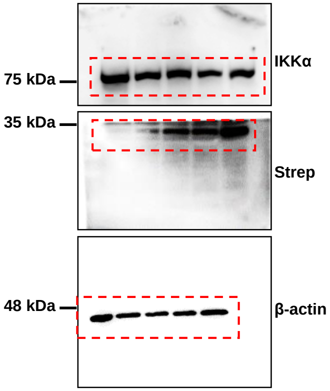

Fig EV4C

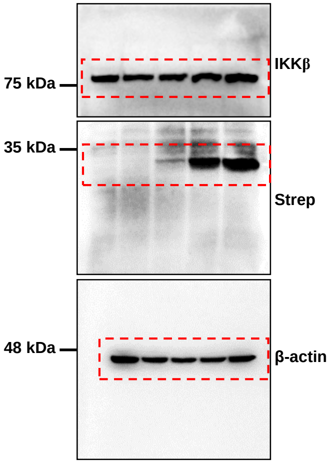

Fig EV4I

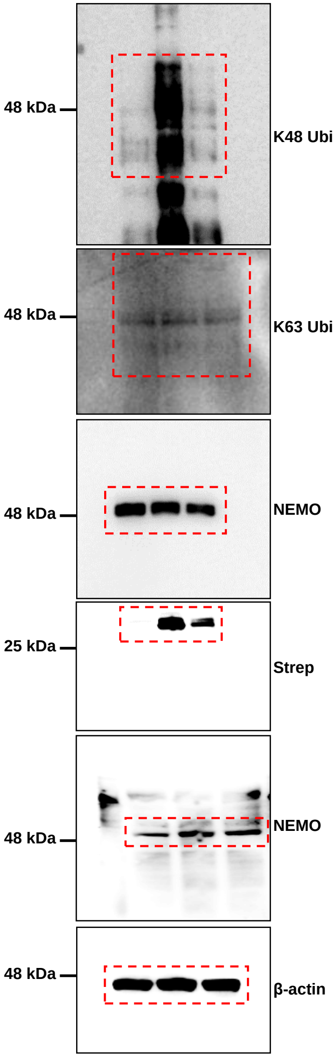

Fig EV4E

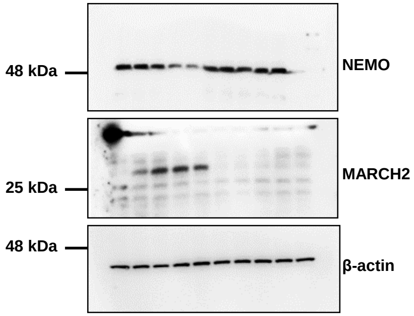

Fig EV4G

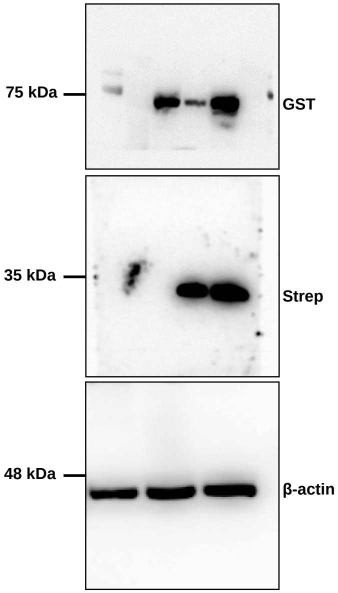

Fig EV4H

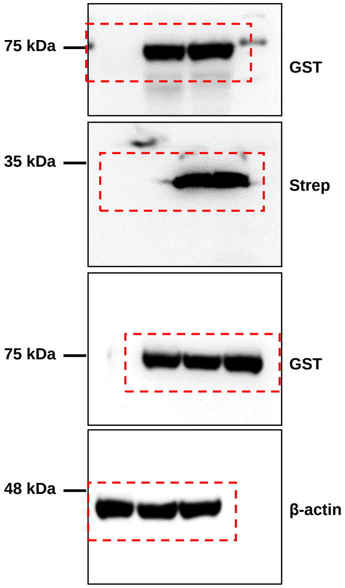

Fig EV4F

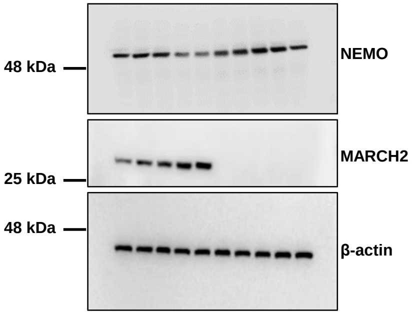

Supplement: Supplementary file 3 — Source Data for Expanded View and Appendix [file EMBJ-39-e105139-s010.zip › Appendix_and_EV_Figure_Source_Data/EV_figure_Source_Data/Source Data for Figure EV04.zip!/EMBOJ-2020-105139R-Source Data for Figure EV04.pdf]
